# Brown marmorated stink bug, *Halyomorpha halys* (Stål), genome: putative underpinnings of polyphagy, insecticide resistance potential and biology of a top worldwide pest

**DOI:** 10.1186/s12864-020-6510-7

**Published:** 2020-03-14

**Authors:** Michael E. Sparks, Raman Bansal, Joshua B. Benoit, Michael B. Blackburn, Hsu Chao, Mengyao Chen, Sammy Cheng, Christopher Childers, Huyen Dinh, Harsha Vardhan Doddapaneni, Shannon Dugan, Elena N. Elpidina, David W. Farrow, Markus Friedrich, Richard A. Gibbs, Brantley Hall, Yi Han, Richard W. Hardy, Christopher J. Holmes, Daniel S. T. Hughes, Panagiotis Ioannidis, Alys M. Cheatle Jarvela, J. Spencer Johnston, Jeffery W. Jones, Brent A. Kronmiller, Faith Kung, Sandra L. Lee, Alexander G. Martynov, Patrick Masterson, Florian Maumus, Monica Munoz-Torres, Shwetha C. Murali, Terence D. Murphy, Donna M. Muzny, David R. Nelson, Brenda Oppert, Kristen A. Panfilio, Débora Pires Paula, Leslie Pick, Monica F. Poelchau, Jiaxin Qu, Katie Reding, Joshua H. Rhoades, Adelaide Rhodes, Stephen Richards, Rose Richter, Hugh M. Robertson, Andrew J. Rosendale, Zhijian Jake Tu, Arun S. Velamuri, Robert M. Waterhouse, Matthew T. Weirauch, Jackson T. Wells, John H. Werren, Kim C. Worley, Evgeny M. Zdobnov, Dawn E. Gundersen-Rindal

**Affiliations:** 1 USDA-ARS Invasive Insect Biocontrol and Behavior Laboratory, Beltsville, MD 20705 USA; 2 USDA-ARS San Joaquin Valley Agricultural Sciences Center, Parlier, CA 93648 USA; 3 Department of Biological Sciences, University of Cincinnati, Cincinnati, OH 45221 USA; 4 Department of Human and Molecular Genetics, Human Genome Sequencing Center, Baylor College of Medicine, Houston, TX 77030 USA; 5 Department of Entomology, University of Maryland, College Park, MD 20742 USA; 6 Department of Biology, University of Rochester, Rochester, NY 14627 USA; 7 USDA-ARS National Agricultural Library, Beltsville, MD 20705 USA; 8 A.N. Belozersky Institute of Physico-Chemical Biology, Moscow State University, Moscow, 119911 Russia; 9 Department of Biological Sciences, Wayne State University, Detroit, MI 48201 USA; 10 Department of Biochemistry, Virginia Tech, Blacksburg, VA 24061 USA; 11 Department of Biology, Indiana University, Bloomington, IN 47405 USA; 12 Department of Genetic Medicine and Development, University of Geneva Medical School and Swiss Institute of Bioinformatics, 1211 Geneva, Switzerland; 13 Present address: Institute of Molecular Biology and Biotechnology, Foundation for Research and Technology-Hellas, 73100 Heraklion, Crete Greece; 14 Department of Entomology, Texas A&M University, College Station, TX 77843 USA; 15 Center for Genome Research and Biocomputing, Oregon State University, Corvallis, OR 97331 USA; 16 Center for Data-Intensive Biomedicine and Biotechnology, Skolkovo Institute of Science and Technology, Skolkovo, 143025 Russia; 17 National Center for Biotechnology Information, National Library of Medicine, National Institutes of Health, Bethesda, MD 20894 USA; 18 URGI, INRA, Université Paris-Saclay, 78026 Versailles, France; 19 Environmental Genomics and Systems Biology Division, Lawrence Berkeley National Laboratory, Berkeley, CA 94720 USA; 20 Department of Microbiology, Immunology and Biochemistry, University of Tennessee Health Science Center, Memphis, TN 38163 USA; 21 USDA-ARS Center for Grain and Animal Health Research, Manhattan, KS 66502 USA; 22 Developmental Biology, Institute for Zoology: University of Cologne, 50674 Cologne, Germany; 23 School of Life Sciences, University of Warwick, Gibbet Hill Campus, Coventry, CV4 7AL United Kingdom; 24 EMBRAPA Genetic Resources and Biotechnology, Brasília, DF 70770-901 Brazil; 25 Larner College of Medicine, The University of Vermont, Burlington, VT 05452 USA; 26 Present address: Earth BioGenome Project, University of California, Davis, Davis, CA 95616 USA; 27 Department of Entomology, University of Illinois, Urbana-Champaign, IL 61801 USA; 28 Department of Ecology and Evolution, University of Lausanne and Swiss Institute of Bioinformatics, 1015 Lausanne, Switzerland; 29 Division of Biomedical Informatics, and Division of Developmental Biology, Center for Autoimmune Genomics and Etiology, Cincinnati Children’s Hospital Medical Center, Cincinnati, OH 45229 USA; 30 Department of Pediatrics, College of Medicine, University of Cincinnati, Cincinnati, OH 45267 USA

**Keywords:** Brown marmorated stink bug genome, Pentatomid genomics, polyphagy, chemoreceptors, odorant binding proteins, opsins, cathepsins, xenobiotic detoxification, invasive species

## Abstract

**Background:**

*Halyomorpha halys* (Stål), the brown marmorated stink bug, is a highly invasive insect species due in part to its exceptionally high levels of polyphagy. This species is also a nuisance due to overwintering in human-made structures. It has caused significant agricultural losses in recent years along the Atlantic seaboard of North America and in continental Europe. Genomic resources will assist with determining the molecular basis for this species’ feeding and habitat traits, defining potential targets for pest management strategies.

**Results:**

Analysis of the 1.15-Gb draft genome assembly has identified a wide variety of genetic elements underpinning the biological characteristics of this formidable pest species, encompassing the roles of sensory functions, digestion, immunity, detoxification and development, all of which likely support *H. halys*’ capacity for invasiveness. Many of the genes identified herein have potential for biomolecular pesticide applications.

**Conclusions:**

Availability of the *H. halys* genome sequence will be useful for the development of environmentally friendly biomolecular pesticides to be applied in concert with more traditional, synthetic chemical-based controls.

## Background

*Halyomorpha halys* (Stål) (Heteroptera: Pentatomidae), the brown marmorated stink bug (BMSB), is native to Asia (China, Taiwan, Korea and Japan) and has emerged in recent decades as a major insect pest of worldwide importance due to its exceptional capacity to colonize new habitats (i.e., invasiveness). Accidentally introduced outside its native range, *H. halys* has become established in North America (Allentown, Pennsylvania, United States, mid-1990s), Europe (Zurich, Switzerland, 2007) and South America (Santiago, Chile, 2017) [1]; it has also been detected yet eradicated multiple times in Australia [2]. In regions where it has established, *H. halys’* high dispersal capacity, polyphagy (at least 170 plant species) and ability to compete with endemic species have assisted its spread (reviewed in [3]). In combination, these traits helped *H. halys* to spread quickly and cause significant agricultural losses, especially to specialty crops such as orchard fruits (apples, stone and pome), grapes, ornamental plants, vegetables, seed crops, as well as staple crops [4]. As *H. halys* continues to expand its range, it poses major threats to agriculture, especially to such staple crops as corn and soybean grown in the primary agricultural production regions of the American Midwest [5]. *H. halys* is also a nuisance pest, well known for its invasion of human structures such as houses, schools and other indoor spaces in large numbers when it overwinters [6].

**Fig. 1:**
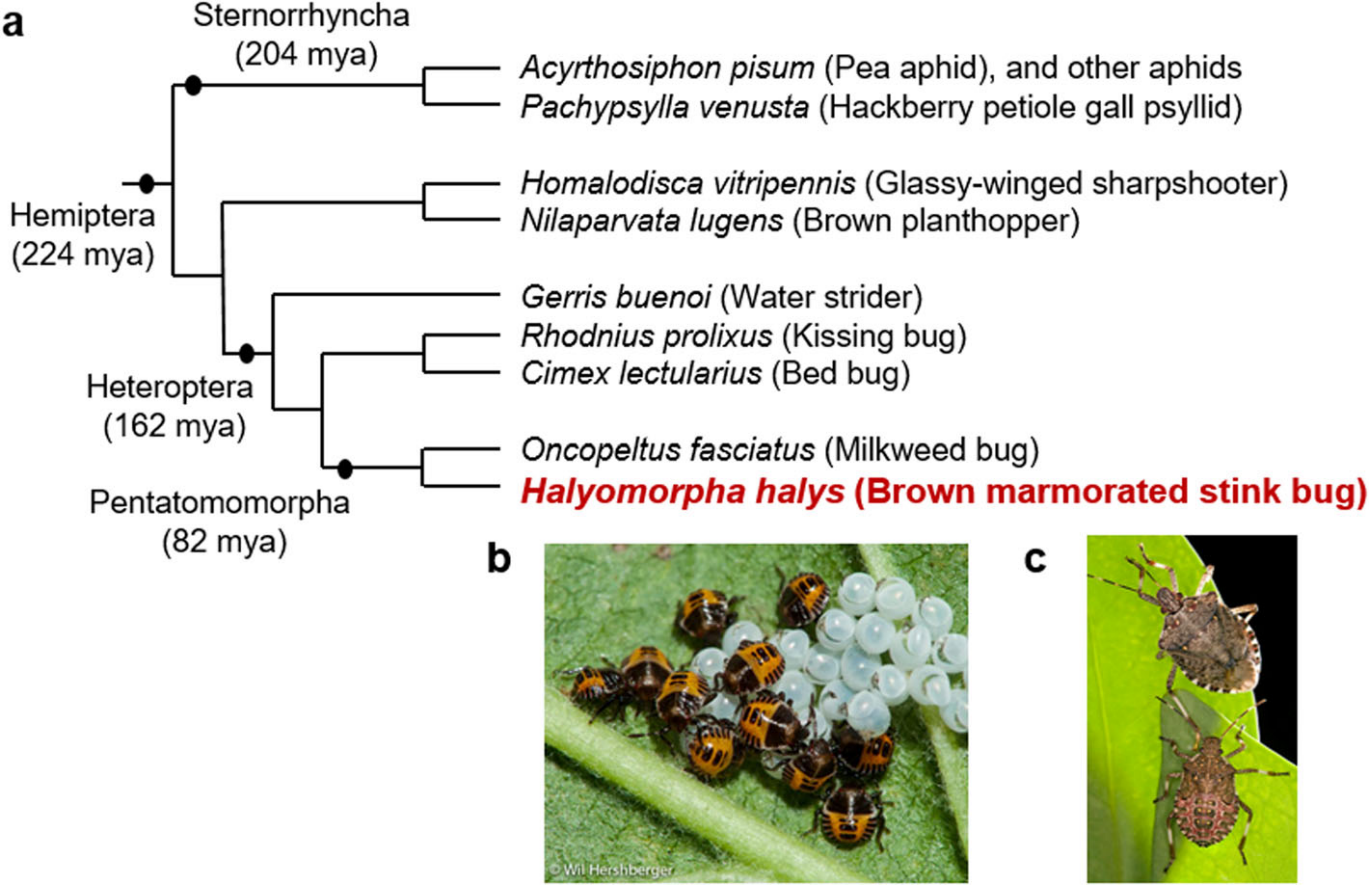
Genomic resources in the Hemiptera. **a** Phylogenetic relatedness of selected hemipterans with available full genomes (modified from [14], originally based on [15]). **b** *H. halys* nymphs, first instar, cluster around a mass of newly-hatched eggs on the underside of a leaf (photo from http://www.stopbmsb.org/ by W. Hershberger; used with permission). **c** Adult (top) and fifth-instar nymph (bottom).

*H. halys* is a member of the insect order Hemiptera, which contains approximately 82,000 described species and constitutes the most speciose order of hemimetabolous insects [7]. All hemipteran insects share a piercing-sucking mouthpart anatomy [8], but have diversified across a wide range of different food sources (including vertebrates). Five clades are recognized within the Hemiptera: Sternorrhyncha (scale insects, aphids, whiteflies and psyllids), Fulgoromorpha (planthoppers), Cicadomorpha (leafhoppers, spittlebugs and cicadas), Coleorrhyncha (moss bugs) and Heteroptera (true bugs) [9]. As a “true bug,” *H. halys* belongs to the sub-order Heteroptera, and to the family Pentatomidae, which encompasses all stink bugs (or shield bugs; see Additional file 1: Figure S1). This report provides the first complete Pentatomid genome, thus complementing previously published hemipteran genomes including a species of the kissing bugs, *Rhodnius prolixus* [10]; the pea aphid, *Acyrthosiphon pisum* [11]; the water strider, *Gerris buenoi* [12]; the brown plant hopper, *Nilaparvata lugens* (Fulgoromorpha) [13]; and the milkweed bug, *Oncopeltus fasciatus* [14]; among others (see Fig. 1).

Analysis of the *H. halys* genome was conducted as a community annotation project under the “i5K” initiative to sequence the genomes of 5,000 insects and other arthropods with important biological significance or economic value [16]. Given the significance of *H. halys* as a worldwide invasive pest, top priority was given to the annotation and analysis of gene families related to sensory functions, digestion, immunity, detoxification and development. These efforts revealed informative genome features potentially related to broad phytophagy (e.g., chemosensory genes), xenobiotic detoxification (with attendant potential to develop insecticide resistance) and digestion.

Numerous integrated pest management and biological control measures, as well as monitoring and targeted chemical control tactics, have been explored for *H. halys* [17, 18]. The genome sequence draft provided here—being the product of sequencing single female and male specimens following 10 generations of sibling-sibling mating—will help to dissect the genetic underpinnings of how *H. halys* is attracted to and infests new host plants, of its potential to develop insecticide resistance and possibly of its biological vulnerabilities, thereby assisting in the development of environmentally sustainable biomolecular pesticides for controlling this important pest.

## Results and Discussion

### Genome sequencing, assembly and annotation

The genome sequencing and assembly yielded an assembly of 1.15 Gb (1.00 Gb in gap-free scaffolds) with a contig N50 of 17.7 kb and scaffold N50 of 802 kb. The overall genome size was estimated to be 1.143 +/- 0.019 Gb (n= 4) and 1.095 +/- 0.023 Gb (n=4) for the female and male, respectively, using flow cytometry (see Additional file 1). The data have been deposited in the NCBI as Genbank assembly accession GCA_000696795.1. The Official Gene Set halhal_OGSv1.1, reflecting automated and manually annotated genes, comprises 24,450 protein-coding gene models.

The BUSCO completeness assessment tool [19, 20] searches assemblies and annotated gene sets for genes that are expected, based on comparisons to similar species, to be present as single-copy orthologs in order to assess completeness in terms of expected gene content. *H. halys* showed high levels of completeness both for the genome assembly (96.7%) and the annotated gene set (98.7%), missing only 29 and 13 of the 1,658 Insecta BUSCO genes, respectively (Table 1). This was supported by additional quality checks comparing orthologs with four other hemipterans which showed that *H. halys* has the highest representation in near-universal orthogroups and the lowest numbers of missing orthologs (see Additional file 1). Analysis of hemipteran ortholog distributions identified a conserved core of nearly 5,000 orthogroups with orthologs in *H. halys* and four other representative hemipterans (see Additional file 1: Table S2 and Figure S2). In support of overall assembly quality, appropriate assembly of the highly conserved Hox and Iro-C gene clusters—which are hallmarks of bilaterian [21] and insect [22–24] genomes, respectively—was observed. Single-copy gene models were recovered for all expected orthologs, with linkage of the Iro-C and substantial linkage of the Hox cluster (for Hox2/3/4 and for Hox5/6/7/8/9/10: see remarks in Additional file 1: Figure S4). The *H. halys* gene set is thus comparable to other hemipterans with high-quality sequenced and annotated genomes and provides a strong foundation for analyses of the *H. halys* protein-coding gene repertoire. Additional assessments of assembly quality were performed and are described in Additional file 1.

**Table 1:** BUSCO completeness assessments of the genome assemblies and predicted gene sets of *H. halys* and three other hemipterans

Species	*Halyomorpha halys*		*Acyrthosiphon pisum*		*Cimex lectularius*		*Rhodnius prolixus*	
Dataset	Assembly	Gene set	Assembly	Gene set	Assembly	Gene set	Assembly	Gene set
Version	Hhal_1.0	Hhal_1.0	v2.0	v2.1b	ClecH1	ClecH1.3	RproC3	RproC3.3
% Complete BUSCOs	96.7	98.7	94.0	95.9	99.1	95.8	96.6	90.3
Complete BUSCOs	1,604	1,636	1,558	1,589	1,642	1,588	1,602	1,590
of which single-copy	1,577	1,596	1,479	1,477	1,606	1,542	1,590	1,481
of which duplicated	27	40	79	112	36	46	12	17
Fragmented BUSCOs	25	9	26	19	5	35	28	95
Missing BUSCOs	29	13	74	50	11	35	28	65

### Lateral Gene Transfers in *Halyomorpha halys*

Lateral Gene Transfers (LGTs) from microbes into arthropod genomes were once thought rare or non-existent, but are now know to be relatively common [25]. *H. halys* shares a lineage-specific (infraorder Pentatomomorpha) LGT event with the milkweed bug, *Oncopeltus fasciatus*, of a cell wall degradation enzyme, endo-1,4-beta-mannosidase [26]. Strikingly, this bacterial-origin gene has subsequently expanded into a nine-member, multigene family in *H. halys* through a series of species-specific tandem duplications (Additional file 1: Figure S5). While hemipteran genomic resources for comparative analysis are growing [14], the Pentatominae in particular will benefit from greater sampling of additional species. Preliminarily, tBLASTn alignments of a recent, unpublished assembly for the fellow pentatominid *Euschistus heros* (GenBank accession GCA_003667255.1) does support a potential tandem expansion of mannosidase genes in this polyphagous lineage (see Additional file 1: Figure S6).

Using the same methods as in Panfilio et al. (2019) [26], we identified a set of five additional candidate LGT events in *H. halys* (see Additional file 1). These include two independent LGTs of *Wolbachia* ankyrin-repeat-bearing genes, one of which has expanded into a four-member gene family and the other of which has duplicated once. These genes all show clear expression in the stages tested: 2^nd^ and 4^th^ nymphal instars, and male and female adults. Another independent *Wolbachia* transfer appears to have occurred from the *Wolbachia* phage WO, also with subsequent gene duplication. It is yet another ankyrin-repeat protein and both copies show post-embryonic expression. *Wolbachia* are widespread intracellular bacteria that infect 40-70 percent of arthropod species [27, 28] and are common sources of lateral gene transfers into arthropods [25]. Additionally, two candidate LGTs were found that appear to be derived from *Candidatus Pantoea carbekii*, the primary bacterial symbiont of *H. halys* [29]: one from a ribonuclease III gene and the other with weak similarity to a cytosol aminopeptidase, although both of these show only trace gene expression. The evolutionary history of these LGT candidates and their possible functions in *H. halys* require further investigation, particularly in light of the multiple, independent expansions in copy number after the original integration events.

### Chemoreceptors: Odorant, Gustatory and Ionotropic Receptors

Insects depend on the members of three large families of chemoreceptors for the specificity and sensitivity of most of their senses of smell and taste [30, 31]. The Odorant Receptor (OR) and Gustatory Receptor (GR) families together form the insect chemoreceptor superfamily of seven-transmembrane-domain ligand-gated ion channels. The GR family is far older than the OR family, which evolved within the Insecta [32]. In contrast, the Ionotropic Receptors (IRs) are a variant family of the otherwise highly conserved and widespread ionotropic glutamate receptors. Insects have widely ranging gene family sizes, from single digits to over 400 genes per family, which largely correlate with the complexity of their chemical ecology [33]. We compared these three families in *H. halys* with those from other hemipterans with available genome sequences to detect whether any potential expansions or contractions may have occurred along the hemipteran lineage leading to *H. halys* (Table 2; see also Additional file 1). Although extant genomic resources are inadequate to determine whether the following observations are unique to *H. halys*, they nonetheless shed light on how this insect is distinctive vis-à-vis the reference taxa. Results indicate that although the IR family is of roughly comparable size, there appears to have been a slight expansion of the OR family, including three potential lineage-specific expansions (one of which includes 40 genes). Most remarkable, however, is a major potential expansion of the GR family, both in number of genes and the prevalence of alternative splicing yielding different isoforms from 63 of the 198 genes. This is among the largest GR families known in insects, even taking into account that 37 of them are pseudogenic, leaving 330 apparently functional GR proteins. This total exceeds the 215 genes encoding 245 proteins (219 of them intact) in the flour beetle *Tribolium castaneum* [34], and the 197-213 and 231 genes reported from the highly polyphagous moths *Helicoverpa armigera* and *Spodoptera frugiperda* [35–38]. The only insects with known larger GR families are the omnivorous cockroaches *Periplaneta americana* [39] and *Blattella germanica* [40] that can encode 522 and 545 GRs, respectively, while the extraordinarily polyphagous spider mite *Tetranychus urticae* has 689 genes [41]. This major putative expansion of the GR family is primarily due to an increase in the number of candidate bitter taste receptors, which are generally implicated in perception of plant compounds in phytophagous insects [36, 37] and therefore potentially associated with the remarkably wide host range of this plant-feeding bug.

**Table 2:** Numbers of chemoreceptor genes/proteins in three families in six hemipteroid insects, as well as *D. melanogaster* and the termite *Zootermopsis nevadensis* for comparison

Species	Odorant	Gustatory	Ionotropic
*Halyomorpha halys*	148/149	198/347	39/39
*Oncopeltus fasciatus*	120/121	115/169	37/37
*Rhodnius prolixus*	116/116	28/30	33/33
*Cimex lectularius*	48/49	24/36	30/30
*Acyrthosiphon pisum*	79/79	77/77	19/19
*Pediculus humanus*	12/13	6/8	14/14
*Drosophila melanogaster*	60/62	60/68	65/65
*Zootermopsis nevadensis*	70/70	87/90	150/150

### Odorant-binding proteins

Forty-eight odorant-binding protein (OBP) genes were identified in the genome of *H. halys*, which are expected to encode 58 proteins due to isoforms (Additional file 1: Table S12). These include the 30 previously identified OBPs [42] and an additional 28 OBPs, totaling 50 classic Cys-pattern and 8 Plus-C OBPs. Seven OBP were considered pseudogenes based on the lack of detection of constitutive expression by qPCR (Additional file 1: Table S12). OBP pseudogenes were identified in *Apis mellifera*, *Bombyx mori*, *Nasonia vitripennis*, *T. castaneum* and several *Drosophila* species [43, 44]. The number of identified putative HhalOBP genes is comparable to that identified in the genomes of *D. melanogaster*, with 51 [45–47] and *B. mori* [48], with 44. This is fewer than that found in the genomes of *Anopheles gambiae* [49–52], *Aedes aegypti* [52] and *N. vitripennis* [44], with 68, 66 and 90, respectively. On the other hand, this is more than was found in the genome of *A. mellifera*, with 21 [53], and in transcriptomes of the neotropical stink bugs *Euschistus heros* (25), *Chinavia ubica* (25) and *Dichelops melacanthus* (9) [54, 55].

*Halyomorpha halys* has a ratio of OR to OBP genes of 148:48, approximately 3:1. The ratio of OR:OBP genes has been quite variable among insects, but always with more OR genes than OBP genes. For example, for *D. melanogaster* and *An. gambiae* the ratio is 70:50 [47, 50], and for *A. mellifera* it is 170:21 [53].

**Fig. 2:**
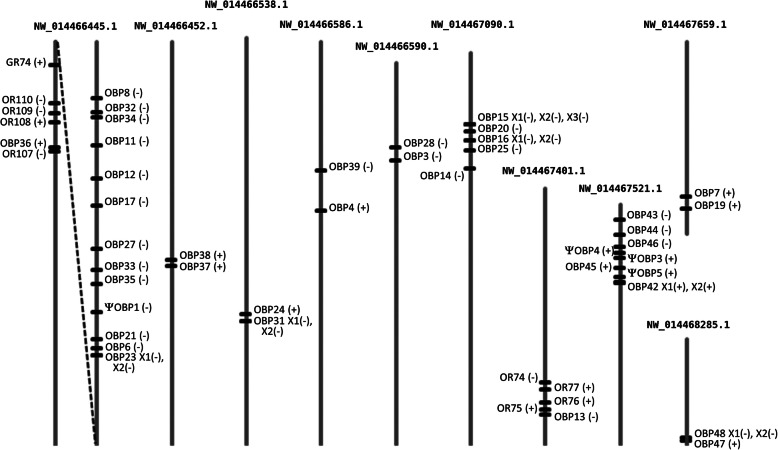
Locations of the OBP gene and pseudogene (Ψ) clusters in *H. halys* genome scaffolds. The location of each OBP gene is indicated by a horizontal line. Transcriptional directions are indicated by (+) for same direction as the scaffold or (-) for the opposite direction. NW_014466445.1 (2,775,865 bp) is the two terminal 750 k bp pieces, interconnected by a dashed line, which in one end has a cluster of OR genes with the gene *obp36* entirely placed inside an OR gene intron. NW_014467401.1 (475,337 bp) illustrates the close proximity of *obp13* to some OR genes. The 750 k bp terminal ends of scaffolds NW_014466452.1 (2,701,634 bp) and NW_014466586.1 (2,077,303 bp) are shown. The lengths of the other scaffolds are: NW_014466538.1 = 757,214 bp; NW_014466590.1 = 710,159 bp; NW_014467090.1 = 728,809 bp; NW_014467659.1 = 357,280 bp; and NW_014468285.1 = 196,245 bp. All scaffold representations are drawn to scale.

The HhalOBP genes and pseudogenes are distributed across 25 scaffolds. Forty are organized into nine clusters of 2-14 genes (Fig. 2), suggesting that most HhalOBP genes evolved by gene duplication. The largest cluster is in NW_014466445.1 with 13 of the 14 OBP genes organized in tandem in reverse orientation within ca 550 kb (Fig. 2). This cluster is separated by about 740 kb from a cluster of four ORs, one GR and one OBP gene (Additional file 1: Table S12). The second-largest cluster is in NW_014467521.1 with eight OBP genes organized within roughly 130 kb, with 5 and 3 in forward and reverse orientation, respectively. The third-largest cluster is in NW_014467090.1, with four OBP genes in tandem in reverse orientation within about 100 kb. The other OBP clusters have two OBP genes apiece. There was no evidence that a specific Cys-motif pattern was associated with any particular OBP cluster. Clustering of the majority of OBP genes is also seen in other insect genomes, such as *D. melanogaster*, *An. gambiae*, *A. mellifera, Ae. aegypti*, *B. mori* and *T. castaneum* [43, 47, 48, 50, 52, 53].

Two OBP genes not clustered with other OBPs call attention because of their location near ORs (Fig. 2). One is *obp13*, which was only 6 kb from the *or74* through *or77* genes in NW_014467401.1 (Fig. 2). Proximity between OR and OBP genes was also found in the genome of *Drosophila* [47], although no functional linkage has been discovered. The other gene is *obp36,* which per the draft assembly is entirely inside one OR gene intron, in a four-OR cluster with one GR gene close by. The finding of an OBP gene inside an OR intron is unprecedented.

The length of the HhalOBP genes ranged from 2,130 (*obp44*) to 37,085 (*obp21*) bases, which is longer than that found in, for example, the hemipteran aphids *A. pisum* [56] and *Aphis gossypii* [57], and the dipteran *Drosophila melanogaster* [47]. This is probably related to the larger and higher number of introns: four to eight introns ranging from 69 to 30,377 bases in *H. halys,* compared to zero to eight introns ranging from 58 to 12 kb in *A. pisum,* one to eight introns ranging from 0.6 to 2 kb for *A. gossypii* and zero to two introns ranging from zero to 638 bases in *D. melanogaster*.

### Vision and light detection genes

**Fig. 3:**
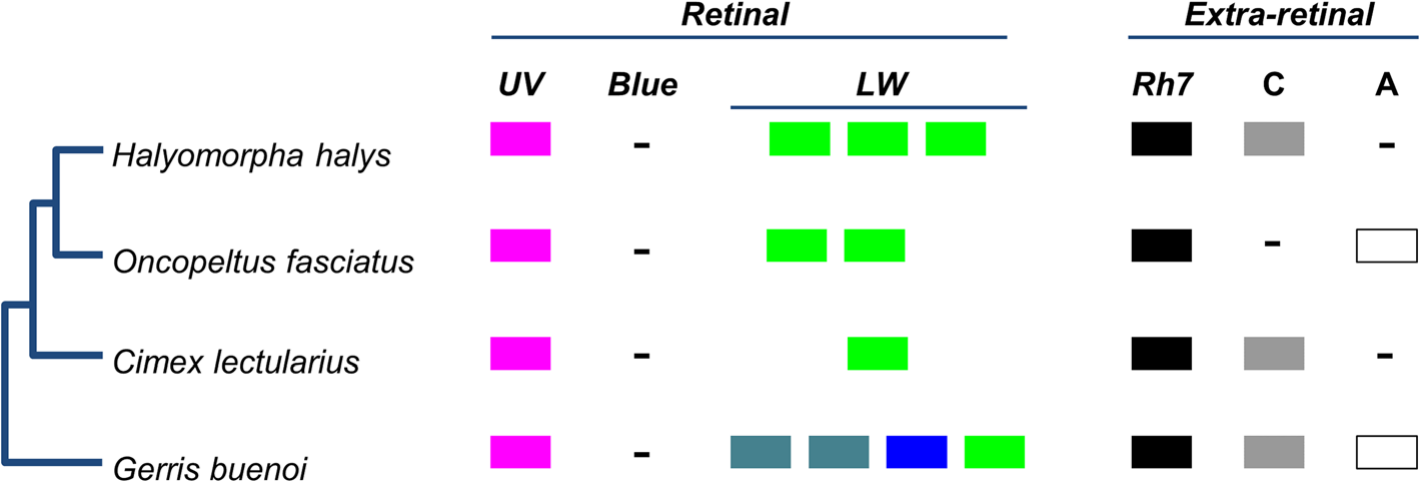
Opsin gene repertoire. Opsin gene family members detected in each of four heteropteran hemipterans surveyed is indicated [62]. C ~ ciliary opsins; A ~ arthropsins; as well as the four rhabdomeric opsins: UV ~ UV-sensitive, Blue ~ Blue-sensitive, LW ~ long wavelength-sensitive, and Rh7 ~ the Rh7 gene. Diverse LW opsin paralog colors indicate hypothesized wavelength-shifts based on [12].

Most heteropteran Hemiptera, including *H. halys*, are equipped with prominent lateral compound eyes and a set of smaller dorsal eyes, the ocelli [58]. By reference to *Drosophila* and previous comparative work on insect vision [59–61], this suggests the use of different opsin gene subfamilies expressed in the retinas of these visual organs to facilitate visual tasks in the context of flight dispersal, animal prey or food plant localization, predator avoidance and mate localization. Consistent with this expectation, the genomic opsin gene family surveys in *Cimex lectularius*, *O. fasciatus* and *G. buenoi* (water strider) uncovered varying representations of retinal opsin subfamilies (Fig. 3) [12, 22, 26]. Bed bugs are characterized by a single member of each of the UV and long wavelength (LW) sensitive opsin gene families [22], and a lack of blue sensitive (B) opsin genes, which constitute the third ancestral retinal opsin gene subfamily of insects. Singleton homologs of the UV-sensitive opsin gene family were also found in the water strider and the milkweed bug [12, 26]; as with the bed bug, no B opsin homologs were detected in the genome drafts or transcriptomes of either species, suggesting that the B opsin gene family was lost in the lineage to the last common ancestor of heteropteran Hemiptera [12]. Water strider and milkweed bug, however, differ from bed bugs by expanded LW opsin repertoires, possessing four and two members of this opsin gene subfamily, respectively [12, 26].

**Fig. 4:**
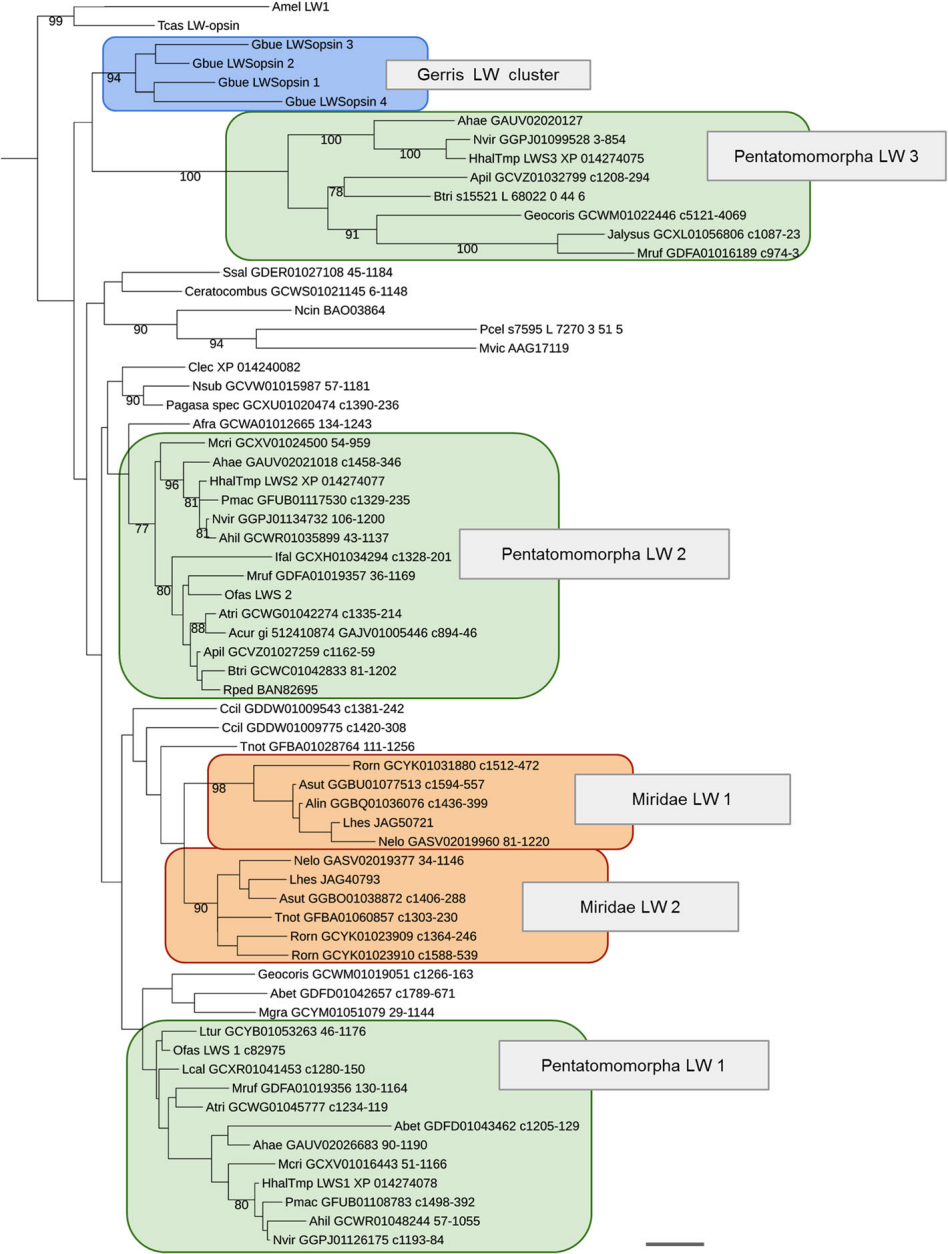
Heteropteran long wave-sensitive opsin gene tree. Scale bar corresponds to 0.1 substitutions per amino acid site. Species abbreviations: Ahae ~ *Acanthosoma haemorrhoidale*, Ahil ~ *Acrosternum hilare*, Alin ~ *Adelphocoris lineolatus*, Acur ~ *Anoplocnemis curvipes*, Asut ~ *Adelphocoris suturalis*, Apil ~ *Alydus pilosulus*, Atri ~ *Anasa tristis*, Aaes ~ *Aphelocheirus aestivalis*, Afra ~ *Aphelonotus fraterculus*, Amel ~ *Apis mellifera*, Abet ~ *Aradus betulae*, Btri ~ *Boisea trivittata*, Clec ~ *Cimex lectularius*, Ccil ~ *Corythucha ciliata*, Gbue ~ *Gerris buenoi*, Hhal ~ *Halyomorpha halys*, Ifal ~ *Ischnodemus falicus*, Lcal ~ *Largus californicus*, Ltur ~ *Lygaeus turcicus*, Lhes ~ *Lygus hesperus*, Mcri ~ *Megacopta cribraria*, Mruf ~ *Metatropis rufescens*, Mgra ~ *Mezira granulata*, Mvic ~ *Megoura viciae*, Nsub ~ *Nabicula subcoleoptrata*, Ncin ~ *Nephotettix cincticeps*, Nvir ~ *Nezara viridula*, Nelo ~ *Notostira elongata*, Ofas ~ *Oncopeltus fasciatus*, Pcel ~ *Pachypsylla celtidismamma*, Pmac ~ *Podisus maculiventris*, Rped ~ *Riptortus pedestris*, Rorn ~ *Reuteroscopus ornatus*, Ssal ~ *Saldula saltatoria*, Tnot ~ *Tupiocoris notatus*, Tcas ~ *Tribolium castaneum*.

In the *H. halys* genome, we found three tandemly duplicated LW-sensitive opsin homologs and a singleton UV-opsin homolog, but no ortholog of the B opsin subfamily (Fig. 3 and Additional file 1: Figure S10). The presence of a single UV-opsin homolog and the lack of B opsins is consistent with the loss of B opsins in the earliest Heteroptera and the broad conservation of UV opsins in this clade. To clarify the relationships between the different LW opsins of water strider, the milkweed bug and *H. halys*, we compiled an alignment of 76 heteropteran LW opsin sequences available from the NCBI TSA division for gene tree reconstruction and analysis (see Additional file 5). This effort revealed that the three LW opsins of *H. halys* and the two LW opsins of the milkweed bug are members of three LW subclades, which are ancestral for pentatomorph Hemiptera with the likely exclusion of the Aradidae, their earliest offshoot (Fig. 4). In addition to robust branch support for each of the three LW opsin subclades, this conclusion was supported by three additional species in which homologs for all three LW opsin subclades were detected: *Acanthosoma haemorrhoidale* (Acanthosomatidae), *Metatropis rufescens* (Berytidae) and *Nezara viridula* (Pentatomidae). Combined, these findings date the expansion of the *H. halys* LW opsin gene cluster to the early evolution of the Pentatomorpha. These LW opsin gene clusters are therefore referred to as the “Pentatomomorpha LW” clades 1-3.

The hemipteran LW opsin gene tree further revealed that the four-member water strider LW-opsin cluster is of independent origin (Fig. 4). In addition to these previously reported hemipteran LW opsin expansions, the LW opsin gene tree unraveled another independent LW opsin expansion in plant bugs (Miridae) (Fig. 4). In the framework of these discoveries, the *H. halys* LW opsin subfamily expansion ranks as one of many examples in the unexpectedly dynamic diversification of LW-opsins in the Heteroptera.

Previous analyses detected candidate tuning substitutions in the protein sequences of two of the four water strider LW opsins, potentially explaining the physiological evidence for blue sensitivity in water striders despite the absence of the B-opsin gene family [12]. Interestingly, all *H. halys* and *O. fasciatus* LW opsin paralogs are characterized by the ancestral green-sensitivity associated residues at the sites of strongest comparative tuning substitution evidence (see Additional file 6). Due to the lack of physiological data on the spectral sensitivities of photoreceptors in the Pentatomomorpha, it is at this point difficult to speculate about the functional corollaries of the LW opsin gene family expansion in this clade. One attractive possibility, however, is the differential deployment of LW opsins in the ocelli and lateral compound eyes, given the conservation of the ocelli in the Pentatomomorpha in contrast to water striders and most Miridae.

Three ancient opsin subfamilies that are expressed in non-retinal tissues have been discovered in insects: ciliary (C) opsins [63], arthropsins (A) [64] and the Rh7 opsins [65]. In *H. halys*, we found singleton orthologs of the Rh7 and C-opsin subfamilies (Fig. 3 and Supp. Additional file 1: Figure S10). No sequence evidence of the A-opsin subfamily was detected in *H. halys* (although this subfamily has been found in the water strider and milkweed bug) [12, 26]. A Hemiptera-wide search in the NCBI NR protein database for C-opsins and arthopsin homologs detected three hemipteran species with singleton orthologs of both C-opsins and A-opsin (*Lygus hesperus*: JAG03839, JAG63746; *Bemisia tabaci*: XP_018896152.1, XP_018897455; and *Diuraphis noxia*: XP_015365906.1, XP_015372008.1). Moreover, all three non-retinal opsins, including Rh7 opsin, have been found in the water strider genome [12]. Taken together, these data constitute unambiguous evidence for the presence of all three non-retinal subfamilies in early hemipterans. Further studies will be needed to clarify whether the discrepancies in C-opsin and A-opsin conservation between *H. halys* and *O. fasciatus* are due to genuine gene losses or insufficient genome sequence coverage.

### Cysteine peptidases from the papain C1 family

Cysteine peptidases from the papain C1 family (MEROPS classification, [66]) are typically lysosomal cathepsins involved in intracellular protein degradation, autophagy, and regulators and signaling molecules in various, more specific biological processes [67, 68]. In some insects, cysteine cathepsins also have evolved from lysosomal ancestors to function as digestive enzymes [69, 70]. Cucujiformia beetles adapted cysteine cathepsins as digestive enzymes to enable survival on seeds containing serine peptidase inhibitors [71, 72]. For example, digestive cysteine cathepsins in *T. castaneum* larvae became important components of adaptive responses in overcoming the effect of cereal protease inhibitors [73]. In *T. castaneum* and the related tenebrionid, *Tenebrio molitor*, large expansions of genes encoding cysteine cathepsins were driven not only by protection against inhibitors, but also by more efficient digestion of complex proteins in grains [74–76].

For insects from the family Pentatomidae, evidence suggests that cysteine cathepsins also participate as digestive enzymes in intraoral digestion. There were early reports of cathepsin B activity in the posterior midgut of the brown stink bug, *Euschistus euschistoides* [77], and later, cathepsin B and L activities were found in the digestive tract of pistachio green stink bug, *Brachynema germari* [78]. Digestive cysteine peptidases also have been described in the midgut of the two-spotted stink bug, *Perillus bioculatus* [79]; spined soldier bug, *Podisus maculiventris* [80]; shield bug, *Apodiphus amygdali* [81] and the southern green stink bug, *N. viridula* [82]. In fact, finding digestive cysteine peptidases in beneficial predatory bugs warranted caution in the development of transgenic plants expressing cysteine peptidase inhibitors that target plant pests [80].

**Fig. 5:**
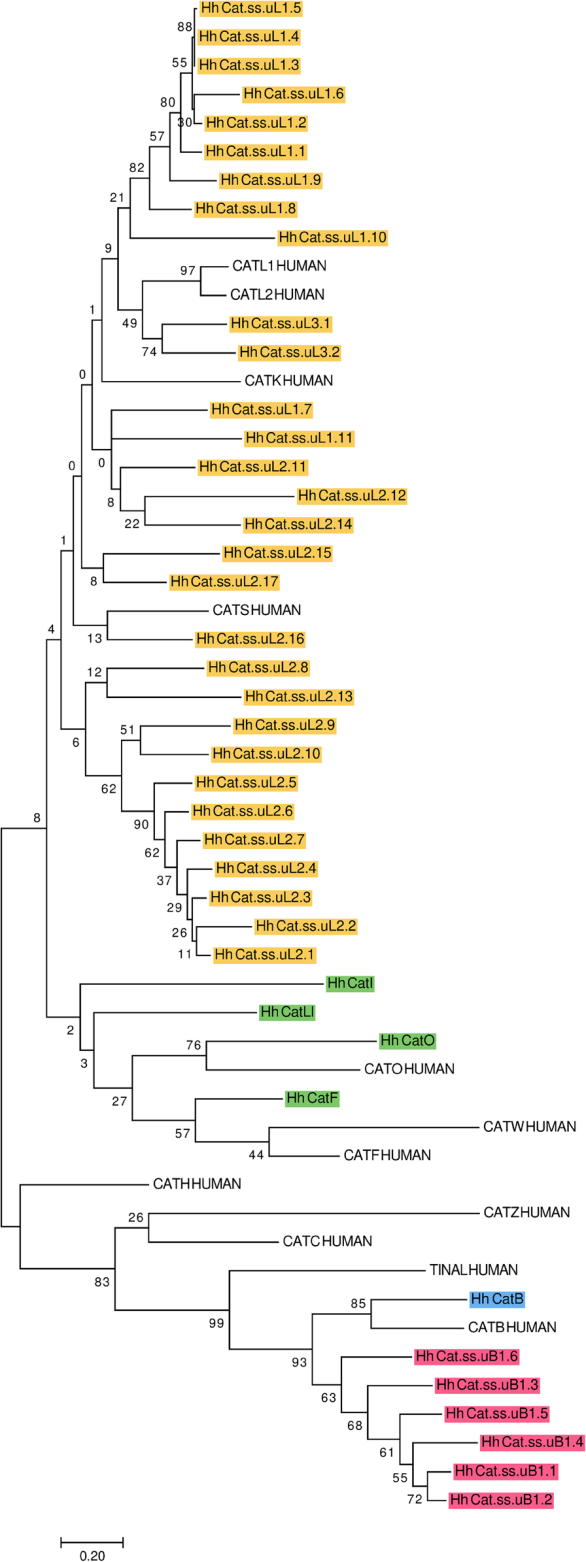
Phylogenetic tree of cysteine cathepsins. An analysis of predicted proteins from cysteine cathepsin genes annotated in the draft genome of *H. halys* was performed using MEGA7. The tree with the highest log likelihood is shown (-9596.54). The percentage of trees in which the associated taxa clustered together is indicated beside the branches. The tree is drawn to scale, with branch lengths measured in the number of substitutions per site. Cysteine cathepsins annotated in *H. halys* include those that are conserved in the cathepsin L-like subfamily (Hh CatF, Hh CatO, Hh CatI, Hh CatLl) in green and species-specific (Hh Cat ss.uLX.x) in yellow; cathepsin B ortholog (Hh CatB) in blue and species-specific cathepsin B-like (Hh Cat ss.uLX.x) in pink; and human cathepsins, which are marked according to UniProt IDs: L (CATL1_HUMAN, P07711), V (CATL2_HUMAN, O60911), F (CATF_HUMAN, Q9UBX1), O (CATO_HUMAN, P43234), H (CATH_HUMAN, P09668), K (CATK_HUMAN, P43235), S (CATS_HUMAN, P25774), W (CATW_HUMAN, P56202), Z (CATZ_HUMAN, Q9UBR2), B (CATB_HUMAN, P07858), C (CATC_HUMAN, P53634) and TINAL-like protein (TINAL_HUMAN, Q9GZM7). Correspondences between leaf node identifiers and NCBI protein sequences are indicated in Additional file 1: Table S14.

In *H. halys*, we found 41 genes and gene fragments encoding cysteine cathepsins of the C1 family (Fig. 5). Thirty-four genes belong to the cathepsin L-like subfamily (yellow and green), and seven are from the cathepsin B-like subfamily (pink and blue, [83]). All cathepsins fit two types of peptidase gene categories: 1) those encoding conserved cathepsins, orthologous to mammalian or most insect cathepsins and 2) species-specific cathepsins that lack orthologs in other insects described thus far and appear unique to *H. halys*, perhaps also to the genus *Halyomorpha* or the family Pentatomidae. Conserved cathepsins include cathepsin L-like subfamily genes (shaded green; including orthologs of mammalian cathepsins F (Hh CatF) and O (Hh CatO); and orthologs of insect cathepsins I (Hh CatI) and Ll (26-29kD-proteinase, Hh CatL1), as well as cathepsin B-like subfamily gene (shaded blue; including an ortholog of mammalian cathepsin B (Hh CatB)).

The most numerous group is the species-specific category, containing species-specific cathepsin L-like genes (shaded yellow) with 30 members clustered in three phylogenetic clades derived from the cathepsin L subfamily enzymes (Hh Cat.ss.uLx.x). These species-specific genes are localized in the draft genome assembly either as separate genes or as tandem arrays of up to seven copies. Species-specific cathepsin B-like peptidases (shaded pink) are represented by one phylogenetic clade of six genes (Hh Cat.ss.uBx.x). We propose that these species-specific genes in *H. halys* encode digestive peptidases to enable specific functions, such as expanded dietary choices. This hypothesis is supported by similar species-specific clades of cysteine peptidases in *T. castaneum* [74, 75], *T. molitor* [74, 84] and *Leptinotarsa decemlineata* [85, 86], which were demonstrated to be involved in digestion.

Surprisingly, we did not find complete gene sequences of one of the major conserved cathepsins–“true” cathepsin L (ortholog of human CatL1 or L2), as well as a conserved and presumably catalytically inactive TINAL-like protein from the cathepsin B-like group [87]. These sequences have been annotated in most of the insect genome assemblies prepared to date [12, 74, 86] and may eventually be found in an improved *H. halys* genome assembly. If in fact they are not encoded in the genome, this divergence would have significant biological and evolutionary consequences.

### Salivary effector genes

*H. halys*, like other hemipteran species, injects saliva into plants using highly evolved, needle-like and flexible mouthparts (i.e., stylets) [88]. Hemipteran saliva contains effector proteins, which manipulate the structure and function of host cells so as to promote insect feeding and survival [89]. Salivary effectors not only suppress host defenses, which is imperative for successful colonization, but may also perform extra-oral digestion following their secretion into the plant. We identified and annotated 64 genes encoding salivary effector proteins in the *H. halys* genome (Additional file 1: Table S15). Gene expression data for select instances exhibiting differences between nymphal and adult stages (or sexes) is shown in Additional file 1: Table S16. Our analysis into the evolution of these genes yielded negative test statistic (dN-dS) values, suggesting higher synonymous subsitutions than nonsysnonymous (P>0.05) (Additional file 1: Table S16). This lack of evidence for positive selection in *H. halys* effectors is consistent with the mode of selection observed in aphid effectors [90]. Several *H. halys* genes encoded salivary proteins having 1:1 orthologs amongst the best-studied effectors in herbivorous hemipterans (Table 3). For example, *H. halys* has genes for Armet (homolog of “Mesencephalic astrocyte-derived neurotrophic factor” in pea aphid, *A. pisum* [91]), Ejaculatory bulb-specific protein (a homolog of Mp10 in green peach aphid, *Myzus persicae* [92, 93]), Angiotensin-converting enzyme (homologous to Ace2 in *A. pisum* [94]), and Mucin (effector homolog in brown planthopper, *N. lugens* [95, 96]). There is evidence for all these proteins to be secreted by herbivorous hemipterans into plant hosts through saliva. Though specific functions for these proteins are not yet known, RNAi targeting their gene expression has shown severe negative impacts on insect survival, thus validating the critical role played by salivary effectors in hemipteran growth and colonization [91–96]. Herbivorous hemipterans inject Ca^2+^ binding proteins into plants to suppress the activation of defense cascades by Ca^2+^, a secondary messenger for signal transduction in plants [90]. Accordingly, we annotated three genes coding for Ca^2+^ binding proteins (calreticulin, sarcalumenin and endoplasmin) as salivary effector genes. Further, we annotated three genes for disulfide isomerases, thought to aid in the gelling nature of sheath saliva by catalyzing the formation of disulfide bridges in proteins [90]. We identified four genes encoding antioxidant enzymes known to degrade reactive oxygen species, which are part of the plant’s initial defense response [90]. We also identified four genes for cysteine proteinase-like cathepsins, thought to remove harmful plant proteases and protease-inhibitors, and to perform extra-oral digestion. In addition, we annotated other genes for peptidases, lipases and glucosidases that may help *H. halys* overcome a plant’s physical and chemical defenses and/or perform extra-oral digestion. The discovery of salivary effector genes in the *H. halys* genome is significant because these genes were recently found to play a key role in generalist herbivory behavior [110], which has likely aided the rapid spread and successful establishment of *H. halys* across North America.

**Table 3:** *H. halys* salivary effectors with homologs previously implicated in mediating herbivorous hemipterans’ interaction with plant hosts

Salivary effector	Gene symbol	Protein Reference	Pea aphid homolog	BLAST E-value	Comment
Armet/mesencephalic astrocyte -derived neurotrophic factor	LOC106681713	XP_014277663.1	ACYPI008001	1.6e-45	Found in pea aphid saliva and aphid-fed plants. RNAi targeting *Armet* expression disrupted aphid feeding behavior leading to reduced life span [91].
Mp10/ejaculatory bulb-specific protein	LOC106681352	XP_014277113.1	ACYPI000097	1.6e-26	Mp10 from green peach aphid induced chlorosis, localized cell death *in planta*, and triggered plant defenses [92, 93].
Angiotensin-converting enzyme, Ace2	LOC106681465	XP_014277274.1	ACYPI007204	0	Ace, a M2 metalloprotease, potentially degrade short signaling peptides capable of inducing plant defense. RNAi simultaneously targeting *Ace1* and *Ace2* expression in pea aphid caused significant mortality [90, 94].
Mucin-like	LOC106684151	XP_014281554.1	ACYPI001019	1.6e-41	Found in both gelling and watery saliva of rice brown planthopper. RNAi targeting its expression disrupted the salivary sheath formation leading to disordered developmental duration and poor performance [95, 96].
Calreticulin	LOC106681650	XP_014277576.1	ACYPI002622	0	Calcium binding proteins found in saliva of southern green stink bug and various aphid species, putatively involved in suppressing the activation of defense cascades [82, 90, 97].
Sarcalumenin	LOC106681164	XP_014276825.1	ACYPI001446	0	
Endoplasmin	LOC106681661	XP_014277591.1	ACYPI009915	0	
Digestive cysteine proteinase 1	LOC106685481	XP_014283673.1	ACYPI003954	5.4e-170	Cysteine proteinase-like cathepsins have been found in saliva of southern green stink bug [82], these enzymes potentially degrade plant defense peptides and/or perform extra-oral digestion of dietary proteins.
Cathepsin B	LOC106690036	XP_014290885.1	ACYPI000003	3.2e-139	
Cysteine proteinase	LOC106682432	XP_014278766.1	ACYPI000376	1.7e-123	
Cathepsin L1	LOC106682597	XP_014279027.1	ACYPI006974	3e-114	
Disulfide isomerase	LOC106677432	XP_014270846.1	ACYPI005594	0	Found in saliva of southern green stink bug and various aphid species, potentially aid in gelling nature of sheath saliva by catalyzing the formation of disulfide bridges in proteins [90, 98].
	LOC106678635	XP_014272751.1	ACYPI009755	0	
	LOC106686982	XP_014286089.1	ACYPI008926	1e-172	
Superoxide dismutase	LOC106681155	XP_014276814.1	ACYPI003921	2.56e-58	Antioxidant enzymes have been found in saliva of southern green stink bug and various aphid species [82, 90, 99–102], and supposedly degrade reactive oxygen species, plant’s initial defense response.
Peroxidase-like isoform X1	LOC106692485	XP_014293941.1	ACYPI000817	1.2e-108	
Peroxiredoxin-2	LOC106681766	XP_014277748.1	ACYPI003960	9.2e-138	
Selenoprotein-like	LOC106691878	XP_014293266.1	ACYPI003278	3.7e-69	
Neutral alpha-glucosidase	LOC106679031	XP_014273428.1	ACYPI009457	0	Found in saliva of plant tarnished bug, glassy-winged sharpshooter, and potato aphid, potentially break down complex carbohydrates such as cellulose in plant cell wall [103–105].
Trehalase	LOC106681721	XP_014277675.1	ACYPI002298	0	Found in saliva of various aphid species; putatively suppresses the activation of plant defenses by disrupting signal transduction [90, 97, 98, 106].
Aminopeptidase N	LOC106686134	XP_014284771.1	ACYPI002258	0	Found in saliva of southern green stink bug, tarnished plant bug, and pea aphid; potentially destroy plant defense and signaling peptides [82, 90, 105].
Carboxypeptidase E	LOC106686022	XP_014284587.1	ACYPI001238	0	Found in saliva of various hemipteran species [90, 107], potentially degrade plant defense peptides and/or perform extra-oral digestion of dietary proteins.
Pancreatic triacylglycerol lipase	LOC106692440	XP_014293875.1	ACYPI009369	3.0e-60	Lipases have been found in saliva of various hemipteran species [99, 103, 107–109], potentially interfere plant defense by binding to lipids and/or perform extra-oral digestion of dietary proteins
Pancreatic lipase-related protein	LOC106679755	XP_014274569.1	ACYPI003852	0	
Apolipophorins	LOC106679717	XP_014274514.1	ACYPI004198	1.1e-76	Found in saliva of various aphid species [90, 99, 108], potentially interfere plant defense signaling.
Glycosyltransferase	LOC106688290	XP_014288163.1	ACYPI002729	0	Several glycosyltransferases have been found in saliva of southern green stink bug [82].
Protein yellow	LOC106690949	XP_014292045.1	ACYPI000479	1e-168	Found in two cereal aphid species, it potentially targets the phenoloxidase-based defense in plants [106].
	LOC106680598	XP_014275905.1	ACYPI001857	9e-112	Several glycosyltransferases have been found in saliva of southern green stink bug [82].

### Insect Immunity

Genes for the Toll signaling cascade, involved in both development and innate immunity, were annotated. Several sequences homologous to known Toll-pathway transmembrane receptors were encountered: *toll*, *toll-interacting isoform X1*, *toll family protein 10*, *toll-6* and *toll-7* were present on four different scaffolds. Single copies of *spaetzle*, myeloid differentiation primary response protein (MyD88), *pelle kinase*, *dorsal*, *tube*, *cactus*, *cactin*, *traf*, *pellino*, *persephone* and various serine proteases (serpins) were also confirmed by manual annotation and BLAST alignments. However, the *dif* (encoding dorsal related immunity factor) gene was not found. In *Drosophila*, dif is a second cactus-bound NfK-B transcription factor (in addition to dorsal) that functions primarily in the immune response rather than in playing a developmental role. Unlike dorsal, dif can be activated in both a *Toll-*dependent and independent manner depending on the challenge [111]. The absence may imply a more important role for *dorsal* in immunity in *Hemiptera*, or possibly reduced specificity and complexity of response to certain pathogens.

The JAK/STAT pathway in *Drosophila* is also involved in both development and immunity. It is hypothesized that induction of the JAK/STAT pathway leads to overproliferation of hemocytes and an upregulation of thiolester-containing proteins (TEPs), as well as triggering of the antiviral response [112]. The *H. halys* genome has homologs of all core JAK/STAT genes, including genes encoding the cytokine receptor Domeless, JAK tyrosine kinase Hopscotch, and the Signal Transducer and Activator of Transcription (Stat) transcription factor, which was found on NW_014466899.1:1005475-1044464, with highest homology to the Stat 5B isoform X1 from *Bombus terrestris* (XP_003401031.1). Two thiolester-containing proteins (TEPs) were located on NW_014467684.1. A putative uroporphyrinogen decarboxylase (*upd*, or “unpaired”), considered a key ligand in *Drosophila* JAK/STAT induction, was located on NW_014466467.1. Interestingly, this ligand is missing in other insects, such as *A. mellifera* [113]. In contrast, *Drosophila* encodes three upd-like ligands; however, their sequences are highly divergent, and this degree of divergence may account for why orthologs have not been identified in other insects. In *Drosophila*, upd-3 is largely responsible for the activation of the Jak-STAT pathway in the context of an immune response. Additionally, in both *Ae. aegypti* and *Drosophila*, an alternate activating ligand, vago, initates a Jak-STAT response to virus infection [114, 115]. *Vago* was not found in the *H. halys* genome, and this absence may imply an alternate means of responding to viral challenge.

The IMD and JNK signaling pathways were complete with the notable exception of the immune deficiency (IMD) death domain protein itself, which initiates the IMD signaling pathway after peptidoglycan recognition protein (PGRP) attaches to the cell membrane. The lack of IMD is not necessarily unusual, as it has also not been found in the pea aphid, *A. pisum*; the body louse, *Pediculus humanus corporis*; or the deer tick, *Ixodes scapularis*. Indeed, recent evidence suggests IMD is absent among hempiterans [116, 117], although its absence indicates an as-yet-uncharacterized means for transducing a PGRP-initiated signal in response to bacterial challenge.

The JNK pathway role in antimicrobial peptide gene expression and cellular immune responses is very well described in other insects [118, 119]. *H. halys* contained all requisite genes for the signaling pathway, including *tab (1), tak (3)*, *hep (1)*, *basket (2)*, *jra (2)* and *kayak (1).* Furthermore, the signaling genes *kenny, ird5* and *relish* were also found, but not *iap2*. The procaspase precursors to apoptosis, death domain protein Dredd, as well as two homologues of the aspartate-specific cysteine proteases CASP1 were annotated, indicating that the signaling pathway to apoptosis is intact, even in the absence of IMD. The absence of *fadd* is notable, as this may be unique to the *Drosophila* pathway due to its role in making flies susceptible to Gram-negative bacterial infection [120].

Eiger (NW_014467110.1:500566-503446) has been proposed as an IMD-independent alternative inducer for activation of the protein kinase TAK (3 putative versions found on scaffolds NW_014466634.1, NW_014466754.1 and NW_014466862.1), which then triggers the the JNK pathway [121]. A BLAST search for the inducer Eiger protein from a consensus UniProt set of arthropod sequences found a homolog in the pea aphid, *A. pisum*. This is consistent with the observation that Eiger serves as an IMD-like inducer for TAK in *H. halys*, similar to the pea aphid, whose genome also lacks IMD notwithstanding an intact JNK signaling pathway [122].

In addition to immune signaling pathways, numerous other genes likely involved in insect immunity were identified, including PGRPs, Gram-negative binding proteins, lectins, antimicrobial peptides, RNA interference pathway components and a variety of such miscellaneous immune-related genes as putative prophenoloxidases and nitric oxide synthases (see Additional file 1).

### Xenobiotic detoxification genes

Detoxification of xenobiotic compounds is an imperative cellular function that protects the organism from harmful compounds it may encounter. *H. halys* has a broad host range and wide geographic distribution, increasing the likelihood of encountering xenobiotic substances in the form of plant defensive compounds and insecticides. Glutathione S-transferases (GSTs), carboxylesterases (COEs) and cytochrome P450s (CYPs) are three well-documented gene families associated with xenobiotic detoxification in insects. Additionally, they have all been associated with increased insecticide tolerance and/or insecticide resistance in other insects through various mechanisms. Understanding *H. halys*’ xenobiotic detoxification gene repertoire is vital for successful pest control and to combat insecticide resistance that may arise in the future.

#### Glutathione S-transferases (GSTs)

Glutathione S-transferases (GSTs) compose a large gene family associated with xenobiotic detoxification. Microsomal GST enzymes are typically trimeric and membrane bound, while cytosolic GSTs are typically dimeric and unbound [123]. Based on current knowledge, an insect can possess at most six cytosolic GSTs subclasses: Delta, Epsilon, Sigma, Omega, Theta and Zeta [124, 125]. Of the microsomal and six cytosolic subclasses, only the cytosolic Delta and Epsilon subclasses have been associated with insecticide resistance [126]. The Delta subclass is present across Insecta, while the Epsilon subclass is only present in the Holometabola [125].

**Fig. 6:**
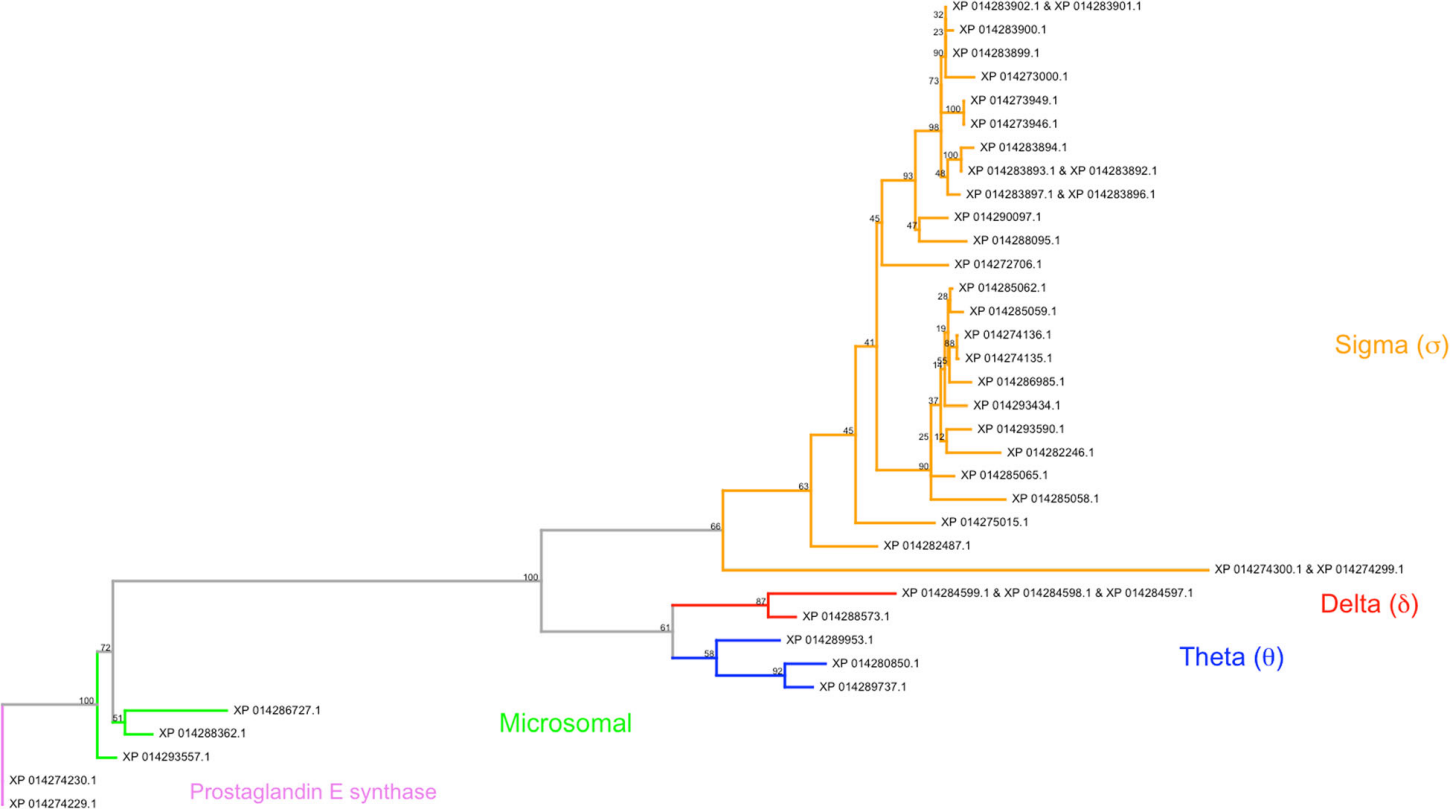
Phylogenetic tree of *H. halys* glutathione S-transferase (GST)-associated proteins. Contains, microsomal (green), theta (blue), delta (red) and sigma (orange) clades. Two prostaglandin E isoforms (purple) are used as outgroups to all GSTs. Uncolored leaves could not be assigned to family based on annotation. Bootstrap support (100 replicates) is indicated on nodes.

Thirty unique loci in *H. halys* transcribe 41 unique transcripts, which in turn translate 35 unique GST protein sequences (i.e., some isoforms encode identical protein sequences; see Additional file 1: Table S17). *H. halys*’ genome encodes three Theta, two Delta and 25 distinct Sigma GST proteins (Fig. 6). Four distinct clades corresponding to GST class and sub-class were observed: Theta, Delta, Sigma, and microsomal. The majority of protein sequences were placed in the Sigma clade. Two prostaglandin E synthase isoforms were outgroups to all GSTs, and a long branch with 100% bootstrap support separates the three microsomal GSTs from all cytosolic variants. Expression data for all GST transcripts, obtained using data from prior transcriptome studies (see Additional file 1: Table S1), are presented in Additional file 1: Table S17.

The 30 glutathione S-transferase genomic loci present in the *H. halys* genome harbor 21 Sigma GST genes. Sigma GSTs detoxify reactive oxygen species in active muscle tissues and provide a structural role in less active muscle tissues [127]. Many species have only one Sigma GST gene; however, more than one Sigma GST gene have been reported in *A. pisum* (6), *A. mellifera* (4), *N. vitripennis* (8), *T. castaneum* (6) and *B. mori* (2), and may result in new endogenous functions [128]. *H. halys*’ large complement of Sigma GSTs could correspond with structural roles and/or detoxification of reactive oxygen species in muscle tissue or other novel endogenous roles.

High counts of Delta GSTs have been reported in *An. gambiae* (15) and *D. melanogaster* (11) [129, 130]. *H. halys* appears to possess only two Delta GST genes. High expression levels of Delta GSTs are a mechanism for conferring insecticide resistance, obtained by either upregulation of expression, or gene duplication. Insecticide resistance in *H. halys* has not yet been reported. Sparks et al. (2014) [131] noted increases of glutathione S-transferase transcript expression levels of adult *H. halys* in response to septic puncture: adult females exhibited a 9.8-fold change and adult males a 6.1-fold change. This up-regulation could be in response to foreign substances introduced during septic puncture and/or to help process a potential increase of metabolic products resulting from an immune response. The ability of *H. halys* to quickly alter expression levels of Delta GSTs, as seen after septic puncture, suggests the insect may utilize this mechanism in response to insecticide exposure.

#### Carboxylesterases

The carboxylesterase (COE) family is typically divided into three clades [132–134]. The neurodevelopment clade contains generally non-catalytic neuroligin, glioactin and neurotactin proteins, as well as catalytic acetylcholinesterases. The hormone/ semiochemical processing clade includes secreted β-esterases, integument esterases and juvenile hormone esterases. Several mechanisms of insecticide resistance via carboxylesterases have been reported—most notably, point mutations of acetylcholinesterase, which prevent insecticide inhibition, and duplication of β-esterase genes to produce high levels of insecticide-sequestering enzymes [132]. The third clade, related to dietary and detoxification functions, contains α-esterases and has not been associated with insecticide resistance.

**Fig. 7:**
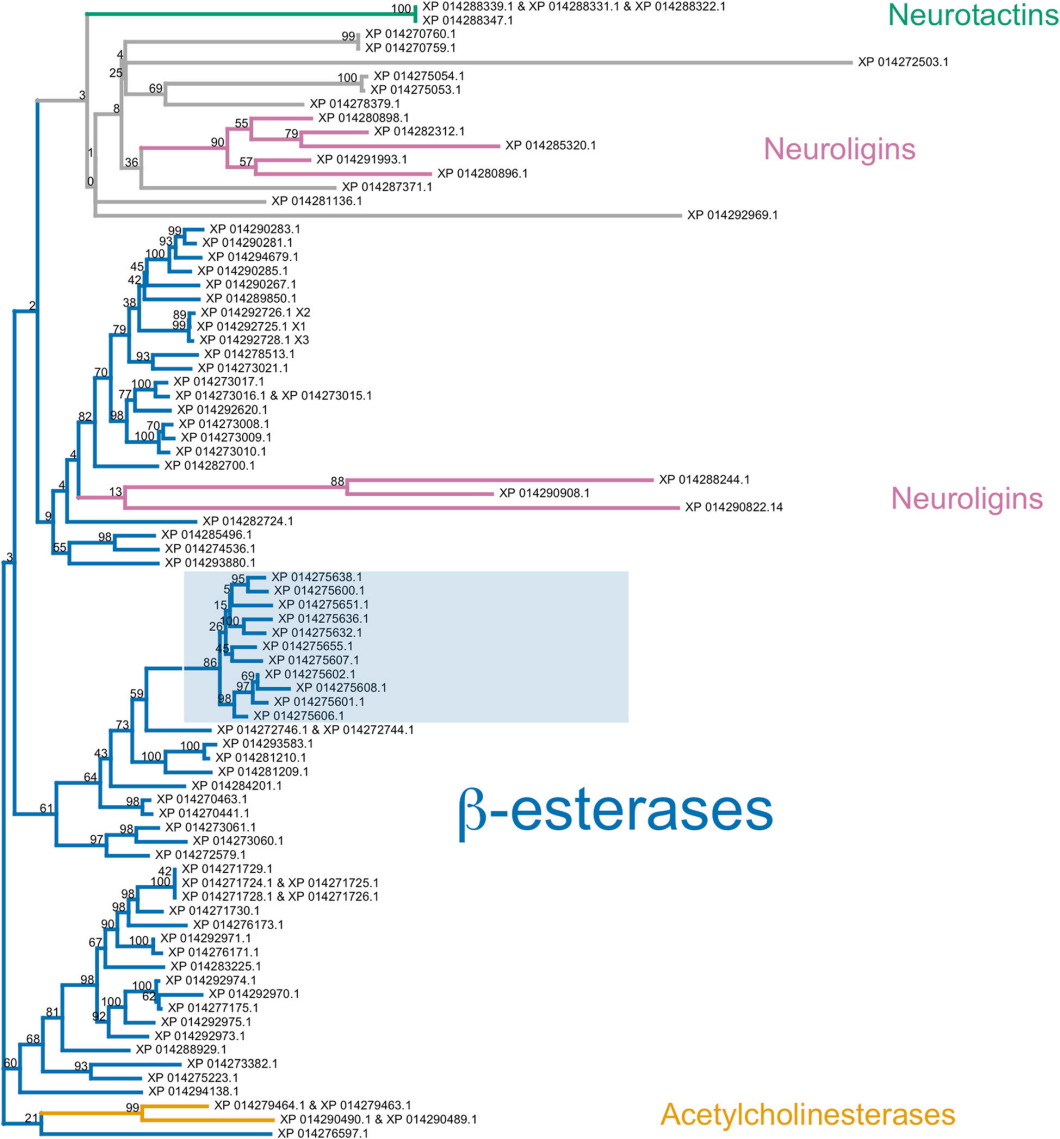
Phylogenetic tree of *H. halys* carboxylesterase-associated proteins. Contains β-esterases (blue), neuroligins (purple), acetylcholinesterases (orange) and neurotactins (green) groups. Uncolored leaves could not be assigned to family based on annotation. Shaded box represents the monophyletic grouping of the eleven indicated loci on Scaffold NW_014466677.1. Bootstrap support (100 replicates) is indicated on nodes.

In total, there are 75 genomic loci from which 90 distinct transcripts arise and which translate to 82 unique COE protein sequences. Of these, 59 are β-esterase genes, which produce 68 unique transcripts in total. A bootstrapped maximum likelihood phylogenetic tree of all unique translation products is provided in Fig. 7. The majority of predicted protein sequences are β-esterases and resolve to three clades with low bootstrap support and short branch lengths, indicating a high level of similarity. The annotations of these sequences vary; most are labeled either as E4-like, FE4-like or venom COE-6-like (all of which were labeled as β-esterases). Several uncharacterized proteins and one annotated as para-nitrobenzyl esterase-like were also placed among the β-esterases. A group of two pairs of acetylcholinesterase isoforms, corresponding to two separate genomic loci, and a group of three neuroligins were placed among the β-esterases. A separate clade contains neurotactins, neuroligins, COE 4, COE 5A and several uncharacterized or possibly misannotated sequences. This clade generally has low base node bootstrap support and long branch lengths.

The *H. halys* genome contains multiple scaffolds with β-esterase gene duplications in close proximity: *H. halys* scaffolds NW_014466677.1, NW_014469008.1, NW_014466575.1, NW_014467841.1 and NW_014466532.1 contain 11, 6, 6, 4 and 2 β-esterase genes, respectively. Within each of these scaffolds, inferred protein sequences are typically highly similar and cluster together in the phylogeny. For example, all eleven β-esterase genes on NW_014466677.1 are organized in a tandem array (Additional file 1: Figure S11) and whose translation products constitute a monophyletic clade in Fig. 7 (see shaded box). β-esterase gene duplication exists in many insects, most notably the aphid *Myzus persicae* [135, 136], numerous *Drosophila* species [137–139] and the mosquito *Culex pipiens* [140]. Gene duplication allows for new enzymatic functions to evolve while allowing the parent function to remain [141, 142]. *Drosophila* species vary greatly in esterase gene duplication, some of which have developed new functions [137–139]. The mixture of *H. halys* β-esterase annotations demonstrates that these similar protein sequences differ enough to affect annotation and suggests possible gain of novel functions. For example, of the eleven β-esterase genes located on scaffold NW_0144666772 (Fig. 7, shaded box), eight are annotated as venom COE-6-like, one as E4-like, one as an uncharacterized protein and one as para-nitrobenzyl esterase-like. Gene duplication can also increase protein expression. High levels of β-esterase expression via tandem duplication has been shown to confer insecticide resistance in both *M. persicae* [143] and *C. pipiens* [144]. Given *H. halys’* broad agricultural impact and exposure to insecticides, its tandem array β-esterase duplications could serve as a means for the emergence of insecticide resistance.

The paraphyletic placement of neuroligins, some of which are derived within β-esterases, as well as acetylcholinesterases derived within β-esterases, may be caused by the small size of the phylogenetic tree and the sharing of protein domains. The branch lengths of neuroligins and acetylcholinesterase in Fig. 7 demonstrate that they are quite different from β-esterases and are potentially misplaced in our phylogenetic tree, likely due to a long branch-attraction artifact. Sparks et al. (2017) [145] combined these data with protein sequences from the harlequin bug, *Murgantia histronica*, and the resulting phylogeny (see Figure S3 of Sparks et al. (2017) [145]) places the neurodevelopment-associated carboxylesterases within their own monophyletic clade, an outgroup to the β-esterase clade. The additional information utilized by this multi-species analysis suggests it may convey a more accurate representation of the overall relationships among COEs in *H. halys* than does the single-species phylogeny, underscoring the importance of sequencing genomes from additional pentatomid taxa to enable comparative genomics studies (and thus, more informative phylogenetic gene family analyses) in the future.

#### Cytochrome P450s

Cytochrome P450s (CYPs) have a two-fold role in gene-environment interactions, participating both in detoxification of xenobiotic compounds and in host biosynthetic pathways. P450-mediated biosynthesis of critical endogenous molecules affecting molting, hormone/pheromone synthesis and turnover, and cuticular hydrocarbon waterproofing processes can be targeted by pesticides. In response, insects have evolved modified P450s to detoxify exogenous chemicals like pesticides, leading to resistance. These two classes of P450s are easily observed in phylogenetic trees. Highly conserved one-to-one orthologs between insect species are parts of pathways to make essential biomolecules like ecdysone (Halloween genes: CYP302, CYP306, CYP307, CYP314 and CYP315 [146];), juvenile hormone (CYP15 [147];), as well as fatty-acid-derived alkanes and alkenes for exoskeleton coating (CYP4G [148, 149];).

Resistance has been associated with numerous cytochrome P450s, often members of “gene blooms,” which are large expansions of P450s in tandem duplication arrays on chromosomes. These are not highly conserved or even limited to one CYP clan. Almost any P450 family can become adapted to detoxify a pesticide [150–152]. Resistance may not only be due to pesticide inactivation, but it may be caused by blocking pesticide entry via thickening of the cuticular hydrocarbon barrier [153]. On the biocontrol side, entomopathogenic fungi kill insects by using P450s like CYP52X1 to degrade and penetrate the hydrocarbon coating on insects [154].

**Fig. 8:**
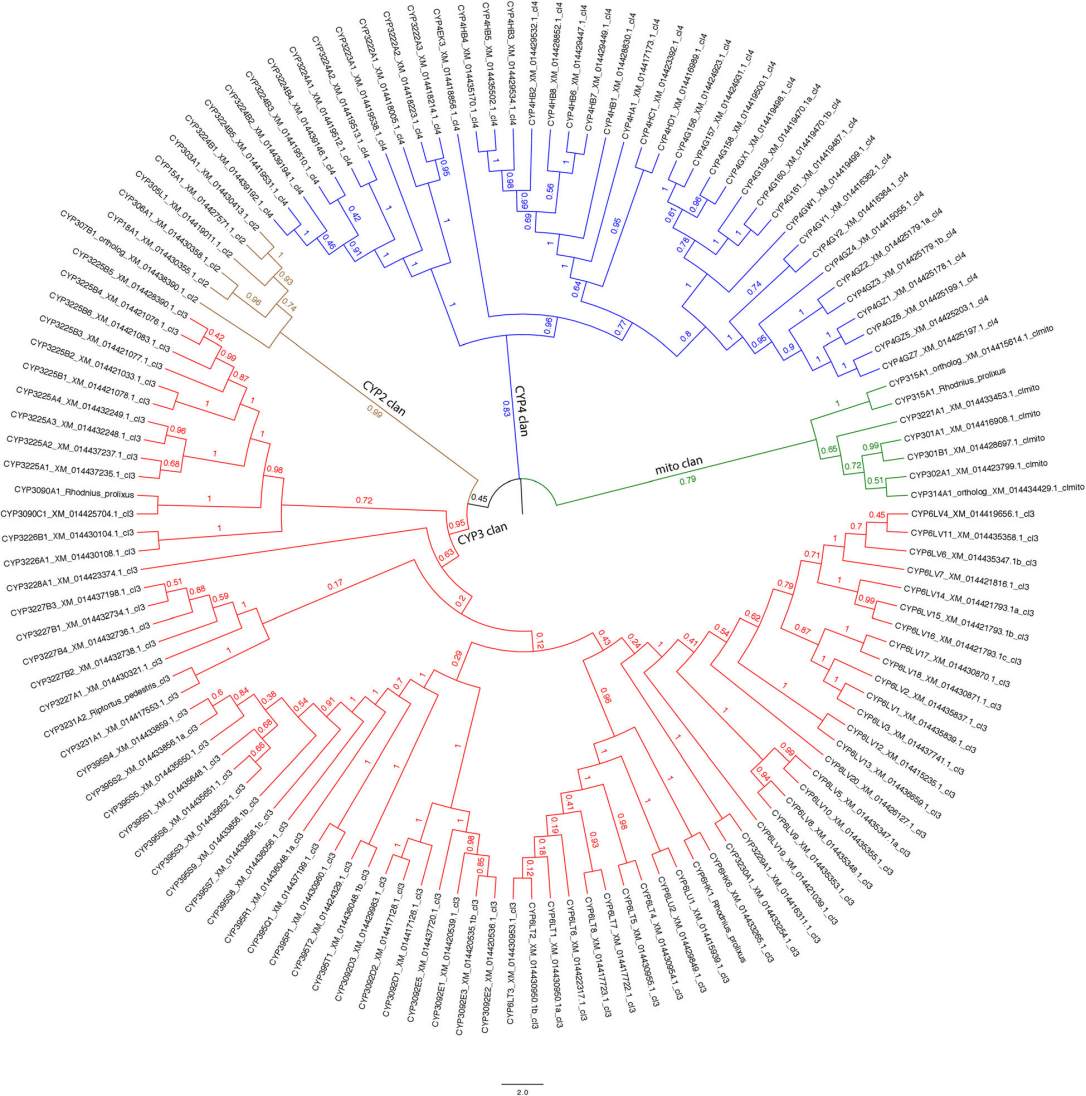
Phylogenetic tree of cytochrome P450s. MEGA X was used to phylogenetically analyze relatedness among cytochrome P450s in *H. halys*. The maximum likelihood tree (log likelihood = -87136.93) is shown. Branch lengths in this cladogram correspond to substitutions per site. The four cytochrome P450 clans are depicted as follows: brown ~ CYP2, red ~ CYP3, blue ~ CYP4 and green ~ mito

The 141 *H. halys* P450s sorted into the four known P450 clans: CYP2 (6 sequences), CYP3 (84 sequences), CYP4 (45 sequences) and mito (6 sequences). A maximum likelihood tree was constructed from 126 full or nearly full-length sequences, excluding 14 fragments and one pseudogene. Four additional sequences were included to stabilize the positions of single outlier sequences in the tree (see Fig. 8, and Materials and Methods). The CYP2 and mito clans contain all of the halloween genes for ecdysone synthesis CYP302A1, CYP306A1, CYP307B1, CYP314A1, CYP315A1 [146] and CYP18 for 20-hydroxy ecdysone turnover [155]. The 4G subfamily has six genes. Specific CYP4G sequences have been shown to make a waterproof hydrocarbon coating for the exoskeleton to prevent dehydration [148, 149, 156]. CYP15A1 in other insects is committed to juvenile hormone synthesis [147, 157]. CYP301A1 is another conserved P450, having a role in cuticle formation in *Drosophila* [158]. Also found in *Drosophila*, the CYP303A1 gene is required for the structure and function of sensory organs [159]. Other P450s such as CYP301B1 and the CYP305 family are conserved among other insects, but the role of these enzymes is not known yet. The large number of CYP3 and CYP4 clan sequences in the *H. halys* genome may be involved in synthesis of specific chemicals such as the stink smell (trans-2-decenal and trans-2-octenal). CYP74 family P450s are involved in generating hexenal in plants by a hydroperoxide cleavage reaction. Although no *H. halys* CYP74 was found, convergent evolution could produce similar products. P450s are known to be involved in pheromone clearance in the antenna [160] and this must occur in stink bugs as well, though the genes have not been identified.

## Conclusions

The collaborative genome sequencing and annotation efforts reported here suggest identities of the genetic determinants underlying the highly invasive, generalist nature of the brown marmorated stink bug. BUSCO and OrthoDB assessments indicate that the *H. halys* genomic resource has a high degree of gene content completeness, and the overall high quality of the assembly is also corroborated by well-assembled Hox and Iro-C gene clusters, in addition to two independent contamination screens (see Additional file 1). At least six lateral gene transfer events from *Wolbachia* and other bacteria into the *H. halys* genome are evident. LINE-type non-LTR retrotransposons are the predominant repetitive element observed in the assembly, accounting for 124 Mb of overall sequence. A Gypsy-type LTR retrotransposon form is heavily under-represented, however, suggesting a significant and recent accumulation of this family of transposable elements (see Additional file 1).

*H. halys*’ highly polyphagous nature might be partially explained by its extensive complement of chemoreceptors, especially its array of gustatory receptors, which through gene copy number and variation in spliced isoforms constitutes one of the largest such repertoires yet observed in insects. The ratio of odorant receptor to odorant binding protein (OBP) genes in this species is approximately 3:1, not unusal for an insect. Most OBP genes are organized into gene clusters, with an atypical instance of an OBP gene being embedded within the intron of an odorant receptor per the draft assembly. Although LWS and UV opsin homologs are observed in *H. halys*, no ortholog of the SWS-B opsin subfamily was detected, consistent with the notion that this subfamily was lost during early heteropteran diversification.

Cysteine cathepsins of the C1 family observed in *H. halys* include 34 genes from the cathepsin L-like subfamily and seven from the cathepsin B-like subfamily—30 and six of these, respectively, seem to represent *H. halys*-specific instances of cysteine peptidases, which may have been involved in the diversification of the insect’s broad dietary selections. The discovery of 64 salivary effector genes—several of which are homologous to known effectors in other herbivorous hemipterans—is significant due to the key role such genes play in the type of generalist herbivory exhibited by this species. Expansion of a cell wall degradation mannosidase, originally incorporated via an LGT event, to nine copies may also contribute to the digestive capabilities of this insect, providing a clear candidate enzyme for future functional assays and demonstrating the need for further species sampling within the Pentatominae and close relatives.

Genes encoding Toll and JAK/STAT pathway components, as well as all elements of the JNK signaling pathway, were observed. All components of the IMD pathway were present with the exception of IMD itself, which initiates this pathway *in vivo*. This apparent lack of the IMD initiator in *H. halys* is consistent with findings made among other hemipteran species. A variety of additional immunity-related genes were also identified in the genome assembly, including peptidoglycan receptor proteins, gram-negative binding proteins, lectins, and the requisite molecular machinery to enable RNAi (see Additional file 1).

The brown marmorated stink bug appears to encode only two Delta-class glutathione S-transferase genes, which have been associated with insecticide resistance development in other taxa—although the genome contains only two copies, it is possible that an up-regulation in gene expression alone, under specific circumstances, could be sufficient to confer a resistant phenotype. Regarding carboxylesterases, 59 β-esterase genes were identified, 29 of which were present in tandem array configurations on five separate scaffolds. In addition, two acetylcholinesterase genes and various neuroligins are present, all pointing to an innate capacity for the development of insecticide resistance. In *H. halys*, 141 cytochrome P450s were observed, sorting into the four known P450 clans: CYP, CYP3, CYP4 and mito. Members of this gene family can confer insecticide resistance, are involved in insect development and very likely also play a role in synthesizing the chemicals responsible for this insect’s characteristic odor.

Analyses presented in Additional file 1 demonstrated that 462 transcription factors are present in the genome. Strong evidence for the presence of orthologs for all nine *D. melanogaster* pair-rule genes was found in *H. halys*. In addition, highly conserved family members, such as *gsb, lozenge*, and *sob* and *bowl* were also identified. Interestingly, a potential recent duplication in the *H. halys* lineage was observed for an *odd*-family member. Segment polarity genes were also identified, including orthologs of key genes previously studied in *Drosophila*: *wingless*, *hedgehog*, *engrailed* and *invected*, as well as two paralogous copies of *armadillo*. Twenty-four candidate Y-linked genes were identified, including homologs to known male fertility factors in *Drosophila*, cilia- and flagella-associated proteins, and an ankyrin repeat domain-containing protein also found on the Y chromosome of various mosquito species.

The number, type distribution and organization of cuticular proteins was not remarkable with respect to other insect species: most (138 of 156 total) were R&R Consensus domain-containing CPR proteins, and approximately three-fourths of the cuticular genic repertoire was arrayed in a type-specific clustering manner. Seven aquaporin genes were identified, an amount commensurate with what has been reported in other arthropods. Please see Additional file 1 for details.

Perhaps the most striking *H. halys* genome features reflect its broad phytophagy—in particular, its remarkable abundance of chemosensory genes—as well as the diversity of genes associated with xenobiotic detoxification and digestion. Availability of the *H. halys* genome sequence will undoubtedly prove useful towards the development of environmentally sustainable biomolecular pesticides for use in concert with more traditional, synthetic chemical-based controls. In addition, given the presence of RNAi pathway components, these genomic resources can, for example, assist researchers in designing functional studies of gene function by dsRNA-mediated knockdown experiments.

The genome features described here can be directly contrasted with those of other Hemiptera with sequenced genomes, such as the brown plant hopper, *N. lugens* (Fulgoromorpha) [13], a destructive yet strictly monophagous pest of rice which has very few gene exemplars associated with chemoreception and has lost genes and gene families related to detoxification and digestion [161]. Another intriguing comparator taxon outside the Hemiptera is the wood-feeding Coleopteran pest, *Anoplophora glabripennis* (Asian long-horned beetle) [162]. These distinctions, among others, will be thoroughly explored in a follow-up comparative genomics analysis.

## Materials and Methods

### Genome sequencing, assembly and annotation

*H. halys* is one of thirty arthropod species sequenced as part of a pilot project for the i5K arthropod genomes project at the Baylor College of Medicine Human Genome Sequencing Center. An enhanced Illumina-ALLPATHS-LG sequencing and assembly strategy was used, in which four libraries of nominal insert sizes (180bp, 500bp, 3kb and 8kb) prepared from a single female insect (the homogametic sex) were sequenced, as well as one 300bp-insert library derived from a single male specimen (heterogametic sex). The amount of sequence generated from each of these libraries is noted in Additional file 1: Table S1 with NCBI SRA accessions. Both individual insects used for sequencing were the product of 10 generations of sibling-sibling breeding for genome homozygocity from the colony maintained at the USDA-ARS Beltsville Agricultural Research Center’s Invasive Insect Biocontrol and Behavior Laboratory (Beltsville, MD, USA), reared in culture as described by Khrimian et al. (2014) [163]. This colony was established in 2007 from adults collected in Allentown, PA, USA and was supplemented annually with several Beltsville, MD-collected individuals until 2011. The sibling-sibling mated individuals from this colony, from which the genome originates, are the Beijing haplotype, as confirmed using primers and haplotype conventions from Xu et al. (2014) [164]. Additional sequencing and assembly details are provided as Additional file 1. The resulting assembly has been deposited in the NCBI Genbank as assembly accession GCA_000696795.1.

Automated gene annotation was performed both with a MAKER 2.0 annotation pipeline [165] tuned specifically for arthropods and NCBI’s Eukaryotic Genome Annotation Pipeline, Gnomon [166, 167]. Manual annotation was enabled by the Apollo manual annotation and JBrowse viewing software [168, 169] hosted at The i5K Workspace [170]. Existing RNA-Seq datasets available for *H. halys* ([42, 131, 171]; see also Additional file 1: Table S1), in combination with RefSeq and GenBank protein sets from *Diaphorina citri*, *D. melanogaster*, *A. pisum* and other insects, were utilized as extrinsic evidence in preparing automated gene calls and in assisting expert annotators with refining gene models. The Additional file 2 presents gene expression levels observed within each sample reported in the aforementioned *H. halys* transcriptomics studies; RNA-Seq reads were mapped to gene models using bowtie2 [172] and expression levels (conveyed using the Transcripts Per Million (TPM) measure) were estimated by RSEM [173]. Gene annotations are distributed with the genome assembly at NCBI and are available under accession number GCA_000696795.1. The Official Gene Set halhal_OGSv1.1 is available at the i5K Workspace (https://i5k.nal.usda.gov/data/Arthropoda/halhal-(Halyomorpha_halys)/Hhal_1.0), as well as the Ag Data Commons (doi: 10.15482/USDA.ADC/1504240). Detailed information for all annotation-related topics is available in the supplement.

### Assembly and annotation completeness assessments

Completeness in terms of expected gene content of the *H. halys* genome assembly and annotated protein-coding gene set was assessed with the Benchmarking Universal Single-Copy Orthologs (BUSCO) tool, v3.0.2 [19]. The Insecta BUSCO lineage dataset (insect_odb9) was used, which consists of 1,658 single-copy orthologous genes present in at least 90% of insects at OrthoDB v9 [174]. For comparisons with other hemipterans, the same assessments were performed on the assemblies and gene sets of the pea aphid, *A. pisum* (downloaded from AphidBase [175]); the bed bug, *C. lectularius*; and the kissing bug, *R. prolixus* (obtained from VectorBase [176]). For all gene set assessments, protein files were first filtered to select only one protein per gene when alternative transcripts were annotated, always selecting the longest protein product as the representative sequence.

### Lateral gene transfers in *Halyomorpha halys*

The *H. halys* genome assembly was screened for lateral gene transfers using a DNA based homology pipeline similar to that of Wheeler et al. (2013) [177] and also with an updated version of the pipeline as described in the genome analysis of *O. fasciatus* [26].

### Chemoreceptors: Odorant, Gustatory and Ionotropic Receptors

The genome assembly was searched using tBLASTn with chemoreceptors from the most closely related hemipteran genomes available, specifically those of three other heteropterans, the milkweed bug, *O. fasciatus* [26]; the bedbug, *C. lectularius* [22]; and the kissing bug, *R. prolixus* [10]. Comparisons of the above three species with two other hemipterans, the pea aphid, *A. pisum* [11]; and the human body louse, *P. humanus corporis* [178], are available in Panfilio et al. (2019) [26]. Most gene models were built directly in the Apollo genome browser at The i5K Workspace, but problematic models and pseudogenes were built manually. Pseudogenes were translated as best as possible accommodating stop codons, frameshifts or other pseudogenizing mutations like splice mutants, but only included in the naming scheme if longer than 50% of an average family protein for the ORs and GRs, or a close relative for the more length-variable IRs. The same length criterion was applied to gene fragments thought to represent otherwise full-length genes (with some exceptions in the GR family; see below). Many gene models were joined across scaffolds, mostly based on spliced RNA-Seq reads, but sometimes on the appropriateness of gene fragments on either ends of two scaffolds. Every effort was made to complete partial gene models by repairing gaps in the genome assembly using raw RNA-Seq and/or genomic reads. Multiple alignments for each family were used to reveal problematic models, which were then manually improved. All are modeled as best as possible in the Apollo browser at i5K and were incorporated into the Official Gene Set (OGS). Their protein sequences are provided as supplementary data (see Additional file 4), as they include many genes modeled across two scaffolds and others for which the genome assembly was repaired, as well as translations of pseudogenes, none of which are available from the OGS.

The final multiple alignments for each family included the members of the three other heteropterans noted above, as well as relevant proteins from other insects, and were generated with CLUSTALX v2.1 [179]. Alignments were trimmed with TRIMAL v1.4 [180], using the “gappyout” option for the ORs and GRs, which are of generally similar length, and the “strict” option for the IRs, which commonly have highly length- and sequence-variable N-termini. Phylogenetic analysis was conducted using PHYML v3.0 [181] with default parameters. Trees were arranged and colored using FIGTREE v1.4.2 (http://tree.bio.ed.ac.uk/software/figtree/).

### Odorant-binding proteins

Odorant-binding protein (OBP) family members were searched in the *H. halys* genome scaffolds through BLAST in Apollo/JBrowse in The i5K Workspace. The OBP gene search used 30 putative HhalOBP transcripts mined in the antennae of females and males through RNA-Seq [42], as well as *H. halys* Gnomon-predicted proteins and six-frame translation products of the *H. halys* RNA collection screened against Classic, Plus-C and Atypical OBP motif-patterns (bit score 40.0). The OBP motif-patterns were built up using a set of 6,064 OBPs as a reference retrieved from NCBI by querying for “odorant binding protein”. In addition, OBP transcripts in other closely related heteropterans were also used, such as from the Miridae [182] and Pentatomidae [54]. OBP gene annotations were directly performed in the Apollo genome browser. The expression of predicted OBP genes was by qPCR, using antennae and the two forelegs of ten *H. halys* specimens from nymphs of 1^st^, 2^nd^, 3^rd^ and 4^th^ instars, unmated three day-old females and males that were killed in liquid nitrogen. Dissected antennae and legs were immediately immersed together in TRIzol and homogenized in FastPrep®-24 Instrument at 6.5 m/s for 60 s. Total RNA was extracted using PureLink RNA Mini kit (Ambion by Thermo Fisher Scientific) according to the manufacturer’s instructions, with DNase treatment on-column. RNA yield was verified using a Qubit RNA HS Assay (Thermo Fisher Scientific). One microgram of total RNA was used for first strand cDNA synthesis using SuperScript III First-Strand Synthesis System for RT-PCR (Thermo Fisher Scientific) and used for qPCR reactions (3-8 replicates each gene/isoform) using PowerUp SYBR Green Master Mix (Applied Biosystems) in Roche Applied Science LightCycler® 480 Real-Time PCR System. Primers were designed using version 2.62 of the PrimerPlex program (PREMIER Biosoft, Palo Alto, CA, USA) to make them unique and cross-homology-intolerant. Their sequences are presented in Additional file 1: Table S13.

### Vision and light detection genes

For preparation of the global opsin gene tree, *H. halys* sequences were collected by tBLASTn searches against the genome sequence draft version 1.0 (GCA_000696795.1). A multiple sequence alignment was generated with Clustal Omega [183] and variable sites were removed with Gblocks at least stringent settings [184]. A bootstrapped maximum likelihood topology was generated with RAxML on the Cipres platform [185, 186], with the cutoff for showing support values in the trees being set to 75. For preparation of the long wave-sensitive opsin gene tree, *H. halys* sequences were collected by tBLASTn searches against the NCBI TSA database; multiple alignment, variable site clearance and bootstrapped maximum likelihood analysis were performed as described above.

### Cysteine peptidases

The evolutionary history among cysteine peptidases was inferred by the Maximum Likelihood method (using the JTT matrix-based model [187]) as implemented in MEGA7 [188]. Initial tree(s) for the heuristic search were obtained automatically by applying Neighbor-Join and BioNJ algorithms to a matrix of pairwise distances estimated using a JTT model, and then a topology maximizing the log-likelihood score was selected. The analysis involved 53 amino acid sequences and a total of 145 positions in the final dataset. All positions with less than 95% site coverage (i.e., those containing at least 5% alignment gaps, missing data and ambiguous bases) were eliminated from consideration.

### Salivary effector genes

Salivary effector genes in *H. halys* were identified using a reciprocal best BLAST hit approach as described earlier for effector identification [99, 189]. Initially, aphid effectors [90, 93, 94, 190–194] were used as queries in a BLASTp search (E-value cut-off 1e-20) against *H. halys* predicted proteins. Top hits in the *H. halys* genome were BLAST searched against an *A. pisum* protein dataset to identify false positive candidates. If *H. halys* genes had a top hit different from the *A. pisum* query used in the first step, these were excluded from further analysis. Retained *H. halys* candidate genes were validated based on following three criteria: 1) presence of secretion signal peptide as revealed by signalP (version 4.1) [195], 2) absence of transmembrane domain as revealed by TMHMM (version 2) [196], and 3) presence of signature domains and/or conserved sites as revealed by an InterPro output [197], which was inspected manually. For tests of positive codon selection, the top hit for each putative *H. halys* effector was used in pairwise comparisons. Analyses were conducted using the Nei-Gojobori method [198] in MEGA X [199]. All ambiguous positions were removed from each sequence pair (pairwise deletion option) prior to analysis.

### Insect Immunity

Manual annotation efforts were organized around a list of genes involved in the innate, humoral immune response contributing to recognition, signaling and response to bacteria and fungi in arthropods. Genes were found using a combination of approaches. Immunity genes with the same gene id names (e.g., PGRP) and identified as belonging to the phylum Arthropoda (taxid:6656) were downloaded from the UniProt database and used to create an HMM profile to search against proteins identified in the genome assembly (HVIT v.1.0). Proteins with similar domains to the HMM-constructed protein families were ranked by similarity using the lowest E-values (min cutoff 1e-20; HMMER 3.1b1 May 2013 [200];) and then BLASTed against the raw genome fasta file to recover scaffold coordinates of the original protein match and any potential paralogs.

When HMM-constructed protein families were not successful in finding a match to an immunity protein of interest, a consensus sequence was manually constructed using protein sequences from UniProt containing the same gene identifier restricted to Arthropoda (taxid:6656), then compared with the genome sequence using tBLASTn and the default parameters provided by The i5K Workspace’s BLAST tool (https://i5k.nal.usda.gov/webapp/blast/). If the original search failed, default parameters were relaxed to remove the low complexity filter. Upon determination of a genomic location using one of these two methods, genes were reviewed and manually annotated. If a gene model was successfully annotated, the putative protein was compared to the NCBI NR database for arthropoda (taxid: 6656) using the BLASTp algorithm to reconfirm the annotation and gene name.

### Xenobiotic detoxification genes

Identification of *H. halys* carboxylesterase (COE) and glutathione-S-transferase (GST) enzyme inventory was performed via keyword search of Gnomon annotated *H. halys* inferred proteome. For each protein family, the resulting protein sequences were then used as queries in a BLASTp search against the inferred proteome to identify any sequences that may have been missed during the Gnomon annotation process. Results were then manually filtered on alignment quality and biological relevance. Both curated COE and GST protein sets were multiply aligned using MUSCLE [201]. SeqBoot [202] was used to create a bootstrapped data set of 100 replicates, from which a maximum likelihood-based phylogeny was generated using the method of Le and Gascuel [203] as implemented in PhyML [181]. Phylogenies were then visually rendered using the R Phytools package version 0.5-64 [204].

Cytochrome P450s (CYP) from *H. halys* were mined by batch BLAST of NCBI’s NR database using 52 P450 sequences representative of insects. Results from each search were combined and filtered to remove duplicate hits. The results were 212 gene models predicted by Gnomon from the genome. Some of these were fusions of adjacent genes that had to be split. After further refinement to split fusions and remove variants of the same gene, 141 P450s remained. To look for any additional P450s, 126 of the 141 sequences were used to BLAST search the WGS section for genomic contigs. 38,000 hits distilled to just 65 contigs, indicating P450 gene linkage. The 65 contigs were BLASTx searched against a database of named insect P450s to find all exons for P450s in the genome and to determine associated start and stop coordinates.

CYP evolutionary history was inferred by using the Maximum Likelihood method and JTT matrix-based model [187]. Initial tree(s) for heuristic search were obtained automatically by applying Neighbor-Join and BioNJ algorithms to a matrix of pairwise distances estimated using a JTT model, and then the topology with superior log likelihood value was selected. The analysis involved 126 full or nearly full-length *H. halys* sequences, as well as one *Riptortus pedestris* (CYP3231A2 ~ AK417387.1) and three *R. prolixus* (CYP315A1 ~ KQ034057.1, CYP6HK1 ~ KQ034757.1 and CYP3090A1 ~ KQ034396.1) sequences used to stabilize the position of outlier branches in the tree. All positions in the multiple sequence alignment with less than 70% site coverage were purged—that is, positions with fewer than 30% alignment gaps, missing data and ambiguous bases were allowed (partial deletion option). The final dataset contained a total of 479 positions. Evolutionary analyses were conducted in MEGA X [199].

## Supplementary information

**Additional file 1**:  Main Supplementary Information text file, including **Tables S1-S17** and **Figures S1-S18**. **Table S1**. Sequencing, assembly, annotation statistics and accession numbers. **Table S2.** OrthoDB v10 comparison of five species for ortholog presence and copy-number in Hemiptera-level orthogroups. **Table S3.** Scaffolds present in the *H. halys* assembly (accession GCA_000696795.1) that may originate from contaminant sources. **Table S4.** Counts of repetitive DNA elements encountered in the *H. halys* genome assembly. **Table S5.** *H. halys* predicted protein products associated with the RNAi pathway. **Table S6.** Positional information for the annotated homeobox genes. **Table S7.** Nuclear receptors of *H. halys*. **Table S8.** Listing of candidate Y-linked genes. **Table S9.** Number of genes identified as putative cuticle proteins per family in the genome of *H. halys*. **Table S10.** Number of genes identified as putative cuticle proteins per species in the genomes of several insect orders. **Table S11.** Clusters of genes coding for cuticle proteins in the genome of *H. halys*. **Table S12.** Odorant-binding protein genes and pseudogenes (Ψ) annotated in the genome of *H. halys*. **Table S13.** Primer sequences used to validate the HhalOBP gene annotations. **Table S14.** Correspondences between *H. halys* predicted protein identifiers and cathepsin labels. **Table S15.** A total of 64 salivary effector proteins were identified in the *H. halys* genome. **Table S16.** A select subset of 15 *H. halys* salivary effector proteins having variable expression levels between nymphal and adult stages (up- or down-regulation). **Table S17.** Gene expression data for *H. halys* glutathione S-transferase genes. **Figure S1.** Phylogenetic organization of the Hemiptera. **Figure S2.** Ortholog distributions among hemipterans. **Figure S3.** Genome assembly quality control. **Figure S4.** Hox and Iro-C cluster gene loci. **Figure S5.** *Halyomorpha mannosidase* expansion. **Figure S6.** Maximum likelihood phylogenetic tree of selected mannosidase proteins from three bacterial outgroups and three hemipteran species. **Figure S7.** Phylogenetic tree of the OR family. **Figure S8.** Phylogenetic tree of the GR family. **Figure S9.** Phylogenetic tree of the IR family. **Figure S10.** Heteropteran global opsin gene tree. **Figure S11.** Array of β-esterase genes. **Figure S12.** Distribution of transcription factor families across insect genomes. **Figure S13.** Nanos amino acid sequence alignments from different species. **Figure S14.** Location of pair-rule gene orthologs in the *H. halys* genome. **Figure S15.** *H. halys odd*-family genes. **Figure S16.** Alignment of Wnt family domain proteins. **Figure S17.** Engrailed and Invected are shared among diverse insects. **Figure S18.** Phylogenetic analysis of hemipteran genes named “NR2E1” reveals that they are orthologous to NR2E6.

**Additional file 2**. *H. halys* gene expression omnibus (per RNA-Seq data).

**Additional file 3**. *H. halys* transcription factor details.

**Additional file 4** . Manually curated *H. halys* chemoreceptor protein sequences.

**Additional file 5**. Heteropteran LW opsin sequences.

**Additional file 6**. LW opsin tuning site comparison.

**Additional file 7**. Y-linked gene sequences. 

## Data Availability

Manual annotations are distributed with the genome assembly at NCBI and are available under accession number GCA_000696795.1. The Official Gene Set halhal_OGSv1.1 is available at the i5K Workspace (https://i5k.nal.usda.gov/data/Arthropoda/halhal-(Halyomorpha_halys)/Hhal_1.0), as well as the Ag Data Commons (doi: 10.15482/USDA.ADC/1504240).
