# Supplementary material for: Brown marmorated stink bug, Halyomorpha halys (Stål), genome: putative underpinnings of polyphagy, insecticide resistance potential and biology of a top worldwide pest
Source: BMC Genomics. 2020 Mar 14;21:227. doi: 10.1186/s12864-020-6510-7 (PMC7071726; doi:10.1186/s12864-020-6510-7)
Supplement: Supplementary file 1 — Additional file 1: Main Supplementary Information text file, including Tables S1-S17 and Figures S1-S18. Table S1. Sequencing, assembly, annotation statistics and accession numbers. Table S2. OrthoDB v10 comparison of five species for ortholog presence and copy-number in Hemiptera-level orthogroups. Table S3. Scaffolds present in the H. halys assembly (accession GCA_000696795.1) that may originate from contaminant sources. Table S4. Counts of repetitive DNA elements encountered in the H. halys genome assembly. Table S5. H. halys predicted protein products associated with the RNAi pathway. Table S6. Positional information for the annotated homeobox genes. Table S7. Nuclear receptors of H. halys. Table S8. Listing of candidate Y-linked genes. Table S9. Number of genes identified as putative cuticle proteins per family in the genome of H. halys. Table S10. Number of genes identified as putative cuticle proteins per species in the genomes of several insect orders. Table S11. Clusters of genes coding for cuticle proteins in the genome of H. halys. Table S12. Odorant-binding protein genes and pseudogenes (Ψ) annotated in the genome of H. halys. Table S13. Primer sequences used to validate the HhalOBP gene annotations. Table S14. Correspondences between H. halys predicted protein identifiers and cathepsin labels. Table S15. A total of 64 salivary effector proteins were identified in the H. halys genome. Table S16. A select subset of 15 H. halys salivary effector proteins having variable expression levels between nymphal and adult stages (up- or down-regulation). Table S17. Gene expression data for H. halys glutathione S-transferase genes. Figure S1. Phylogenetic organization of the Hemiptera. Figure S2. Ortholog distributions among hemipterans. Figure S3. Genome assembly quality control. Figure S4. Hox and Iro-C cluster gene loci. Figure S5. Halyomorpha mannosidase expansion. Figure S6. Maximum likelihood phylogenetic tree of selected mannosidase proteins from three bacter [file 12864_2020_6510_MOESM1_ESM.zip › 12864_2020_6510_MOESM1_ESM/Hhalys1.0_SupplementaryMaterials.docx]

SUPPLEMENTARY INFORMATION FOR

Brown marmorated stink bug, *Halyomorpha halys* (Stål), genome:

putative underpinnings of polyphagy, insecticide resistance potential

and biology of a top worldwide pest

Michael E. Sparks^a,*^, Raman Bansal^b^, Joshua B. Benoit^c^, Michael B. Blackburn^a^, Hsu Chao^d^, Mengyao Chen^e^, Sammy Cheng^f^, Christopher Childers^g^, Huyen Dinh^d^, HarshaVardhan Doddapaneni^d^, Shannon Dugan^d^, Elena N. Elpidina^h^, David W. Farrow^c^, Markus Friedrich^i^, Richard A. Gibbs^d^, Brantley Hall^j^, Yi Han^d^, Richard W. Hardy^k^, Christopher J. Holmes^c^, Daniel S.T. Hughes^d^, Panagiotis Ioannidis^l,m^, Alys M. Cheatle Jarvela^e^, J. Spencer Johnston^n^, Jeffery W. Jones^i^, Brent A. Kronmiller^o^, Faith Kung^e^, Sandra L. Lee^d^, Alexander G. Martynov^p^, Patrick Masterson^q^, Florian Maumus^r^, Monica Munoz-Torres^s^, Shwetha C. Murali^d^, Terence D. Murphy^q^, Donna M. Muzny^d^, David R. Nelson^t^, Brenda Oppert^u^, Kristen A. Panfilio^v,w^, Débora Pires Paula^x^, Leslie Pick^e^, Monica F. Poelchau^g^, Jiaxin Qu^d^, Katie Reding^e^, Joshua H. Rhoades^a^, Adelaide Rhodes^y^, Stephen Richards^d,z^, Rose Richter^f^, Hugh M. Robertson^aa^, Andrew J. Rosendale^c^, Zhijian Jake Tu^j^, Arun S. Velamuri^a^, Robert M. Waterhouse^ab^, Matthew T. Weirauch^ac,ad^, Jackson T. Wells^o^, John H. Werren^f^, Kim C. Worley^d^, Evgeny M. Zdobnov^l^ and Dawn E. Gundersen-Rindal^a,*^

^a^ USDA-ARS Invasive Insect Biocontrol and Behavior Laboratory, Beltsville, MD 20705 USA

^b^ USDA-ARS San Joaquin Valley Agricultural Sciences Center, Parlier, CA 93648 USA

^c^ Department of Biological Sciences, University of Cincinnati, Cincinnati, OH 45221 USA

^d^ Human Genome Sequencing Center, Department of Human and Molecular Genetics, Baylor College of Medicine, Houston, TX 77030 USA

^e^ Department of Entomology, University of Maryland, College Park, MD 20742 USA

^f^ Department of Biology, University of Rochester, Rochester, NY 14627 USA

^g^ USDA-ARS National Agricultural Library, Beltsville, MD 20705 USA

^h^ A.N. Belozersky Institute of Physico-Chemical Biology, Moscow State University, Moscow 119911 Russia

^i^ Department of Biological Sciences, Wayne State University, Detroit, MI 48201 USA

^j^ Department of Biochemistry, Virginia Tech, Blacksburg, VA 24061 USA

^k^ Department of Biology, Indiana University, Bloomington, IN 47405 USA

^l^ Department of Genetic Medicine and Development, University of Geneva Medical School and Swiss Institute of Bioinformatics, Geneva, 1211, Switzerland

^m^ Present address: Institute of Molecular Biology and Biotechnology, Foundation for Research and Technology-Hellas, 73100 Heraklion, Crete, Greece

^n^ Department of Entomology, Texas A&M University, College Station, TX 77843 USA

^o^ Center for Genome Research and Biocomputing, Oregon State University, Corvallis, OR 97331 USA

^p^ Center for Data-Intensive Biomedicine and Biotechnology, Skolkovo Institute of Science and Technology, Skolkovo 143025 Russia

^q^ National Center for Biotechnology Information, National Library of Medicine, National Institutes of Health, Bethesda, MD 20894 USA

^r^ URGI, INRA, Université Paris-Saclay, 78026 Versailles, France.

^s^ Environmental Genomics and Systems Biology Division, Lawrence Berkeley National Laboratory, Berkeley, CA 94720 USA.

^t^ Department of Microbiology, Immunology and Biochemistry, University of Tennessee Health Science Center, Memphis, TN 38163 USA

^u^ USDA-ARS Center for Grain and Animal Health Research, Manhattan, KS 66502 USA

^v^ Institute for Zoology: Developmental Biology, University of Cologne, 50674 Cologne, Germany

^w^ School of Life Sciences, University of Warwick, Gibbet Hill Campus, Coventry CV4 7AL, United Kingdom

^x^ EMBRAPA Genetic Resources and Biotechnology, Brasília, DF, Brazil 70770-901

^y^ Larner College of Medicine, The University of Vermont, Burlington, VT 05452 USA

^z^ Present address: Earth BioGenome Project, University of California, Davis, Davis, CA 95616 USA

^aa^ Department of Entomology, University of Illinois, Urbana-Champaign, IL 61801 USA

^ab^ Department of Ecology and Evolution, University of Lausanne and Swiss Institute of Bioinformatics, Lausanne, 1015, Switzerland

^ac^ Center for Autoimmune Genomics and Etiology, Division of Biomedical Informatics, and Division of Developmental Biology, Cincinnati Children's Hospital Medical Center, Cincinnati, OH 45229 USA

^ad^ Department of Pediatrics, College of Medicine, University of Cincinnati, Cincinnati, OH 45267 USA

^*^ Authors for correspondence: michael.sparks2@usda.gov ; dawn.gundersen-rindal@usda.gov

**Genome sequencing, assembly and annotation**

The genome size was estimated following methods described in Hare and Johnston (2011) [1], with the head of a male or female *H. halys* combined with 1/3 of the head of a male *Periplaneta americana* standard 1C = 3.338 Mb in a 2-ml Dounce tissue grinder with 1ml of Galbraith buffer, ground with 15 strokes of the A pestle, filter through 45 um nylon mesh and stained for 1 hour in the cold and dark with 25 ug/ml of propidium iodide. Relative red fluorescence of the 2C peaks of the sample and standard were scored using a Partec Cyflow with excitation at 514 nm. DNA amount was estimated as the ratio of the 2C sample to the 2C standard times 3.338 Gb.

An enhanced Illumina-ALLPATHS-LG sequencing and assembly strategy was used, in which four libraries—made from DNA isolated from the product of 10 generations of sibling-sibling breeding—of nominal insert sizes (180bp, 500bp, 3kb and 8kb) were sequenced (see Additional file 1: Table S1). A gel-cut paired end library protocol was used to prepare the 180bp and 500bp libraries. Briefly, 1 µg of DNA was sheared using a Covaris S-2 system (Covaris, Inc. Woburn, MA) using the 180bp or 500bp program. Sheared DNA fragments were purified with Agencourt AMPure XP beads, end-repaired, dA-tailed, and ligated to Illumina universal adapters. After adapter ligation, DNA fragments were further size selected by agarose gel and PCR amplified for six to eight cycles using Illumina P1 and Index primer pair and Phusion® High-Fidelity PCR Master Mix (New England Biolabs). The final library was purified using Agencourt AMPure XP beads and quality assessed by Agilent Bioanalyzer 2100 (DNA 7500 kit) to determine library quantity and fragment size distribution before sequencing.

Long-mate pair libraries with 3kb or 8kb insert sizes were constructed per manufacturer’s protocol (Mate Pair Library v2 Sample Preparation Guide art # 15001464 Rev. A PILOT RELEASE). Briefly, 5 µg (for 2 and 3 kb gap size library) or 10 µg (8-10 kb gap size library) of genomic DNA was sheared to desired size fragments by Hydroshear (Digilab, Marlborough, MA), then end repaired and biotinylated. Fragment sizes between 3.0-3.7 kb (3kb) or 8-10 kb (8kb) were purified from 1% low melting agarose gel and then circularized by blunt-end ligation. These size selected circular DNA fragments were sheared to 400 bp (Covaris S-2), purified using Dynabeads M-280 Streptavidin Magnetic Beads, end-repaired, dA-tailed, and ligated to Illumina PE sequencing adapters. DNA fragments with adapter molecules on both ends were amplified for 12 to 15 cycles with Illumina P1 and Index primers. Amplified DNA fragments were purified with Agencourt AMPure XP beads. Quantification and size distribution of the final library was determined before sequencing as described above.

Sequencing was performed on Illumina HiSeq2000s (Casava Version 1.8.3_V3) generating 100bp paired end reads. Reads were assembled using ALLPATHS-LG (v35218) [2] on a computer with 1Tbyte of RAM and further scaffolded and gap-filled using Atlas-Link (v.1.0) and Atlas gap-fill (v.2.2) (https://www.hgsc.bcm.edu/software/). The resulting assembly has been deposited in the NCBI Genbank as assembly accession GCA_000696795.1.

As an additional quality check of the *H. halys* annotated gene set we compared distributions of orthologs in four other hemipteran species chosen for having high quality, published and stable official gene sets: the pea aphid (*Acyrthosiphon pisum*), kissing bug (*Rhodnius prolixus*), bed bug (*Cimex lectularius*) and milkweed bug (*Oncopeltus fasciatus*). Comparisons were performed on the orthology dataset defined at the Hemiptera node from the OrthoDB v10 catalog of orthologs [3], which comprises a total of 16 species. *H. halys* compares favorably with other species for both gene presence and for ortholog copy-number across orthogroups (Additional file 1: Table S2). All four ingroup species have good orthogroup representation (row A, ≥90%) in near-universal orthogroups (note the pea aphid is expected to have lower representation because of its position as the outgroup species in this analysis) and *H. halys* (98.9%) has the best representation. This is also reflected in the lowest number of orthogroups with single-copy orthologs in all other species but missing an ortholog in *H. halys* (B, 46) and similarly in the lowest number of orthogroups with single- and multi-copy orthologs in the other species but missing an ortholog in *H. halys* (C, 28). With respect to rare duplications—defined as multi-copy orthologs in groups where all four other taxa have single-copy orthologs—*Halyomorpha* (D, 179) shows slightly more than *Rhodnius* and *Cimex*, and fewer than *Oncopeltus*, all of which are much lower than for the pea aphid (which is known to have a high level of gene duplications). Overall, *H. halys* shows the most genes in near-universal orthogroups (E, 9,295), reflecting both the intermediate level of duplications and the lower numbers of missing orthologs observed. Analysis of hemipteran ortholog distributions from the OrthoDB v10 Hemiptera node identified almost 5,000 orthogroups with on average more than 6,000 genes per species that are common across all five selected hemipterans (Additional file 1: Figure S2).

The pipeline originally developed by Wheeler et al. (2013) ([4], with updated versions described in the genome analyses of *O. fasciatus* [5], *Hyalella aztec*a [6] and *Diachasma alloeum* [7]) was used both to detect bacterial contaminants and lateral gene transfers (discussed below) in *H. halys*. The DNA-based pipeline detected 21 scaffolds that are likely bacterial contaminants (Additional file 1: Table S3)—these were generally short, with the largest being 4,569bp in length.

As a complement to the Wheeler-Werren pipeline, a BlobTools [8] analysis was also performed. (The two methods have both been applied to the contamination analysis of *D. alloeum* [7], wherein the Wheeler-Werren pipeline detected a larger number of bacterial scaffolds (635 versus 491) and correctly identified one scaffold misclassified by BlobTools as a bacterial contaminant—as deduced by follow-up manual annotation—as containing a LGT inserted in the insect scaffold. However, BlobTools also screens for potential fungal contaminants, *inter alia*, which the Wheeler-Werren pipeline does not.) To enable BlobTools coverage assessments, all sequenced DNA reads were aligned to genomic scaffolds using Bowtie 2 [9] in paired-end mode with default parameters. To support taxonomic assessments, all genomic scaffolds were compared with the NCBI NR protein database using DIAMOND in its BLASTx-like mode [10] with default parameter settings. DIAMOND output was post-processed to remove hits to known *H. halys* protein sequences, as retaining these may have served to artificially inflate counts of scaffolds classified as “Arthropoda.” A visual representation of the BlobDB object computed from these data is presented as Additional file 1: Figure S3, which demonstrates a clean distribution of data, with scaffolds being consistently placed in narrow bands of sequencing coverage and [GC]. This supports the notion that the assembly contains very few contaminant sequences, as these would tend to exhibit quite different coverages and nucleotide compositions relative to *H. halys* genomic DNA (i.e., they would be exceptional outliers). Following scrutinous manual inspection of scaffolds classified as bacterial, fungal or plant in origin, an additional 40 scaffolds were flagged as being suspect (Additional file 1: Table S3). A total of six predicted protein-coding genes (none of which were used in the analyses presented in this report) were found on these scaffolds and subsequently purged from the Official Gene Set (see below): XP_014293456.1, XP_014293507.1, XP_014293515.1, XP_014293561.1, XP_014293659.1 and XP_014293720.1.

*Using a MAKER 2.0 Pipeline Tuned for Arthropods:*

The *H. halys* genome assembly was subjected to automatic gene annotation using a MAKER 2.0 annotation pipeline [11] tuned specifically for arthropods. The pipeline is designed to be systematic, providing a single consistent procedure for species (such as *H. halys*) included in the i5k pilot study, scalable to handle hundreds of genome assemblies, guided using both protein and RNA-Seq evidence as extrinsic support for gene models, and targeted to utilize extant information on arthropod gene sets. The core of the pipeline was a MAKER 2 instance, modified slightly to enable efficient execution on available computational resources. The genome assembly was first subjected to *de novo* repeat prediction and CEGMA [12] analysis to generate gene models for initial training of the *ab initio* gene predictors. Three rounds of training of the Augustus [13] and SNAP [14] gene predictors within MAKER were used to bootstrap a training data set. Input protein data included one million peptides from a non-redundant reduction (90% identity) of Uniprot Ecdysozoa (1.25 million peptides) supplemented with proteomes from eighteen additional species (*Strigamia maritime, Tetranychus urticae, Caenorhabditis elegans, Loa loa, Trichoplax adhaerens, Amphimedon queenslandica, Strongylocentrotus purpuratus, Nematostella vectensis, Branchiostoma ﬂoridae, Ciona intestinalis, Ciona savignyi, Homo sapiens, Mus musculus, Capitella teleta, Helobdella robusta, Crassostrea gigas, Lottia gigantean* and *Schistosoma mansoni*) leading to a final non-redundant peptide evidence set of 1.03 million peptides. RNA-Seq transcription data derived from adult males, females and mixed sex juveniles [15] was used to judiciously identify exon-intron boundaries in conjunction with a heuristic script to identify and split erroneously joined gene models. We used CEGMA models for QC purposes: for 1,977 CEGMA single-copy ortholog gene models, 1,869 were found in the assembly and 1,847 in the final predicted gene set. Finally, the pipeline uses a nine-way homology prediction with human, *Drosophila* and *C. elegans*, and InterPro Scan5 to allocate gene names. The automated gene sets are available from the BCM-HGSC website (https://www.hgsc.bcm.edu/arthropods/brown-marmorated-stink-bug-genome-project[)](https://www.hgsc.bcm.edu/arthropods/western-flower-thrips-genome-project)) and the National Agricultural Library (https://i5k.nal.usda.gov/Halyomorpha_halys) where a web browser of the genome, annotations and supporting annotation data is accessible.

*NCBI Eukaryotic Genome Annotation Pipeline:*

Structural annotation of genes was also performed in a wholly MAKER-independent manner with NCBI’s Eukaryotic Genome Annotation Pipeline. This automated annotation pipeline generates gene annotations from a combination of transcript and protein alignment evidence supplemented with *ab initio* predictions [16,17]. Existing RNA-Seq datasets available for *H. halys* from a variety of life stages, sexes and tissue types ([15,18,19]; see also Additional file 1: Table S1); in combination with RefSeq and GenBank protein sets from *Diaphorina citri*, D. melanogaster, *A. pisum* and other insects; were aligned to the genome and used to inform gene model predictions made by the NCBI eukaryotic gene prediction tool, Gnomon. Assembled *H. halys* transcript sequences available in GenBank [15,18,19] were integrated into some transcript models by Gnomon to partially compensate for gaps in the Hhal_1.0 genome assembly; this “gap-filling” process was applicable to roughly 15% of the inferred *H. halys* gene space, consistent with rates observed in other species. An overview of the annotation release (*H. halys* annotation release 100) is available online at <https://www.ncbi.nlm.nih.gov/genome/annotation_euk/Halyomorpha_halys/100/>.

*Manual, expert-level annotation:*

Manual correction of computational gene predictions represents a crucial step in generating high-quality genome annotations. The i5k Workspace [20] facilitated manual curation via the Apollo manual annotation and JBrowse viewing software [21,22]. To provide extrinsic evidence for gene model improvement RNA-Seq reads from antennae [18]; 2^nd^ and 4^th^ instars, female and male adults [15]; and pooled tissues [19] were aligned to the genome assembly using STAR [23]. In addition, read data from Sparks et al. (2014) [15] were globally assembled using Trinity (version 2.1.0, [24]) and aligned to genomic templates using GMAP (version 2015-09-29, [25]). Annotators from the *H. halys* research community received training on the use of Apollo, and were asked to adhere to a standard set of guidelines (https://i5k.nal.usda.gov/content/rules-web-apollo-annotation-i5k-pilot-project). Annotators modified or added 1,067 gene models, comprising 1,232 transcripts.

*Official Gene Set Generation:*

Manual annotations were quality-controlled (QC) using the GFF3toolkit software [26] (https://github.com/NAL-i5K/GFF3toolkit/releases/tag/v1.4.4), specifically the gff3_QC and gff3_fix functions, in addition to manual review. These were merged with the NCBI *Halyomorpha halys* annotation release 100 (https://www.ncbi.nlm.nih.gov/genome/annotation_euk/Halyomorpha_halys/100/) using the gff3_merge function of the GFF3toolkit, resulting in halhal_OGSv1.1. Manual annotations received i5k Workspace identifiers, and received an additional round of QC using NCBI’s linux64.table2asn_GFF program (ftp://ftp.ncbi.nih.gov/toolbox/ncbi_tools/converters/by_program/table2asn_GFF/linux64.table2asn_GFF.gz, downloaded March 2018). Manual annotations are distributed with the genome assembly at NCBI and are available under accession number GCA_000696795.1. The Official Gene Set halhal_OGSv1.1 is available at the i5k Workspace (https://i5k.nal.usda.gov/data/Arthropoda/halhal-(Halyomorpha_halys)/Hhal_1.0), as well as the Ag Data Commons (doi: 10.15482/USDA.ADC/1504240).

**Lateral gene transfers in *Halyomorpha halys***

In the genome analysis of the milkweed bug, an LGT from *Wolbachia* into the common ancestor of the milkweed bug and *H. halys* (both members of the infraorder Pentatomomorpha) was detected, involving an endo-1,4-beta-mannosidase, with subsequent expansion of the gene into a nine-member family in *H. halys* (XP_014289360, XP_014289359, XP_014289361, XP_014289363, XP_014289366, XP_014289370, XP_014289368, XP_014289367 and XP_014289364; see Supp. Fig S5). The expansion of this gene family and retention of open reading frames along their length suggest possible functions in *H. halys*, although those have yet to be determined. Additional LGT candidates detected in *H. halys* are described below. Although these all require further validation and investigation, strong evidence of expression for events LGT1-LGT3, and similarity to *H. halys* gene models, lends support for these as possible LGT events that have led to acquisition of genes that have evolved function in *H. halys*. The majority of these are ankyrin-repeat bearing genes from *Wolbachia*, suggesting a propensity of *H. halys* to utilize ankyrin-repeat gene transfers for function during evolution.

LGT1: Ankyrin-repeat protein gene from *Wolbachia* followed by gene family expansion to four members. This LGT matches several *Wolbachia* genomes with similar levels of homology, so the precise source *Wolbachia* is undetermined. The best match is to *Drosophila incompta* strain wInc_SM [27] and protein match is to Ankyrin-Repeat Protein (e.g., WP_1361322836.1). The *H. halys* members of this gene family are LOC106679496, LOC106677816, LOC106679233 and LOC106680616. Each gene model shows expression in all the life stages tested 2^nd^ and 4^th^ instar, male and female adults. Maintenance of these open reading frames with matches to the *Wolbachia* ankyrin-repeat proteins over much of their length implies selection for function, indicating that further analysis of their evolution and possible function is warranted.

LGT2: A second independent *Wolbachia* ankyrin-repeat protein gene transfer has occurred, which corresponds to gene model LOC106688239 in *H. halys*. The conclusion that this is an independent LGT is based on different matches to *Wolbachia* ankyrin genes and absence of reciprocal matches to LGT1 members. The best match is to the genome of *Wolbachia* wAlbB, which is a member of the B supergroup of *Wolbachia* [28], in contrast to the A-group match found for LGT1. The LGT gene is expressed in 2^nd^ instar, 4^th^ instar, male imago and female imago.

LGT3: A third independent *Wolbachia* transfer has occurred, involving the *Wolbachia* phage region (best match to fragement 2 of WOcauB3); this is also an ankyrin-repeat bearing protein. The LGT subsequently has undergone a tandem gene duplication in *H. halys* (LOC106681544 and LOC106681543), and the genes are expressed in 2^nd^ instar, 4^th^ instar, male imago and female imago.

LGT4: A fourth independent *Wolbachia* transfer has occurred, corresponding to gene model LOC106689219, which encodes a single, uncharacterized protein (XP_014289594.1). At least two paralogous copies of this gene are evident in the H. halys assembly: LOC106679174 (encoding XP_014273660.1) and LOC106679173 (encoding protein isoforms XP_014273659.1 and XP_014273658.1).

LGT5: A candidate LGT for *Candidatus* *Pantoea carbekii* is found in NW_014466461.1 and corresponds to the ribonuclease III gene from that bacterium. There is only trace gene expression in this region, and the high DNA similarity suggests a relatively recent transfer. Insect genes flank the putative LGT in the scaffold, but confirmation of the assembly by polymerase transfer amplification of the junction between the LGT and insect genes has not been done. Combined with absence of RNA sequencing support, this suggests it is either a relative recent LGT or an assembly error, requiring further validation.

LGT6: This also shows sequence homology to *Ca. P. carbekii*, is on NW_014468021.1, and lacks expression support. As with LGT5, this may be a recent LGT or an assembly artifact, and requires further validation.

**Repetitive sequences**

Repetitive elements contribute 281 Mb (i.e., 24% of the total assembly and 28% of the non-gapped assembly). The most abundant repeats are transposable elements (TEs), representing 190 Mb of the genome assembly, with LINE-type non-LTR retrotransposons being clearly predominant (124 Mb; see Additional file 1: Table S4). Unclassified interspersed repeats also contribute significantly to the repetitive fraction (91 Mb). The repetitive content detected in the assembly is relatively low among insects with similar genome size [29]. Highly similar, repetitive elements are especially challenging to assemble, suggesting that a fraction of the predicted sequences missing in the assembly could correspond to repeats. Therefore, repetitive elements were quantified directly from a library of sequencing reads. For each repetitive element detected in the assembly, the number of matching reads was compared to that obtained using an equal number of reads randomly generated from the actual assembly. Remarkably, we found that one specific Gypsy-type LTR retrotransposon is heavily under-represented in the assembly, suggesting that this TE family may have only recently begun accumulating in the genome.

*Associated Materials and Methods:* For the annotation of repetitive elements, we constructed a library of consensus sequences representative of repetitive elements present in the genome assembly using TEdenovo from the REPET package (version 2.4; [30]). TEdenovo was launched on a subset of the whole assembly comprising only the contigs of length greater than 25 kb (representing 366 Mb) and produced a library of 549 elements. This library has been characterized using REPET’s built-in utility followed by an in-house script to propose a classification for each consensus. In addition, TEannot [31] from the REPET package was launched a first time to filter out consensus sequences without a single full-length match on the assembly subset used for TEdenovo. The final REPET library (461 sequences) was then used to annotate the whole assembly. A complementary analysis was performed using the RepeatModeler package [32], which identified 1,941 *H. halys*-specific repetitive DNA consensus sequence models, the multiple sequence alignments for which are publicly available at the Dfam database [33].

For quantification of repetitive elements in sequencing reads, we analyzed repetitive elements from the SRA experiment SRX552653. Reads were quality-checked and processed using Trimmomatic [34] resulting in about 65 million read pairs. From the actual assembly, we have simulated an equal number of read pairs using wgsim [35]. Sequencing reads and simulated reads were then compared to the REPET library using BLASTn. Reads were assigned to a consensus sequence according to their best hit score against the library. Finally, we have compared the number of reads and simulated reads corresponding to each consensus sequence.

**Chemoreceptors: Odorant, Gustatory and Ionotropic Receptors**

The full set of annotated *H. halys* chemoreceptor protein sequences is available in Additional file 4. Detailed observations for each receptor family follow below.

*The odorant receptor family:*

The OR family in *H. halys* consists of the usual single ortholog for the highly conserved Orco protein and 148 “specific” ORs, which is slightly larger than the other four heteropterans, with bedbug being the smallest by at least half. Like *Oncopeltus*, *Cimex* and *Pediculus*, there is only one obvious instance of alternative splicing yielding two isoforms (Table 2 of main text), with HhalOr8a/b having RNA-Seq support for the splicing of the first long exon of HhalOr8a into the final three exons shared with Or8b, which is in a different scaffold. Only one clear pseudogene was noted (HhalOr63P). Eight models involve joins across scaffolds, mostly with RNA-Seq support. Models for twenty partial genes were successfully completed by repair of the genome assembly, while just one (Or15) remains partial with the final four short exons missing. This OR set is therefore one of the most complete available for any insect.

Phylogenetic analysis of the OR family in the four heteropterans (Additional file 1: Figure S7) reveals only a single instance of single-copy orthologs amongst the “specific” ORs (i.e., HhalOr72/OfasOr82/ClecOr1/RproOr1). All remaining relationships, as is typical for ORs, involve duplications or losses in at least one species. As expected from their closer phylogenetic relationship, in almost every instance ORs from *Halyomorpha* and *Oncopeltus* are closest relatives. *Halyomorpha* appears to have lost at least seven OR lineages, but also has major expansions of three lineages (Additional file 1: Figure S7). The first comprises Or7-24, encoding 19 proteins via alternative splicing of Or8, and most of these genes are on different scaffolds, indicating that they are spread around the genome. The second expansion comprises Or86-106 or 21 proteins, again mostly on separate scaffolds, although Or86-93 are in a tandem array on NW_014467636.1. The largest expansion is Or32-72 comprising 40 genes. Of these, Or33-35 and 64-72 are all singletons in different scaffolds; however, Or58-63 are in a tandem array in the first 112kb of 280kb NW_014467977.1, while Or36-57 are in a large array, not exclusively in tandem orientation, spanning 1.063Mb from roughly 1.403Mb in 1.640Mb NW_014466444.1 to 826kb in 1.046Mb NW_014466499.1. Or36-57 are also the youngest expansion in this family, with short branches to most proteins.

*The gustatory receptor family:*

The GR family in *H. halys* consists of 198 genes potentially encoding 347 proteins through alternative splicing of a long first exon into a shared set of three short exons encoding the C-terminus in 63 genes, ranging from two isoforms to 13 (Gr81a-m). At least ten additional long first exons were not included in the analyzed set, because without knowledge of the set of short C-terminal exons they belong with they do not align well, so the family is likely to be somewhat larger. Three models were joined across scaffolds, while 12 involved repair of the genome assembly. Sixteen models remain partial with N-terminal, C-terminal or internal exons missing. Thirty-seven of these possible proteins are pseudogenic, leaving a potentially functional set of 330 GR proteins.

The large and ancient GR family has three highly conserved subfamilies, present throughout insects: the carbon dioxide, sugar and fructose receptor subfamilies. Like *Oncopeltus* and *Cimex*, *Halyomorpha* has a set of GRs related to the carbon dioxide receptors of endopterygotes (represented by DmelGr21a/63a and TcasGr1-3 in Additional file 1: Figure S8); however, this subfamily has been considerably expanded in *Halyomorpha* to 14 proteins. It remains to be determined whether these proteins in hemipterans are involved in perception of this molecule, or whether this is simply the GR lineage from which the carbon dioxide receptors of endopterygotes evolved. Like *Oncopeltus*, *Halyomorpha* has three members of the sugar receptor subfamily (represented in Additional file 1: Figure S8 by AmelGr1/2), although only two of these are orthologs, indicating that their ancestor had four sugar receptors and each species independently lost one of them. This sugar receptor subfamily was completely lost from the blood-feeding *Rhodnius* and *Cimex*. Like the other hemipterans, *Halyomorpha* has a single member of the fructose receptor lineage expressed both peripherally and in the brain in *Drosophila* flies [36]. The remaining GRs form several major clades of expansions in *Halyomorpha* and *Oncopeltus*, sometimes with representatives from *Rhodnius* and/or *Cimex*. The far larger size of the GR family largely results from expansions of these lineages, including both gene duplications and widespread alternative splicing leading to multiple isoforms for 63 genes. The availability of considerable RNA-Seq evidence not only allowed repair of many gene models, but also provided support for many of these alternative splices—for example, four of the five modeled isoforms of Gr159 have spliced reads supporting their splicing into the final three short shared exons encoding the C-terminus. The largest of the *Halyomorpha*-specific expansions is 108 proteins (16 of them pseudogenic) related to a single *Oncopeltus* protein (OfasGr50). By inference from endopterygotes, these candidate bitter taste GRs are most likely involved in perception of plant secondary compounds, and the massive expansion of this subset of *Halyomorpha* GRs is likely related to their highly polyphagous nature.

*The ionotropic receptor family:*

The IR family in *H. halys* has 39 members, approximately the same number of genes as those of the other heteropterans. One of these genes required repair of the genome assembly, and five others remain as partial models. They include single conserved orthologs for the two well-known co-receptors, Ir8a and 25a, as well as another co-receptor Ir76b [37,38] (Additional file 1: Figure S9). There are also single orthologs for the Ir21a, 40a, 68a and 93a genes that are present in more basally-branching insects [39] and, at least in *Drosophila,* mediate perception of temperature and humidity [40–42]. However, Ir68a appears to be a pseudogene with a stop codon in the middle of exon7. This stop codon is present in the raw reads from the genome project, as well as a single RNA-Seq read from the same strain amongst 125Gb of RNA-Seq in the SRA, but it is possible that it is unique to this strain, as this protein is involved in perception of high humidity, at least in *Drosophila* flies, and hence is unlikely to be pseudogenic in the wild. There are four members of the Ir41 clade (which includes DmelIr41a, 76a, and 92a), implicated in perception of amines in *Drosophila* [43,44], and 10 of the Ir75 clade (which includes DmelIr75a-d, 64a, and 84a), involved in perception of acids in *Drosophila* [45–49], with complexity comparable to the other heteropterans in each case. The remaining IRs are numbered from 101 to avoid confusion with *D. melanogaster* IRs which only go up to Ir100a as they were named for their cytological locations. They form two quite separate groupings. Ir101-113 are either intronless or have a few idiosyncratic introns, like their relatives in the other heteropterans, and are distantly related to the DmelIr7a-g/11a genes and the Ir20a clade in *Drosophila*, both of which are implicated in gustation [50–53]. Finally, HhalIr114-118 have many introns and like their intron-bearing heteropteran relatives, form a distinct lineage with no *Drosophila* relatives, so little can be said about their possible roles.

**Insect Immunity**

### *Peptidoglycan* *receptor proteins:*

Insects utilize a variety of pathogen recognition receptors (PRRs) to detect pathogen-specific molecular motifs. For example, peptidoglycan receptor proteins (PGRPs) recognize the peptidoglycans present in cell walls of Gram-positive and Gram-negative bacteria [54]. PGRPs are highly conserved, with mammals and animals sharing a domain of approximately 160 amino acids [54]. Unlike the pea aphid, which has no homologs to PGRP, one putative PGRP is evident in *H. halys* (NW_014466636.1:681240-694476). Per the SmartBLAST algorithm at NCBI, one gene was most closely related to the peptidoglycan-recognition S2-like protein in the squash bug, *Anasa tristis* (AFM38196.1), and it encodes the conserved domain for the peptidoglycan recognition protein precursor.

#### *Gram-negative binding proteins:*

Gram-negative binding proteins (GNBPs) are thought to also detect Gram-positive bacteria [55]. GNBPs hydrolyze Gram-positive peptidoglycans into small fragments, which are detected by PGRPs. In the brown marmorated stink bug, two beta-1,3-glucan recognition proteins were identified with similarity to known GNBP proteins from the termite, *Zootemorpsis nevadensis* (KDR21214.1, matching NW_014467030.1: 31846-44840) and the squash bug (AFM38191.1, matching NW_014467598.1:133928-139347). The pattern recognition may not be limited to bacteria, as β-1,3-glucans are components of fungal cell walls that can also serve as a pattern recognition signal [56]. The presence of multiple versions of these genes may be an indicator that these proteins are involved in bacterial and/or fungal recognition and response immunity pathways.

#### *Lectins:*

One putative lectin, galectin, was annotated on NW_014466434.1:404096-411448, exhibiting highest similarity to a galectin of the mosquito, *Anopheles darlingi* (ETN66847.1). Lectins interact with compounds produced by the prophenoloxidase cascade to opsonize invading particles and make them vulnerable to phagocytosis. Galectins can also be exploited by parasites such as *Leishmania major* to bind to the midgut of their insect hosts [57]. No hemocytins were uncovered during manual annotation efforts.

### *Antimicrobial peptides:*

At the response end of the pathway, homologs for inducible antibacterial peptides were found for defensin and hemipticerin. Cecropin, andropin and pyrhoccoricin antimicrobial peptides were not observed. Defensin appears after bacterial challenge or injury in the hemolymph of insects and possess anti-Gram-positive activity [58]. The closest homolog to the annotated defensin in *H. halys* belonged to the body louse, *Pediculus humanus corporis* (XP_002428138.1). Two homologs of hemipticerin, an antimicrobial peptide isolated from the hemipteran sap-sucking bug *Pyrrhocoris apterus*, were found.
 No evidence was found for cecropin in the *H. halys* genome. This lack of cecropin may allow bacteria to escape detection by the antimicrobial response pathway. Antimicrobial peptides derived from insect cecropins have been used to engineer innate immune defenses in plants [59]. Cecropin has been found to be an effective defense against Pierce’s disease, caused by the bacterium *Xylella fastidiosa*, which is carried by the glassy-winged sharpshooter *Homalodisca vitripennis* [60]. A lack of cecropin may prevent the glassy-winged sharpshooter antimicrobial response to be signaled to clear a bacterial pathogen from its system. It is possible that the brown marmorated stink bug also has a microbial invader that overcomes its innate immunity due to the lack of cecropin.

*RNA interference:*

The RNA interference (RNAi) pathway is an important component of the insect immune system which can also be used to “knock down” or “silence” the expression of targeted genes. The *H. halys* transcriptome contains the major components of the RNAi pathway [15], which can now be mapped to their genomic loci. Identification of *H. halys* RNAi machinery was performed via keyword search of the Gnomon-annotated *H. halys* inferred proteome, as well as BLASTp searches using exemplar RNAi component sequences. Several of the gene families investigated in this paper have also been used as targets for RNAi knockdown studies in other insect systems, including carboxylesterases [61,62], cytochrome P450s [63,64] and glutathione S-transferases [63]. Many of these knockdown studies also investigate the effects of the knockdown, such as a reduction of insecticide tolerance, fecundity and/or survivorship.

Three “Dicer” enzymes were observed in the inferred proteome: one was annotated as dcr-1 and the other two as Dicer isoforms. None were annotated as dicer-2, the enzyme responsible for cleaving dsRNA and shRNA to the proper length for RISC, although Sparks et al. (2014) [15] reported a dicer-2 transcript observed in RNA-Seq data. Many RISC associated components, such as Argonaute, Aubergine, FMR1, VIG and TARBP, as well as numerous RNA helicases are present in the *H. halys* inferred proteome (Additional file 1: Table S5). No infered SID-1 or SID-2 proteins were identified. Because this analysis relied on automated gene annotations derived (at least in part) from genomic DNA data, potentially missing RNAi machinery may be explained by false negative predictions from the gene finding algorithm or by their encoding loci not being represented in the current draft assembly.

Recent *in vivo* work has shown that RNAi is effective for knocking down gene expression in *H. halys* [65,66]. Both *in silico* and *in vivo* confirmation of *H. halys*’ RNAi machinery strengthens the argument for current and future efforts to develop RNAi control technologies. Several RNAi gene silencing studies on aphids have yielded promising results—these studies target several of the gene families explored here. Plant-mediated gene silencing in wheat has been shown effective in reducing E4 carboxylesterase levels in the aphid *Sitobion avenae*, rendering it more susceptible to Phoxim insecticides [62]. Gong et al. (2014) [61] demonstrated that oral-mediated RNAi knockdown of an *A. gossypii* carboxylesterase gene reduced organophosphorus insecticide resistance.

#### *Miscellaneous immunity genes:*

We found two potential lysozymes and three potential chitinases (the latter of which may, in addition or alternatively, be involved in the insect’s molt cycle [67]). No Turandot was found, or any other variations such as TotX or TotM. Six putative prophenoloxidase (PPO) were identified. As identified in previous transcriptome analyses of *H. halys*, melanin formation activated by serine proteases characteristically accompanies wound clotting, phagocytosis and encapsulation of pathogens and parasites [15]. One putative nitric oxide synthase was located, indicating that a microbial defense can be mounted at the end of the signaling cascade.

**Transcription factors**

A total of 462 putative transcription factors (TFs) was identified in the *H. halys* genome, which is considerably fewer than the number found in genomes of related insects (e.g., 659 for the bed bug, *Cimex lectularius*; and 706 for the fire ant, *Solenopsis invicta*). The distribution of *H. halys* TFs across families is similar to that of other insects (Additional file 1: Figure S12). Of the 462 *H. halys* TFs, motifs could be inferred for 154 (33.3%) (see Additional file 3), based primarily on *D. melanogaster* DNA binding specificity data (95 TFs), but in addition to data from species as unrelated as human (45 TFs). Many of the largest TF families have inferred motifs for a substantial proportion of their members, including Homeodomain (40 of 53, 75%), bHLH (24 of 30, 80%), and nuclear receptors (8 of 13, 62%). As expected, the largest gap is for C2H2 zinc fingers (only 26 of 145, ~18%), which rapidly evolve by means of zinc finger array shuffling, yielding a highly diverse assortment of DBD sequences across organisms [68].

*Associated Materials and Methods:* Likely transcription factors (TFs) were identified by scanning the amino acid sequences of predicted protein coding genes for putative DNA binding domains (DBDs) and when possible, the DNA binding specificity of each TF was predicted using the procedures described in Weirauch et al. (2014) [69]. Briefly, all protein sequences were scanned for putative DBDs using the 81 Pfam [70] models listed in Weirauch and Hughes (2011) [71] and the HMMER tool [72], with the recommended detection thresholds of per-sequence E-value < 0.01 and per-domain conditional E-value < 0.01. Each protein was classified into a family based on its DBDs and their order in the protein sequence (e.g., bZIPx1, AP2x2, Homeodomain+Pou). We then aligned the resulting DBD sequences within each family using clustalOmega [73] with default settings. For protein pairs with multiple DBDs, each DBD was aligned separately. From these alignments, we calculated the sequence identity of all DBD sequence pairs (i.e., the percent of amino acid residues that are identical across all positions in the alignment). Using previously established sequence identify thresholds for each family [69], we mapped the predicted DNA binding specificities by simple transfer. For example, the DBD of HHAL006554-PA is 97% identical to the *Drosophila melanogaster* Ftz-F1 protein. As the DNA binding specificity of Ftz-F1 has already been experimentally determined and the cutoff for the nuclear receptor family of TFs is 70%, we can reasonably predict that HHAL006554-PA may have similar binding specificity as that of Ftz-F1.

**Segmentation genes**

A set of genes controlling early embryonic patterning was identified in massive genetic screens in the model insect, *D. melanogaster* [74]. This screen revealed that following establishment of the basic body axes by maternal genes, four classes of segmentation genes—maternal, gap, pair-rule and segment polarity genes—sequentially subdivide the embryo into repeated metameric units along the anterior-posterior (A/P) axis. Segment identity is then specified by a set of homeotic (*Hox*) genes, which assign different identities to specific segments (or groups of segments) along the A/P-axis [75]. Most of the genes in this regulatory hierarchy encode transcription factors that are highly conserved in insects, arthropods and, in the case of *Hox* genes, metazoans more broadly [76]. Despite conservation of gene sequences, the expression and function of many of these have diverged during evolution [77–79].

In *D. melanogaster*, maternal transcription factors organize the A/P and dorsal-ventral axes of the egg [80,81]. Orthologs of four *D. melanogaster* maternal genes were identified in the *H. halys* genome. Homologs of the *bicoid (bcd)* gene have not been isolated outside of the cyclorrhaphan dipterans and, in keeping with this, no *bcd*-like gene was found in the *H. halys* genome. It is thought that in most insect species, the function of *D.mel-bcd* in patterning the anterior region of the embryo is carried out by other genes, such as *orthodenticle (otd)* and/or *hunchback (hb)* [82]. Putative orthologs of both these genes were found in the *H. halys* genome (*otd*, NW_014466480.1:137302-406322, 55% identity to *Dmel-otd;* see below for *hb)*. *Nanos* (*nos*) is required for posterior patterning in *D. melanogaster* and its sequence is characterized by two essential CCHC motifs [83]. No match was found for *nos*, using *D. melanogaster* or *T. castaneum* *nos* nucleotide or amino acid sequence as a Blat query to search the *H. halys* genome directly. However, searches of the Transcriptome Shotgun Assembly (TSA) database using *D. melanogaster* *nos* amino acid sequence as query identified three highly significant matches: *H. halys* bmsbwbcontig71241 transcribed RNA sequence, Hhalys_USDA-ARS_IIBBL.73928 transcribed RNA sequence and *H. halys* Hhalys_USDA-ARS_IIBBL.73927 transcribed RNA sequence. Queries of the *H. halys* genome with these three sequences identified overlapping matches to a single gene model in NW_014467729.1 (E-value < e-10). This gene was annotated as *Hhal-nanos*. Alignment with *nos* genes from other species shows that they share the two characteristic CCHC domains (Additional file 1: Figure S13). Three other related genes were identified in the *H. halys* genome: *caudal* (*cad*), *Hhal-cad* (NW_014466574.1) shares 73% identity with that of *Dmel-cad*, *Hhal-dl* (NW_014466673.1) shares 61% identity with that of *Dmel-dl*, and *Hhal-cact* (NW_014466673.1) shares 45% identity with that of *Dmel-cact.*

Gap genes *Giant (Gt), Hunchback (Hb), Kruppel (Kr), knirps (kni)* and *tailless (tll)*, are the first zygotically-expressed segmentation genes in *D. melanogaster* (reviewed in [84,85]). The gap gene *kni* and its paralog, *knirps-like* (*knrl*), are the result of a duplication event in the lineage leading to insects, while duplication of *knrl* to produce *kni* appears to have occurred in brachyceran flies, which includes *D. melanogaster* [86]. Although two copies of *kni* were observed the *O. fasciatus* genome sequence and this gene had also been experimentally observed in that species [5,87], we were unable to identify *kni* in the *H. halys* genome or transcriptome. However, since the current *H. halys* genome is still divided among many scaffolds, this gene’s absence is not yet certain. Likely orthologs of four gap genes—*Gt, Hb, Kr* and *tll*—were identified in the *H. halys* genome*. Hhal-Gt* (NW_014466890.1) shares 46% identity with that of *Tcas-Gt*, *Hhal-Hb* (NW_014467650.1) shares 62% identity with that of *Dmel-Hb*, *Hhal-Kr* (NW_014467102.1) shares 68% identity with that of *Dmel-Kr*, and *Hhal-tll* (NW_014467252.1) shares 77% identity with that of *Dmel-tll.* All of these genome annotations were supported by RNA-Seq data [15,19].

The class of pair-rule genes is of particular interest because they are the first to exhibit periodic expression patterns and function in *D. melanogaster*, pre-figuring and directing the formation of body segments. Inquiries into their roles in species outside of *D. melanogaster* have revealed variable degrees of functional conservation. Thus, study of these genes in *H. halys* is of great interest due to its phylogenetic position as an outgroup of the Holometabola. In the *H. halys* genome, full sequences of all nine pair-rule gene orthologs were identified. These are: *fushi tarazu* (*ftz), even-skipped (eve), odd-skipped (odd), paired (prd), runt (run), sloppy paired (slp), odd paired (opa), hairy (h)* and *ftz transcription factor 1 (ftz-f1)* (Additional file 1: Figure S14)*.* Furthermore, RNA-Seq reads [15,19] suggest expression of all orthologs.

The gene *Hhal-eve* had previously been isolated by degenerate PCR and RACE [66]. A sequence nearly identical to this cloned sequence was found on NW_014466434.1 in the *H. halys* genome, showing that this gene spans a total of three exons. A putative *paired (prd)* ortholog was found on NW_014466504.1 including a total of eight exons. Its close relative *gooseberry (gsb)* was identified on NW_014467952.1; presence of a *gsb*-specific octapeptide motif between the Prd domain and the homeodomain helped to distinguish the paralogs [88,89]. The deduced domain of *Hhal-prd* is 98% identical to that of *Ofas-prd*, 90% to that of *Tcas-prd* and 80% to that of *Dmel-prd.*

Four Runt domain-containing genes (*runt, lozenge, RunxA* and *RunxB*) are highly conserved in insect species and there is evidence that linkage between them and direction of transcription is likewise conserved across the Insecta [90]. Four genes with Runt domains were identified in the *H. halys* genome, and two of these were found on scaffold NW_014466420.1 (Additional file 1: Figure S14). *runt* and *RunxA* have been found on the same scaffolds in the hemipterans *O. fasciatus* and *A. pisum*, suggesting conserved linkage between at least these two genes within Hemiptera. However, the two genes found 162 kb apart on NW_014466420.1 are likely *runt* and *lozenge*, as determined by reciprocal BLAST. Their shared direction of transcription provides further support for this determination, as *runt* and *lozenge* have been found to have the same direction of transcription in a diverse collection of insect lineages examined by Duncan et al. (2008) [90].

Three *odd-skipped*-related genes (*odd, sob,* and *bowl*) are known in *D. melanogaster* and have likewise been found in the *O. fasciatus* genome [5,91]. In the *H. halys* genome, four *odd*-related genes were identified. Sequence motifs specific to *sob* and *bowl* sequences were used to identify these and designations were confirmed by reciprocal BLAST. About 111 kb apart from *Hhal*-*sob* on NW_014467343.1 lie the two remaining *odd*-related genes. These are separated by about 32 kb and have remarkably similar genomic structures, appearing to be two copies of *odd-skipped*, representing a potentially recent duplication of this gene not found in the hemipteran *O. fasciatus* genome. While both *odd* genes encode four tandem zinc fingers, both *Hhal-sob* and *–bowl* encode an extra zinc finger, similar to their *D. melanogaster* orthologs. An alignment of the four zinc fingers common to all four gene products shows that while *Hhal-*Odd1 shares about 95% amino acid identity with *Hhal-*Sob and -Bowl, *Hhal-*Odd2 shares only 82% identity, suggesting that *Hhal-*Odd2 is under less pressure to maintain *odd-*like functionality (Additional file 1: Figure S15).

The 5’ region of a putative *odd paired (opa)* ortholog was found on NW_014466841.1, and the remaining sequence was found on NW_014467020.1, suggesting that these two scaffolds could be merged. A possible *Hhal-slp* ortholog was found on NW_014466429.1, with the entire coding region present in one exon. Ftz-F1 is a nuclear receptor that functions as a pair-rule gene in several holometabolous insects [92,93]. As also described in the section on NRs, *Hh-ftz-f1* was identified in the *H. halys* transcriptome and genome, where it maps to NW_014467384.1. Finally, a putative *Hhal-h* ortholog was identified on NW_014467764.1 spanning 3 exons; the Orange domain encoded by this sequence is 82% identical to that encoded by *Ofas-h* and 50% to that encoded by *Dmel-h*.

*D. melanogaster* segment polarity mutations result in deletions and duplications in each segment along the anterior-posterior axis of an embryo. These genes encode transcription factors and signaling proteins, several of which are founding members of the evolutionarily-conserved Wnt and Hedgehog signaling pathways [85]. Blat searches with *D. melanogaster* orthologs identified *H. halys* segment polarity genes, allowing the following genes to be annotated: *wingless* (*wg*) (NW_014466976.1, 79% identity to *Dmel-wg*), *hedgehog* (*hh*) (NW_014466759.1, 52% identity to *Dmel-hh*), *engrailed* *(en*) (NW_014466533.1, 76% identity to *Dmel-en*) and *invected* (*inv*) (NW_014466488.1, 68% identity to *Dmel-inv*). *Drosophila* Armadillo transduces signals from the Wingless/Wnt family and mediates intercellular adhesion in conjunction with cadherins [94]. Two *arm*-like genes were identified in the *H. halys* genome 7.7 kb apart on NW_014467042.1, with 86% and 87% identity to *Dmel-arm*. As the sequences are fairly similar to each other and to *Dmel-arm*, both encoding several armadillo β-catenin-like repeats, they may have resulted from a gene duplication event. All segment polarity gene annotations are supported by independent RNA-Seq data [15,19]. Although the sequences may not be full-length, they include conserved domains, and the amino acid sequence of each completed model matched BLAST hits for the respective predicted protein in *C. lectularius*.

The *Wnt* gene family has undergone extensive gene duplication and can be subdivided into thirteen subfamilies [95]. In insects, *wingless*, which is a classic segment polarity gene, is a *Wnt-1* subfamily ortholog*.* Wnt family members possess conserved cysteine residues that form disulfide bonds [96]. Alignment of this region of the putative *Hhal-*Wg with that of other insect species revealed that the sequence is well-conserved at both the N- and C-terminals, which contain the cysteines (Additional file 1: Figure S16).

Many phylogenetically diverse hexapod orders have species that possess two *engrailed*-family paralogs, *en* and *inv* [97]. In *Drosophila*, *en* and *inv* form a gene complex and encode homeodomain proteins that are co-regulated during development [98,99]. Both genes were found in the *H. halys* genome and transcriptomes. *Hhal-en* and -*inv* are predicted to encode the conserved, Engrailed-like homeodomain, lending additional evidence that the gene duplication that gave rise to *Dmel-en* and -*inv* predated the radiation of hexapods. As with other species, only the *inv* paralogue encodes an arginine-serine dipeptide (RS) motif (Additional file 1: Figure S17), which is part of a serine-rich domain [97]. The position and orientation of *Hh*-*en* and -*inv* relative to each other are unknown, since these map to different scaffolds (NW_014466533.1 and NW_014466488.1).

**Homeodomain gene cluster structure and synteny**

The final step in segment specification is the action of the *Hox* genes that confer unique segment identities. The Hox and Iroquois Complex (Iro-C) gene clusters encode highly conserved homeodomain transcription factors with essential roles in development, with Hox genes chiefly responsible for embryonic patterning of the anterior-posterior body axis. The Hox cluster is conserved across the Bilateria [100], and the Iro-C is found throughout the Insecta [101–103]. Annotation of the genes in these clusters provides an indicator of draft genome quality. In *H. halys* we could construct single-copy gene models for all expected orthologs. In terms of synteny, partial Hox cluster linkage was obtained across three genomic scaffolds, and linkage of the Iro-C gene pair was also confirmed.

Complete models for all ten Hox cluster genes were identified and annotated, with partial linkage within the anterior-central/3′ and posterior/5′ regions of the cluster (Additional file 1: Figure S4, panel A and Table S6). Moreover, the relative transcriptional orientation among the linked genes is conserved compared to the bilaterian Hox archetype and is also observed in the fellow hemipteran *C. lectularius*, in which the complete Hox cluster was fully assembled [101]. Assuming direct concatenation of the three Hox gene containing scaffolds, the *H. halys* Hox cluster would be 5.11 Mb, or 0.46% of the assembly. The size of the *H. halys* Hox cluster relative to the assembly accords well with that of other insects, where 0.41% is the median size across the flour beetle *T. castaneum* and four other species representing the Hemiptera and Coleoptera (*C. lectularius* [101], *Anoplophora glabripennis* [104], *O. fasciatus* [5] and *L. decemlineata* [105]).

On the other hand, the absolute size of the *H. halys* cluster is rather large compared to the other hemipterans, and is over seven-fold larger than that of the coleopteran *T. castaneum*. Thus, whereas the highly compact Hox cluster of *T. castaneum* has no invading non-Hox genes [106], a large number of other genes are interspersed within the *H. halys* Hox cluster in the current assembly. Although this pattern is similar to that observed in other insects, the identities of the non-Hox constituents are not conserved across species. As six of the ten Hox genes are located on scaffold NW_014466431.1, this was chosen for detailed manual curation assessment. Altogether, 16 gene models were curated for 14 distinct, unrelated non-Hox genes, where seven of those genes are within the posterior part of the Hox cluster. These non-Hox genes encode a disparate set of unrelated proteins, including an NADH-ubiquinone oxidoreductase subunit, an E3 ubiquitin-protein ligase, a heat shock protein, and a nuclear ribonucleoprotein. Notably, each of these genes is supported by an existing GenBank protein accession.

For the Iroquois Complex (Iro-C), clear, single-copy orthologs of both *iroquois* and *mirror* are linked in the current assembly, with two alternative isoforms supported for the former (Additional file 1: Figure S4, panel B and Table S6). The 236-kb intergenic distance between the two Iro-C genes is also slightly larger than that of the other species analyzed. However, in common with more compact Iro-C clusters, it appears likely that there are no other intervening protein-coding genes within the *H. halys* Iro-C, based on MAKER automatic gene prediction and BLASTx_Arthropoda homology data.

*A conserved set of insect nuclear receptors:*

Nuclear receptors (NRs) are a family of transcription factors defined by a modular structure including two particularly notable functional domains: the DNA binding domain (DBD) and the ligand binding domain (LBD). The presence of these two domains in a single protein allows small molecules that bind the LBD to rapidly alter transcription via the DBD in response to diet, hormones, and external environmental cues. Although many NRs have not been matched to a ligand, and are thus classified as “orphan” receptors, this does not limit their ability to influence transcriptional cascades. Both liganded and orphan NRs play diverse roles in insect physiology and development. They are especially known for controlling developmental transitions during embryogenesis, molting, and metamorphosis (reviewed in [107,108]). For example, many of the genes involved in ecdysteroid signaling, including the ecdysone receptor and its binding partners, are NRs [109]. In addition, NRs also control development, regulate homeostasis and respond to environmental cues. Because NR genes both respond to external stimuli and control insect life-cycles, they are of special interest as targets for pest management [110].

*H. halys* NR sequences were identified by reciprocal BLAST against the *D. melanogaster* ortholog of each gene, with the exception of *NR2E6*, which used the *A. mellifera* sequence as a query. The ancestral insect repertoire of true NRs, defined as those containing both the LBD and DBD, as opposed to just one or the other, is estimated to be nineteen genes [111]. Eighteen NR orthologs were annotated in the *H. halys* genome. Additional file 1: Table S7 summarizes the names, scaffolds, RNA-Seq support, and information about gene structure for each of these. All *H. halys* sequences appear to be full length, as they often include predicted 5’ and 3’ UTR. These annotations are supported by two independent transcriptome data sets in most cases, but all are supported by at least one of the two published, whole-insect transcriptomes [15,19]. The missing NR, *HR83*, was also found to be absent in another hemipteran insect genome, the milkweed bug, *O. fasciatus* [5], suggesting that these genes were actually lost (as opposed to reflecting incomplete genome coverage and assembly). *HR96*, which was absent in the pea aphid genome (*A. pisum*), was discovered and annotated in *H. halys*, while inversely, the pea aphid does have a copy of *HR83*. *HR83* may be partially redundant with another NR, as it is also absent from the genomes of several unrelated insect species, including the silkmoth, *B. mori*.

*NR2E6* was originally discovered in the honeybee genome (*A. mellifera*) and subsequently detected in the flour beetle as well (*T. casteneum*) [112,113]. Because it was not found in the pea aphid, it was uncertain whether *NR2E6* had been lost from the base of the hemipteran lineage, or only in the specific lineage leading to the *Acyrthosiphon* genus [111,114]*.* Since that time, many hemipteran genomes have been sequenced, but their *NR2E6* orthologs have been improperly named “*NR2E1”*. Phylogenetic analysis (Additional file 1: Figure S18) demonstrates that while some hemipteran genes annotated as *NR2E1* group closely with *tailless* (*NR2E2*), many form a clade with *NR2E6*, including the *H. halys* ortholog. Therefore, this gene has been annotated as *NR2E6*, not *NR2E1,* in the *H. halys* genome.

Together, these data suggest that the common ancestor of living hemipteran insects had all nineteen ancestral NR genes. After modern hemipteran lineages diverged and diversified, NR genes were lost independently. This fits well with previous observations that the cohort of NRs has been fairly stable within the insect lineage [107].

**Y chromosome gene discovery and initial analysis**

Twenty-four candidate Y-linked genes were identified from the RNA-Seq data of mixed-stage samples (see below) using the previously described chromosome quotient (CQ) method [115] (Additional file 1: Table S8). The list includes dynein heavy chain genes homologous to male fertility factors found on the Y chromosome of *D. melanogaster* [116] and a few genes homologous to cilia- and flagella-associated proteins. Also included are homologs to a putative transcriptional mediator, two genes involved in signal transduction (rho GTPase-activating protein and a kinase) and an ankyrin repeat domain-containing protein previously found on the Y chromosome of *Anopheles* mosquitoes [115]. Further verification of Y-linkage, detailed transcriptional profiling and functional analysis by knockout and ectopic expression will help identify important Y genes including the male-determining factor (M factor).

Stringent parameters were used for the discovery of Y-linked gene candidates. Y-linked genes that are not expressed or lowly expressed in the mixed RNA-Seq samples and Y-linked genes that have nearly identical X or autosomal paralogs may have been missed. On the other hand, false positives could result from differential sampling of the haplotypes in the two sexes or contamination from microbial sources. Thus, experimental verification and functional analysis are needed. For example, sex-specific PCR amplification could be used to verify the Y-linkage, and digital droplet PCR may be employed to demonstrate haploid copy number of the Y-linked sequences [117].

*Associated Materials and Methods:* RNA-Seq data from mixed stage samples—SRR1301984 (embryos and 1^st^ through 5^th^ instar nymphs) and SRR1301985 (active male and female adults, and male and female adults in diapause); see [19] for details—were used to obtain a Trinity transcript assembly using default parameters [24]. These transcripts were used as the reference sequences to compare with female Illumina gDNA reads (SRR1298382) and male Illumina gDNA reads (SRR1293900) to calculate their CQ values [115]. One hundred fifty-three transcripts (see Additional file 7) that had sufficient male gDNA coverage (matching at least 20 reads in the male gDNA Illumina data) and showed male-specificity or male bias (CQ < 0.1) were further compared to the non-redundant protein database at NCBI using BLASTx (E-value cut-off: 0.1). BLASTx results were manually inspected to rule out transcripts from possible microbial sources and transposons. Thirty-one transcripts from 24 candidate Y-linked genes were identified (Additional file 1: Table S8).

**Cuticle Proteins**

The arthropod cuticle has played an important role in the proliferation of arthropods in diverse habitats by providing structural support and protection from external stressors [118]. The cuticle comprises a proteinaceous matrix [118] composed of a variety of cuticle proteins (reviewed in [119]). The number and types of cuticle proteins present within the cuticle is diverse among the arthropods, with individual species containing some subset of about twelve cuticle protein families [120,121]. These families of cuticle proteins are classified by the occurrence of one or more conserved sequence motif(s). The CPR family, which typically contains the greatest number of proteins per species, is characterized by the “R&R Consensus” domain [122]. Some families, such as the CPR, CPAP1, and CPAP3 are common among most arthropods, whereas families such as CPCFC and CPLCW are restricted to certain orders and/or even smaller taxonomic groups [120,121]. The precise role of each family of cuticle proteins is still unclear; however, the understanding of the diversity of cuticle proteins is currently evolving as more arthropod genomes are sequenced and gene expression profiles are examined.

One hundred and fifty-six genes encoding for putative cuticle proteins were identified in *H. halys* by searching the genome with sequence motifs characteristic of different cuticle protein families as previously established [119]. CutProtFam-Pred [120] was employed to assign these genes to one of five families (CPR, CPAP1, CPAP3, CPF, and TWDL; Additional file 1: Table S9). The total number of cuticle protein genes identified in *H. halys* is similar to that of other hemipterans and the number of individual genes in each family is not remarkable when compared to other members of the order (Additional file 1: Table S10). As with other insects, the CPR genes (138) constituted the largest group of cuticle protein genes in the *H. halys* genome. Most of these (~76%) were arranged in clusters of 3 to 36 genes that were type-specific (Additional file 1: Table S11). Clustering of cuticle protein genes has been observed in other species [101,104,123] and may be common among arthropods. Clustering of these genes could allow for the coordinated regulation of cuticle proteins and thereby contribute to the development of insecticide resistance.

*Associated Materials and Methods:* The predicted gene set for *H. halys* (MAKER annotation gene set version 5.3) was obtained from the Baylor College of Medicine Human Genome Sequencing Center (BCM-HGSC) and was searched (BLASTp; [124]) using sequence motifs characteristic of several families of cuticle proteins [121]. Predicted cuticle proteins were further analyzed with CutProtFam-Pred, a cuticle protein prediction tool described in [120], to assign genes to specific families of cuticle proteins. To find the closest putative homolog to cuticle protein genes from *H. halys*, genes were searched against the Refseq protein sets downloaded from the National Center for Biotechnology Information (NCBI), or the official gene set for *A. pisum* (Refseq), *Cimex lectularius* (Refseq), *Diaphorina citri* (Refseq), *G. buenoi* (MAKER v. 5.3, BCM-HGSC), *O. fasciatus* (MAKER v. 5.3, BCM-HGSC), *R. prolixus* (CDC v. 3.1, downloaded from VectorBase), *A. mellifera* (Refseq), *D. melanogaster* (Refseq), *B. mori* (Refseq), *T. castaneum* (Refseq) and *P. humanus corporis* (Refseq). The protein sequence with the lowest E-value was considered the closest putative homolog.

**Aquaporins**

Aquaporins (AQPs) are important regulators of water content where they contribute to osmotic pressure regulation, hydration of saliva, digestion efficiency, *in vivo* hydration of offspring and thermal tolerance [125]. Contributions to water and milk provisioning as well as lactation was found to be characteristic of AQPs in *Glossina morsitans* [126], where expansion of DRIP/PRIP (*Drosophila* integral protein/*Pyrocoelia rufa* integral protein) genes in higher-order flies (*Musca domestica* and *G. morsitans*) was postulated as a means to increase water transport or specialization [123].

AQPs were compared from 46 hemipterans and 78 other arthropods to the *Halyomorpha halys* genome assembly and transcriptome [15]. Seven putative AQP genes were identified within the *H. halys* genome with only one non-specific AQP, one AQP1/DRIP, one AQP2/PRIP, one BIB (big brain protein), and three AQP4/AQP5/EGLPs (entomoglyceroporins). Consistently, the hemipteran aquaporins matched more closely to the *H. halys* genome and transcriptome than did those from less closely related species. Approximately half of the protein sequences for the predicted AQP gene models originated in the genome, with the other half originating in the transcriptome. All gene models were predicted as *H. halys* AQPs in NCBI, with most closely related homologs being identified in *C. lectularius* (4)*, D. melanogaster* (1)*, Lygus hesperus* (1) and *Fopius arisanus* (1)*.* The total number of identified AQPs in *H. halys* (7) is comparable to other closely related arthropods, such as *G.* *morsitans* (10), *D.* *melanogaster* (8), *L. cuprina* (7) and *Aedes spp.* (6) [125,127], with no apparent expansions in any specific AQP domain.

**References**

1. Hare EE, Johnston JS. Genome size determination using flow cytometry of propidium iodide-stained nuclei. Methods Mol Biol. 2011;772:3–12.

2. Gnerre S, Maccallum I, Przybylski D, Ribeiro FJ, Burton JN, Walker BJ, et al. High-quality draft assemblies of mammalian genomes from massively parallel sequence data. Proc Natl Acad Sci USA. 2011;108:1513–8.

3. Kriventseva EV, Kuznetsov D, Tegenfeldt F, Manni M, Dias R, Simão FA, et al. OrthoDB v10: sampling the diversity of animal, plant, fungal, protist, bacterial and viral genomes for evolutionary and functional annotations of orthologs. Nucleic Acids Res. 2019;47:D807–11.

4. Wheeler D, Redding AJ, Werren JH. Characterization of an ancient lepidopteran lateral gene transfer. PLoS ONE. 2013;8:e59262.

5. Panfilio KA, Vargas Jentzsch IM, Benoit JB, Erezyilmaz D, Suzuki Y, Colella S, et al. Molecular evolutionary trends and feeding ecology diversification in the Hemiptera, anchored by the milkweed bug genome. Genome Biology. 2019;20:64.

6. Poynton HC, Hasenbein S, Benoit JB, Sepulveda MS, Poelchau MF, Hughes DST, et al. The toxicogenome of *Hyalella azteca*: a model for sediment ecotoxicology and evolutionary toxicology. Environ Sci Technol. 2018;52:6009–22.

7. Tvedte ES, Walden KKO, McElroy KE, Werren JH, Forbes AA, Hood GR, et al. Genome of the parasitoid wasp *Diachasma alloeum*, an emerging model for ecological speciation and transitions to asexual reproduction. Genome Biol Evol. 2019;11:2767–73.

8. Laetsch DR, Blaxter ML. BlobTools: Interrogation of genome assemblies. F1000Research. 2017;6:1287.

9. Langmead B, Salzberg SL. Fast gapped-read alignment with Bowtie 2. Nat Meth. 2012;9:357–9.

10. Buchfink B, Xie C, Huson DH. Fast and sensitive protein alignment using DIAMOND. Nat Meth. 2015;12:59–60.

11. Cantarel BL, Korf I, Robb SMC, Parra G, Ross E, Moore B, et al. MAKER: an easy-to-use annotation pipeline designed for emerging model organism genomes. Genome Res. 2008;18:188–96.

12. Parra G, Bradnam K, Korf I. CEGMA: a pipeline to accurately annotate core genes in eukaryotic genomes. Bioinformatics. 2007;23:1061–7.

13. Stanke M, Diekhans M, Baertsch R, Haussler D. Using native and syntenically mapped cDNA alignments to improve de novo gene finding. Bioinformatics. 2008;24:637–44.

14. Korf I. Gene finding in novel genomes. BMC Bioinformatics. 2004;5:59.

15. Sparks ME, Shelby KS, Kuhar D, Gundersen-Rindal DE. Transcriptome of the invasive brown marmorated stink bug, *Halyomorpha halys* (Stål) (Heteroptera: Pentatomidae). PLoS ONE. 2014;9:e111646.

16. Gnomon [Internet]. Available from: https://www.ncbi.nlm.nih.gov/genome/annotation_euk/process/

17. O’Leary NA, Wright MW, Brister JR, Ciufo S, Haddad D, McVeigh R, et al. Reference sequence (RefSeq) database at NCBI: current status, taxonomic expansion, and functional annotation. Nucleic Acids Res. 2016;44:D733-745.

18. Paula DP, Togawa RC, Costa MMC, Grynberg P, Martins NF, Andow DA. Identification and expression profile of odorant-binding proteins in *Halyomorpha halys* (Hemiptera: Pentatomidae). Insect Mol Biol. 2016;25:580–94.

19. Ioannidis P, Lu Y, Kumar N, Creasy T, Daugherty S, Chibucos MC, et al. Rapid transcriptome sequencing of an invasive pest, the brown marmorated stink bug *Halyomorpha halys*. BMC Genomics. 2014;15:738.

20. Poelchau M, Childers C, Moore G, Tsavatapalli V, Evans J, Lee C-Y, et al. The i5k Workspace@NAL—enabling genomic data access, visualization and curation of arthropod genomes. Nucleic Acids Res. 2015;43:D714–9.

21. Buels R, Yao E, Diesh CM, Hayes RD, Munoz-Torres M, Helt G, et al. JBrowse: a dynamic web platform for genome visualization and analysis. Genome Biol. 2016;17:66.

22. Dunn NA, Unni DR, Diesh C, Munoz-Torres M, Harris NL, Yao E, et al. Apollo: Democratizing genome annotation. PLOS Computational Biology. 2019;15:e1006790.

23. Dobin A, Davis CA, Schlesinger F, Drenkow J, Zaleski C, Jha S, et al. STAR: ultrafast universal RNA-seq aligner. Bioinformatics. 2013;29:15–21.

24. Haas BJ, Papanicolaou A, Yassour M, Grabherr M, Blood PD, Bowden J, et al. De novo transcript sequence reconstruction from RNA-Seq: reference generation and analysis with Trinity. Nat Protoc. 2013;8:1494–512.

25. Wu TD, Watanabe CK. GMAP: a genomic mapping and alignment program for mRNA and EST sequences. Bioinformatics. 2005;21:1859–75.

26. Chen M-JM, Lin H, Chiang L-M, Childers CP, Poelchau MF. The GFF3toolkit: QC and Merge Pipeline for Genome Annotation. Methods Mol Biol. 2019;1858:75–87.

27. Wallau GL, da Rosa MT, De Ré FC, Loreto ELS. *Wolbachia* from *Drosophila incompta*: just a hitchhiker shared by *Drosophila* in the New and Old World? Insect Mol Biol. 2016;25:487–99.

28. Werren JH, Baldo L, Clark ME. *Wolbachia*: master manipulators of invertebrate biology. Nat Rev Microbiol. 2008;6:741–51.

29. Maumus F, Fiston-Lavier A-S, Quesneville H. Impact of transposable elements on insect genomes and biology. Current Opinion in Insect Science. 2015;7:30–6.

30. Flutre T, Duprat E, Feuillet C, Quesneville H. Considering transposable element diversification in *de novo* annotation approaches. PLoS ONE. 2011;6:e16526.

31. Quesneville H, Bergman CM, Andrieu O, Autard D, Nouaud D, Ashburner M, et al. Combined evidence annotation of transposable elements in genome sequences. PLoS Comput Biol. 2005;1:166–75.

32. Smit A, Hubley R. RepeatModeler [Internet]. Available from: http://www.repeatmasker.org/RepeatModeler/

33. Hubley R, Finn RD, Clements J, Eddy SR, Jones TA, Bao W, et al. The Dfam database of repetitive DNA families. Nucleic Acids Res. 2016;44:D81–9.

34. Bolger AM, Lohse M, Usadel B. Trimmomatic: a flexible trimmer for Illumina sequence data. Bioinformatics. 2014;30:2114–20.

35. wgsim [Internet]. Available from: https://github.com/lh3/wgsim

36. Miyamoto T, Slone J, Song X, Amrein H. A fructose receptor functions as a nutrient sensor in the *Drosophila* brain. Cell. 2012;151:1113–25.

37. Rytz R, Croset V, Benton R. Ionotropic receptors (IRs): chemosensory ionotropic glutamate receptors in *Drosophila* and beyond. Insect Biochem Mol Biol. 2013;43:888–97.

38. Ganguly A, Pang L, Duong V-K, Lee A, Schoniger H, Varady E, et al. A molecular and cellular context-dependent role for Ir76b in detection of amino acid taste. Cell Rep. 2017;18:737–50.

39. Ioannidis P, Simao FA, Waterhouse RM, Manni M, Seppey M, Robertson HM, et al. Genomic features of the damselfly *Calopteryx splendens* representing a sister clade to most insect orders. Genome Biol Evol. 2017;9:415–30.

40. Enjin A, Zaharieva EE, Frank DD, Mansourian S, Suh GSB, Gallio M, et al. Humidity sensing in *Drosophila*. Curr Biol. 2016;26:1352–8.

41. Knecht ZA, Silbering AF, Ni L, Klein M, Budelli G, Bell R, et al. Distinct combinations of variant ionotropic glutamate receptors mediate thermosensation and hygrosensation in *Drosophila*. Elife. 2016;5:e17879.

42. Knecht ZA, Silbering AF, Cruz J, Yang L, Croset V, Benton R, et al. Ionotropic Receptor-dependent moist and dry cells control hygrosensation in *Drosophila*. Elife. 2017;6:e26654.

43. Min S, Ai M, Shin SA, Suh GSB. Dedicated olfactory neurons mediating attraction behavior to ammonia and amines in *Drosophila*. Proc Natl Acad Sci USA. 2013;110:E1321-1329.

44. Hussain A, Zhang M, Üçpunar HK, Svensson T, Quillery E, Gompel N, et al. Ionotropic chemosensory receptors mediate the taste and smell of polyamines. PLoS Biol. 2016;14:e1002454.

45. Ai M, Min S, Grosjean Y, Leblanc C, Bell R, Benton R, et al. Acid sensing by the *Drosophila* olfactory system. Nature. 2010;468:691–5.

46. Grosjean Y, Rytz R, Farine J-P, Abuin L, Cortot J, Jefferis GSXE, et al. An olfactory receptor for food-derived odours promotes male courtship in *Drosophila*. Nature. 2011;478:236–40.

47. Gorter JA, Jagadeesh S, Gahr C, Boonekamp JJ, Levine JD, Billeter J-C. The nutritional and hedonic value of food modulate sexual receptivity in *Drosophila melanogaster* females. Sci Rep. 2016;6:19441.

48. Prieto-Godino LL, Rytz R, Bargeton B, Abuin L, Arguello JR, Peraro MD, et al. Olfactory receptor pseudo-pseudogenes. Nature. 2016;539:93–7.

49. Prieto-Godino LL, Rytz R, Cruchet S, Bargeton B, Abuin L, Silbering AF, et al. Evolution of acid-sensing olfactory circuits in drosophilids. Neuron. 2017;93:661-676.e6.

50. Croset V, Schleyer M, Arguello JR, Gerber B, Benton R. A molecular and neuronal basis for amino acid sensing in the *Drosophila* larva. Sci Rep. 2016;6:34871.

51. Koh T-W, He Z, Gorur-Shandilya S, Menuz K, Larter NK, Stewart S, et al. The *Drosophila* IR20a clade of ionotropic receptors are candidate taste and pheromone receptors. Neuron. 2014;83:850–65.

52. Stewart S, Koh T-W, Ghosh AC, Carlson JR. Candidate ionotropic taste receptors in the *Drosophila* larva. Proc Natl Acad Sci USA. 2015;112:4195–201.

53. Sánchez-Alcañiz JA, Silbering AF, Croset V, Zappia G, Sivasubramaniam AK, Abuin L, et al. An expression atlas of variant ionotropic glutamate receptors identifies a molecular basis of carbonation sensing. Nat Commun. 2018;9:4252.

54. Steiner H. Peptidoglycan recognition proteins: on and off switches for innate immunity. Immunological Reviews. 2004;198:83–96.

55. Lemaitre B, Hoffmann J. The host defense of *Drosophila melanogaster*. Annu Rev Immunol. 2007;25:697–743.

56. Gillespie JP, and, Kanost MR, Trenczek T. Biological mediators of insect immunity. Annual Review of Entomology. 1997;42:611–43.

57. Kamhawi S, Ramalho-Ortigao M, Pham VM, Kumar S, Lawyer PG, Turco SJ, et al. A role for insect galectins in parasite survival. Cell. 2004;119:329–41.

58. Cociancich S, Ghazi A, Hetru C, Hoffmann JA, Letellier L. Insect defensin, an inducible antibacterial peptide, forms voltage-dependent channels in *Micrococcus luteus*. J Biol Chem. 1993;268:19239–45.

59. Dandekar AM, Gouran H, Ibáñez AM, Uratsu SL, Agüero CB, McFarland S, et al. An engineered innate immune defense protects grapevines from Pierce disease. PNAS. 2012;109:3721–5.

60. Pardington P, Chaudhary A, Norvell M, Gupta G, Dandekar A, Gouran H, et al. Pathogen clearance by engineering of novel innate immune defense (INM3P.414). The Journal of Immunology. 2015;194:127.19-127.19.

61. Gong Y-H, Yu X-R, Shang Q-L, Shi X-Y, Gao X-W. Oral delivery mediated RNA interference of a carboxylesterase gene results in reduced resistance to organophosphorus insecticides in the cotton aphid, *Aphis gossypii* Glover. PLoS ONE. 2014;9:e102823.

62. Xu L, Duan X, Lv Y, Zhang X, Nie Z, Xie C, et al. Silencing of an aphid carboxylesterase gene by use of plant-mediated RNAi impairs *Sitobion avenae* tolerance of phoxim insecticides. Transgenic Res. 2014;23:389–96.

63. Mao Y-B, Cai W-J, Wang J-W, Hong G-J, Tao X-Y, Wang L-J, et al. Silencing a cotton bollworm P450 monooxygenase gene by plant-mediated RNAi impairs larval tolerance of gossypol. Nat Biotechnol. 2007;25:1307–13.

64. Bautista MAM, Miyata T, Miura K, Tanaka T. RNA interference-mediated knockdown of a cytochrome P450, CYP6BG1, from the diamondback moth, *Plutella xylostella*, reduces larval resistance to permethrin. Insect Biochem Mol Biol. 2009;39:38–46.

65. Ghosh SKB, Hunter WB, Park AL, Gundersen-Rindal DE. Double strand RNA delivery system for plant-sap-feeding insects. PLoS ONE. 2017;12:e0171861.

66. Lu Y, Chen M, Reding K, Pick L. Establishment of molecular genetic approaches to study gene expression and function in an invasive hemipteran, *Halyomorpha halys*. Evodevo. 2017;8:15.

67. Tetreau G, Cao X, Chen Y-R, Muthukrishnan S, Jiang H, Blissard GW, et al. Overview of chitin metabolism enzymes in *Manduca sexta*: Identification, domain organization, phylogenetic analysis and gene expression. Insect Biochem Mol Biol. 2015;62:114–26.

68. Najafabadi HS, Mnaimneh S, Schmitges FW, Garton M, Lam KN, Yang A, et al. C2H2 zinc finger proteins greatly expand the human regulatory lexicon. Nat Biotechnol. 2015;33:555–62.

69. Weirauch MT, Yang A, Albu M, Cote AG, Montenegro-Montero A, Drewe P, et al. Determination and inference of eukaryotic transcription factor sequence specificity. Cell. 2014;158:1431–43.

70. Finn RD, Mistry J, Tate J, Coggill P, Heger A, Pollington JE, et al. The Pfam protein families database. Nucleic Acids Res. 2010;38:D211-222.

71. Weirauch MT, Hughes TR. A catalogue of eukaryotic transcription factor types, their evolutionary origin, and species distribution. Subcell Biochem. 2011;52:25–73.

72. Eddy SR. A new generation of homology search tools based on probabilistic inference. Genome Inform. 2009;23:205–11.

73. Sievers F, Wilm A, Dineen D, Gibson TJ, Karplus K, Li W, et al. Fast, scalable generation of high-quality protein multiple sequence alignments using Clustal Omega. Mol Syst Biol. 2011;7:539.

74. Nüsslein-Volhard C, Wieschaus E. Mutations affecting segment number and polarity in *Drosophila*. Nature. 1980;287:795–801.

75. Lewis EB. A gene complex controlling segmentation in *Drosophila*. Nature. 1978;276:565–70.

76. Carroll SB, Grenier JK, Weatherbee SD. From DNA to diversity: molecular genetics and the evolution of animal design. 2nd ed. Malden, MA: Blackwell Science Ltd; 2005.

77. Heffer A, Shultz JW, Pick L. Surprising flexibility in a conserved *Hox* transcription factor over 550 million years of evolution. Proc Natl Acad Sci USA. 2010;107:18040–5.

78. Pick L, Heffer A. *Hox* gene evolution: multiple mechanisms contributing to evolutionary novelties. Ann N Y Acad Sci. 2012;1256:15–32.

79. Xiang J, Reding K, Heffer A, Pick L. Conservation and variation in pair-rule gene expression and function in the intermediate-germ beetle *Dermestes maculatus*. Development. 2017;144:4625–36.

80. Nüsslein-Volhard C, Frohnhöfer HG, Lehmann R. Determination of anteroposterior polarity in *Drosophila*. Science. 1987;238:1675–81.

81. St Johnston D, Nüsslein-Volhard C. The origin of pattern and polarity in the *Drosophila* embryo. Cell. 1992;68:201–19.

82. Lynch J, Desplan C. Evolution of development: beyond bicoid. Curr Biol. 2003;13:R557-559.

83. Curtis D, Treiber DK, Tao F, Zamore PD, Williamson JR, Lehmann R. A CCHC metal-binding domain in Nanos is essential for translational regulation. EMBO J. 1997;16:834–43.

84. Akam M. The molecular basis for metameric pattern in the *Drosophila* embryo. Development. 1987;101:1–22.

85. Gilbert SF. Developmental Biology. 9th ed. Sunderland, MA: Sinauer Associates; 2010.

86. Naggan Perl T, Schmid BGM, Schwirz J, Chipman AD. The evolution of the *knirps* family of transcription factors in arthropods. Mol Biol Evol. 2013;30:1348–57.

87. Ben-David J, Chipman AD. Mutual regulatory interactions of the trunk gap genes during blastoderm patterning in the hemipteran *Oncopeltus fasciatus*. Dev Biol. 2010;346:140–9.

88. Bopp D, Burri M, Baumgartner S, Frigerio G, Noll M. Conservation of a large protein domain in the segmentation gene paired and in functionally related genes of *Drosophila*. Cell. 1986;47:1033–40.

89. Keller RG, Desplan C, Rosenberg MI. Identification and characterization of *Nasonia* Pax genes. Insect Mol Biol. 2010;19 Suppl 1:109–20.

90. Duncan EJ, Wilson MJ, Smith JM, Dearden PK. Evolutionary origin and genomic organisation of runt-domain containing genes in arthropods. BMC Genomics. 2008;9:558.

91. Hart MC, Wang L, Coulter DE. Comparison of the structure and expression of *odd-skipped* and two related genes that encode a new family of zinc finger proteins in *Drosophila*. Genetics. 1996;144:171–82.

92. Heffer A, Grubbs N, Mahaffey J, Pick L. The evolving role of the orphan nuclear receptor *ftz-f1*, a pair-rule segmentation gene. Evol Dev. 2013;15:406–17.

93. Yu Y, Li W, Su K, Yussa M, Han W, Perrimon N, et al. The nuclear hormone receptor Ftz-F1 is a cofactor for the *Drosophila* homeodomain protein Ftz. Nature. 1997;385:552–5.

94. Loureiro J, Peifer M. Roles of Armadillo, a *Drosophila* catenin, during central nervous system development. Curr Biol. 1998;8:622–32.

95. Schubert M, Holland LZ. The *Wnt* gene family and the evolutionary conservation of *Wnt* expression [Internet]. Landes Bioscience; 2013 [cited 2018 Apr 27]. Available from: https://www.ncbi.nlm.nih.gov/books/NBK6212/

96. Janda CY, Waghray D, Levin AM, Thomas C, Garcia KC. Structural basis of Wnt recognition by Frizzled. Science. 2012;337:59–64.

97. Peel AD, Telford MJ, Akam M. The evolution of hexapod engrailed-family genes: evidence for conservation and concerted evolution. Proc Biol Sci. 2006;273:1733–42.

98. Cheng Y, Brunner AL, Kremer S, DeVido SK, Stefaniuk CM, Kassis JA. Co-regulation of *invected* and *engrailed* by a complex array of regulatory sequences in *Drosophila*. Dev Biol. 2014;395:131–43.

99. Gustavson E, Goldsborough AS, Ali Z, Kornberg TB. The *Drosophila* *engrailed* and *invected* genes: partners in regulation, expression and function. Genetics. 1996;142:893–906.

100. Krumlauf R. Evolution of the vertebrate *Hox* homeobox genes. Bioessays. 1992;14:245–52.

101. Benoit JB, Adelman ZN, Reinhardt K, Dolan A, Poelchau M, Jennings EC, et al. Unique features of a global human ectoparasite identified through sequencing of the bed bug genome. Nat Commun. 2016;7:10165.

102. Cavodeassi F, Modolell J, Gómez-Skarmeta JL. The Iroquois family of genes: from body building to neural patterning. Development. 2001;128:2847–55.

103. McNeill H, Yang CH, Brodsky M, Ungos J, Simon MA. *mirror* encodes a novel PBX-class homeoprotein that functions in the definition of the dorsal-ventral border in the *Drosophila* eye. Genes Dev. 1997;11:1073–82.

104. McKenna DD, Scully ED, Pauchet Y, Hoover K, Kirsch R, Geib SM, et al. Genome of the Asian longhorned beetle (*Anoplophora glabripennis*), a globally significant invasive species, reveals key functional and evolutionary innovations at the beetle-plant interface. Genome Biol. 2016;17:227.

105. Schoville SD, Chen YH, Andersson MN, Benoit JB, Bhandari A, Bowsher JH, et al. A model species for agricultural pest genomics: the genome of the Colorado potato beetle, *Leptinotarsa decemlineata* (Coleoptera: Chrysomelidae). Scientific Reports. 2018;8:1931.

106. Shippy TD, Ronshaugen M, Cande J, He J, Beeman RW, Levine M, et al. Analysis of the *Tribolium* homeotic complex: insights into mechanisms constraining insect Hox clusters. Dev Genes Evol. 2008;218:127–39.

107. Cheatle Jarvela AM, Pick L. The function and evolution of nuclear receptors in insect embryonic development. Curr Top Dev Biol. 2017;125:39–70.

108. King-Jones K, Thummel CS. Nuclear receptors--a perspective from *Drosophila*. Nat Rev Genet. 2005;6:311–23.

109. Yamanaka N, Rewitz KF, O’Connor MB. Ecdysone control of developmental transitions: lessons from *Drosophila* research. Annu Rev Entomol. 2013;58:497–516.

110. Palli SR, Hormann RE, Schlattner U, Lezzi M. Ecdysteroid Receptors and their Applications in Agriculture and Medicine. Vitamins & Hormones [Internet]. Academic Press; 2005 [cited 2017 Nov 15]. pp. 59–100. Available from: http://www.sciencedirect.com/science/article/pii/S008367290573003X

111. Bonneton F, Laudet V. 6 - Evolution of Nuclear Receptors in Insects A2 - Gilbert, Lawrence I. Insect Endocrinology [Internet]. San Diego: Academic Press; 2012 [cited 2016 Oct 11]. pp. 219–52. Available from: http://www.sciencedirect.com/science/article/pii/B9780123847492100068

112. Velarde RA, Robinson GE, Fahrbach SE. Nuclear receptors of the honey bee: annotation and expression in the adult brain. Insect Mol Biol. 2006;15:583–95.

113. Bonneton F, Chaumot A, Laudet V. Annotation of *Tribolium* nuclear receptors reveals an increase in evolutionary rate of a network controlling the ecdysone cascade. Insect Biochem Mol Biol. 2008;38:416–29.

114. Consortium TIAG. Genome Sequence of the Pea Aphid Acyrthosiphon pisum. PLOS Biology. 2010;8:e1000313.

115. Hall AB, Qi Y, Timoshevskiy V, Sharakhova MV, Sharakhov IV, Tu Z. Six novel Y chromosome genes in *Anopheles* mosquitoes discovered by independently sequencing males and females. BMC Genomics. 2013;14:273.

116. Carvalho AB, Lazzaro BP, Clark AG. Y chromosomal fertility factors *kl-2* and *kl-3* of *Drosophila melanogaster* encode dynein heavy chain polypeptides. Proc Natl Acad Sci USA. 2000;97:13239–44.

117. Hall AB, Basu S, Jiang X, Qi Y, Timoshevskiy VA, Biedler JK, et al. A male-determining factor in the mosquito *Aedes aegypti*. Science. 2015;348:1268–70.

118. Neville AC. Biology of the arthropod cuticle. New York: Springer-Verlag; 1975.

119. Willis JH, Iconomidou VA, Smith RF, Hamodrakas SJ. Cuticular proteins. Comprehensive Molecular Insect Science. Gilbert L.I., Iatrou K., Gill S.S. (Eds.). Australia: Elsevier Pergamon; 2005. pp. 79–109.

120. Ioannidou ZS, Theodoropoulou MC, Papandreou NC, Willis JH, Hamodrakas SJ. CutProtFam-Pred: detection and classification of putative structural cuticular proteins from sequence alone, based on profile hidden Markov models. Insect Biochem Mol Biol. 2014;52:51–9.

121. Willis JH. Structural cuticular proteins from arthropods: annotation, nomenclature, and sequence characteristics in the genomics era. Insect Biochem Mol Biol. 2010;40:189–204.

122. Rebers JE, Riddiford LM. Structure and expression of a *Manduca sexta* larval cuticle gene homologous to *Drosophila* cuticle genes. J Mol Biol. 1988;203:411–23.

123. Papanicolaou A, Schetelig MF, Arensburger P, Atkinson PW, Benoit JB, Bourtzis K, et al. The whole genome sequence of the Mediterranean fruit fly, *Ceratitis capitata* (Wiedemann), reveals insights into the biology and adaptive evolution of a highly invasive pest species. Genome Biol. 2016;17:192.

124. McGinnis S, Madden TL. BLAST: at the core of a powerful and diverse set of sequence analysis tools. Nucleic Acids Res. 2004;32:W20-25.

125. Anstead CA, Korhonen PK, Young ND, Hall RS, Jex AR, Murali SC, et al. *Lucilia cuprina* genome unlocks parasitic fly biology to underpin future interventions. Nat Commun. 2015;6:7344.

126. Benoit JB, Hansen IA, Attardo GM, Michalková V, Mireji PO, Bargul JL, et al. Aquaporins are critical for provision of water during lactation and intrauterine progeny hydration to maintain tsetse fly reproductive success. PLoS Negl Trop Dis. 2014;8:e2517.

127. International Glossina Genome Initiative. Genome sequence of the tsetse fly (*Glossina morsitans*): vector of African trypanosomiasis. Science. 2014;344:380–6.

128. Saha S, Hosmani PS, Villalobos-Ayala K, Miller S, Shippy T, Flores M, et al. Improved annotation of the insect vector of citrus greening disease: biocuration by a diverse genomics community. Database. 2019;2019.

129. Nei M, Gojobori T. Simple methods for estimating the numbers of synonymous and nonsynonymous nucleotide substitutions. Mol Biol Evol. 1986;3:418–26.

130. Kumar S, Stecher G, Li M, Knyaz C, Tamura K. MEGA X: molecular evolutionary genetics analysis across computing platforms. Mol Biol Evol. 2018;35:1547–9.

131. Li H, Leavengood JM, Chapman EG, Burkhardt D, Song F, Jiang P, et al. Mitochondrial phylogenomics of Hemiptera reveals adaptive innovations driving the diversification of true bugs. Proc Biol Sci. 2017;284:20171223.

132. Dereeper A, Guignon V, Blanc G, Audic S, Buffet S, Chevenet F, et al. Phylogeny.fr: robust phylogenetic analysis for the non-specialist. Nucleic Acids Res. 2008;36:W465-469.

133. Milne I, Wright F, Rowe G, Marshall DF, Husmeier D, McGuire G. TOPALi: software for automatic identification of recombinant sequences within DNA multiple alignments. Bioinformatics. 2004;20:1806–7.

134. Edgar RC. MUSCLE: multiple sequence alignment with high accuracy and high throughput. Nucleic Acids Res. 2004;32:1792–7.

**Table S1.** Sequencing, assembly, annotation statistics and accession numbers.

| Bio Projects | i5K Pilot NCBI Bio-project | PRJNA163973  http://www.ncbi.nlm.nih.gov/bioproject/163973 |
| --- | --- | --- |
|  | *Halyomorpha halys* NCBI Bio-project | PRJNA168118 https://www.ncbi.nlm.nih.gov/bioproject/168118 |
|  | NCBI Bio-sample | SAMN02737379  https://www.ncbi.nlm.nih.gov/biosample/SAMN02737379 |
|  | EMBRAPA/ UMN antennal transcriptome | PRJNA263721  https://www.ncbi.nlm.nih.gov/bioproject/263721 |
|  | USDA-ARS whole-insect transcriptome | PRJNA242849  https://www.ncbi.nlm.nih.gov/bioproject/242849 |
|  | UMD whole-insect transcriptome | PRJNA248431  https://www.ncbi.nlm.nih.gov/bioproject/248431 |
| Genome  Sequence | 180bp (192bp actual) insert, female DNA | 2 Illumina HiSeq 2000 run: 240.7M read pairs, 48.6 Gbp and  42.5M read pairs, 8.6 Gbp |
|  | 500bp (actual n/a) insert female DNA | 1 Illumina HiSeq 2000 run: 129.3M read pairs, 26.1 Gbp |
|  | 3kb (3,042 bp actual) insert female DNA | 2 Illumina HiSeq 2000 runs: 78.3M read pairs, 15.8 Gbp and  227.4M read pairs, 45.9 Gbp |
|  | 8kb (actual n/a) insert female DNA | 1 Illumina HiSeq 2000 run: 91.6M read pairs, 18.5 Gbp |
|  | 300bp (240 bp actual) insert male DNA | 1 Illumina HiSeq 2000 run: 140.7M read pairs, 28.4 Gbp |
|  | 180bp insert NCBI SRA Accession | SRX552655 and SRX552650  https://www.ncbi.nlm.nih.gov/sra/SRX552655 and  https://www.ncbi.nlm.nih.gov/sra/SRX552650 |
|  | 500bp insert NCBI SRA Accession | SRX552654  https://www.ncbi.nlm.nih.gov/sra/SRX552654 |
|  | 3kb insert NCBI SRA Accession | SRX552653 and SRX552652  https://www.ncbi.nlm.nih.gov/sra/SRX552653 and  https://www.ncbi.nlm.nih.gov/sra/SRX552652 |
|  | 8kb insert NCBI SRA Accession | SRX552651  https://www.ncbi.nlm.nih.gov/sra/SRX552651 |
|  | 300bp insert male NCBI SRA Accession | SRX548749  https://www.ncbi.nlm.nih.gov/sra/SRX548749 |
| Genome  Assembly | Number of contigs | 132,308 |
|  | Contig N50 | 17,705 bp |
|  | Number of scaffolds | 12,168 |
|  | Scaffold N50 | 802,423 bp |
|  | Size of final assembly | 1,150,108,890 bp |
|  | Size of final assembly - without gaps | 1,000,800,076 bp |
|  | NCBI Genome Assembly Accession | GCA_000696795.1  https://www.ncbi.nlm.nih.gov/assembly/GCA_000696795.1 |
| RNA-Seq  Resources  Utilized | antenna (PE50; 123,932,954 pairs) | SRX731658 (published in [18])  https://www.ncbi.nlm.nih.gov/sra/SRX731658 |
|  | tube1 (adults; PE100; 98,116,956 pairs) | SRX554889 (published in [19])  https://www.ncbi.nlm.nih.gov/sra/SRX554889 |
|  | tube2 (juveniles; PE100; 85,227,647 pairs) | SRX554890 (published in [19])  https://www.ncbi.nlm.nih.gov/sra/SRX554890 |
|  | 2nd instar (SE100; 196,439,408 reads) | SRX7502104 (published in [15])  https://www.ncbi.nlm.nih.gov/sra/SRX502104 |
|  | 4th instar (SE100; 208,313,504 reads) | SRX502746 (published in [15])  https://www.ncbi.nlm.nih.gov/sra/SRX502746 |
|  | male adult (SE100; 189,721,966 reads) | SRX502747 (published in [15])  https://www.ncbi.nlm.nih.gov/sra/SRX502747 |
|  | female adult (SE100; 164,646,253 reads) | SRX502748 (published in [15])  https://www.ncbi.nlm.nih.gov/sra/SRX502748 |
| Automated Genome Annotation (Hhal_0.5.3) | Genes (Hhal_0.5.3) | 11,374 |
|  | Average Transcript length | 1099.6 bp |
|  | Average CDS length | 1086.0 bp (362.0 aa) |
|  | Exons per gene | 5.32 |
|  | Genome Annotation Link | National Agricultural Library  https://i5k.nal.usda.gov/Halyomorpha_halys |

**Table S2.** OrthoDB v10 comparison of five species for ortholog presence and copy-number in Hemiptera-level orthogroups. *Apisu* ~ *Acyrthosiphon pisum*, *Rprol* ~ *Rhodnius prolixus*, *Clect* ~ *Cimex lectularius*, *Ofasc* ~ *Oncopeltus fasciatus*, and *Hhaly* ~ *Halyomorpha halys*.

| **Hemipteran orthogroup metrics** | **Species** | | | | |
| --- | --- | --- | --- | --- | --- |
|  | ***Apisu*** | ***Rprol*** | ***Clect*** | ***Ofasc*** | ***Hhaly*** |
| A. Orthogroups that include this species out of all orthogroups with at least four of these five species (n= 7,045 orthogroups) | 5,546  (78.7%) | 6,618  (93.9%) | 6,928  (98.3%) | 6,862  (97.4%) | 6,971  (98.9%) |
| B. Orthogroups missing an ortholog from this species where the other four species all have single-copy orthologs | 1150 | 297 | 76 | 113 | 46 |
| C. Orthogroups missing an ortholog from this species where the other four species have single- and multi-copy orthologs | 349 | 130 | 41 | 70 | 28 |
| D. Orthogroups with more than one ortholog from this species where the other four species all have single-copy orthologs | 513 | 140 | 104 | 258 | 179 |
| E. Total number of genes in orthogroups with at least four of the five species (irrespective of ortholog copy-number) | 8,900 | 7,886 | 8,352 | 8,642 | 9,295 |

**Table S3.** Scaffolds present in the *H. halys* assembly (accession GCA_000696795.1) that may originate from contaminant sources.

| Wheeler-Werren | | BlobTools | | | | | |
| --- | --- | --- | --- | --- | --- | --- | --- |
| Bacterial | | Bacterial | | Fungal | | Plant | |
| ID | Length (bp) | ID | Length (bp) | ID | Length (bp) | ID | Length (bp) |
| NW_014474542 | 4569 | NW_014468335 | 40,397 | NW_014471080 | 7059 | NW_014468385 | 45,522 |
| NW_014477531 | 4019 | NW_014470722 | 12,578 | NW_014469089 | 6467 | NW_014471191 | 11,464 |
| NW_014475298 | 1983 | NW_014470404 | 11,060 | NW_014473533 | 4480 | NW_014469261 | 11,097 |
| NW_014478343 | 1903 | NW_014471202 | 9338 | NW_014470836 | 2260 | NW_014469274 | 10,161 |
| NW_014474180 | 1751 | NW_014470298 | 7010 | NW_014474778 | 1776 | NW_014470137 | 9247 |
| NW_014476948 | 1694 | NW_014470812 | 6517 |  |  | NW_014471199 | 5621 |
| NW_014475008 | 1539 | NW_014475255 | 5813 |  |  | NW_014474693 | 5220 |
| NW_014475884 | 1476 | NW_014472042 | 5658 |  |  | NW_014475752 | 4728 |
| NW_014473859 | 1471 | NW_014471830 | 4804 |  |  | NW_014474109 | 4649 |
| NW_014477545 | 1435 | NW_014472684 | 4590 |  |  | NW_014475653 | 4460 |
| NW_014475370 | 1413 | NW_014475292 | 3974 |  |  | NW_014470977 | 4419 |
| NW_014478113 | 1403 | NW_014472895 | 3776 |  |  | NW_014474317 | 4199 |
| NW_014477325 | 1399 | NW_014473324 | 3527 |  |  | NW_014472305 | 3956 |
| NW_014477696 | 1211 | NW_014476121 | 3345 |  |  | NW_014477417 | 3944 |
| NW_014477640 | 1189 | NW_014473592 | 3113 |  |  | NW_014473411 | 3451 |
| NW_014476920 | 1169 | NW_014477350 | 1536 |  |  | NW_014474592 | 2714 |
| NW_014475900 | 1082 |  |  |  |  | NW_014475849 | 1535 |
| NW_014474486 | 1032 |  |  |  |  | NW_014477311 | 1238 |
| NW_014475046 | 1029 |  |  |  |  | NW_014477047 | 1142 |
| NW_014478211 | 999 |  |  |  |  |  |  |
| NW_014474689 | 932 |  |  |  |  |  |  |

**Table S4.** Counts of repetitive DNA elements encountered in the *H. halys* genome assembly.

| Type | Sub-type | Coverage (bp) | % assembly | % ungapped |
| --- | --- | --- | --- | --- |
| LTR retrotransposon | Copia | 202,672 | 0.02 | 0.02 |
|  | Gypsy | 12,681,665 | 1.10 | 1.27 |
|  | DIRS | 0 | 0.00 | 0.00 |
|  | BEL | 0 | 0.00 | 0.00 |
| Non-LTR retrotransposon | LINE | 124,505,321 | 10.83 | 12.44 |
|  | SINE | 1,450,481 | 0.13 | 0.14 |
| Putative_retrotransposon | Putative_RT | 3,734,625 | 0.32 | 0.37 |
| DNA | DNA | 28,236,569 | 2.46 | 2.82 |
| Helitron | Helitron | 28,523 | 0.00 | 0.00 |
| Crypton | Crypton | 0 | 0.00 | 0.00 |
| Confused | Confused | 19,073,523 | 1.66 | 1.91 |
| Unclassified | Unclassified | 91,467,719 | 7.95 | 9.14 |
| Hostgene | Hostgene | 19,515 | 0.00 | 0.00 |
| Tandem repeats | Tandem repeats | 1,246,837 | 0.11 | 0.12 |
| Virus | Endovirus | 2,094,897 | 0.18 | 0.21 |

**Table S5.** *H. halys* predicted protein products associated with the RNAi pathway.

| RISC | Dicer | XP_014270680.1 | endoribonuclease Dcr-1 |
| --- | --- | --- | --- |
|  |  | XP_014275310.1 | endoribonuclease Dicer isoform X1 |
|  |  | XP_014275311.1 | endoribonuclease Dicer isoform X2 |
|  | TARBP | XP_014270387.1 | probable methyltransferase TARBP1 isoform X1 |
|  |  | XP_014270396.1 | probable methyltransferase TARBP1 isoform X2 |
|  |  | XP_014270420.1 | probable methyltransferase TARBP1 isoform X5 |
|  |  | XP_014270429.1 | probable methyltransferase TARBP1 isoform X6 |
|  |  | XP_014270436.1 | probable methyltransferase TARBP1 isoform X7 |
|  |  | XP_014285641.1 | RISC-loading complex subunit tarbp2-like |
|  |  | XP_014285642.1 | RISC-loading complex subunit tarbp2-like |
|  |  | XP_014285643.1 | RISC-loading complex subunit tarbp2-like |
|  |  | XP_014285644.1 | RISC-loading complex subunit tarbp2-like |
|  |  | XP_014274312.1 | RISC-loading complex subunit tarbp2-like isoform X1 |
|  | Argonaute | XP_014287702.1 | protein argonaute-2 isoform X1 |
|  |  | XP_014287703.1 | protein argonaute-2 isoform X2 |
|  |  | XP_014287704.1 | protein argonaute-2 isoform X2 |
|  |  | XP_014287705.1 | protein argonaute-2 isoform X3 |
|  |  | XP_014277851.1 | protein argonaute-2-like |
|  |  | XP_014271330.1 | protein argonaute-2-like isoform X1 |
|  |  | XP_014271331.1 | protein argonaute-2-like isoform X2 |
|  |  | XP_014271332.1 | protein argonaute-2-like isoform X3 |
|  |  | XP_014276831.1 | protein argonaute-3 |
|  | Aubergine | XP_014270559.1 | protein aubergine-like |
|  |  | XP_014275924.1 | protein aubergine-like isoform X1 |
|  |  | XP_014275925.1 | protein aubergine-like isoform X2 |
|  |  | XP_014275927.1 | protein aubergine-like isoform X3 |
|  |  | XP_014275928.1 | protein aubergine-like isoform X3 |
|  |  | XP_014275929.1 | protein aubergine-like isoform X3 |
|  | fragile X mental retardation syndrome-related protein 1(FMR1) | XP_014289785.1 | cytoplasmic FMR1-interacting protein isoform X1 |
|  |  | XP_014289786.1 | cytoplasmic FMR1-interacting protein isoform X2 |
|  |  | XP_014287676.1 | fragile X mental retardation syndrome-related protein 1 isoform X1 |
|  |  | XP_014287677.1 | fragile X mental retardation syndrome-related protein 1 isoform X2 |
|  |  | XP_014287678.1 | fragile X mental retardation syndrome-related protein 1 isoform X3 |
|  |  | XP_014293048.1 | nuclear fragile X mental retardation-interacting protein 1 |
|  | Vasa Intronic Gene (VIG) | XP_014278885.1 | ATP-dependent RNA helicase vasa, isoform A |
| Helicases | DEAD/H | XP_014271232.1 | DEAD-box ATP-dependent RNA helicase 20-like isoform X1 |
|  |  | XP_014271234.1 | DEAD-box ATP-dependent RNA helicase 20-like isoform X2 |
|  |  | XP_014271235.1 | DEAD-box ATP-dependent RNA helicase 20-like isoform X3 |
|  |  | XP_014280060.1 | DEAD-box helicase Dbp80-like |
|  |  | XP_014283358.1 | SWI/SNF-related matrix-associated actin-dependent regulator of chromatin subfamily A containing DEAD/H box 1 homolog isoform X1 |
|  |  | XP_014283359.1 | SWI/SNF-related matrix-associated actin-dependent regulator of chromatin subfamily A containing DEAD/H box 1 homolog isoform X2 |
|  |  | XP_014285568.1 | DEAD-box ATP-dependent RNA helicase 40-like |
|  |  | XP_014286900.1 | DEAD-box helicase Dbp80-like |
|  | Bel | XP_014276206.1 | BEL1-like homeodomain protein 2 |
|  |  | XP_014279435.1 | ATP-dependent RNA helicase bel isoform X1 |
|  |  | XP_014279436.1 | ATP-dependent RNA helicase bel isoform X2 |
|  | Ddx | XP_014282067.1 | ATP-dependent RNA helicase Ddx1 |
|  |  | XP_014287493.1 | probable ATP-dependent RNA helicase DDX5 |
|  |  | XP_014287508.1 | probable ATP-dependent RNA helicase DDX5 |
|  |  | XP_014270548.1 | probable ATP-dependent RNA helicase DDX10 |
|  |  | XP_014273577.1 | probable ATP-dependent RNA helicase DDX11 isoform X1 |
|  |  | XP_014273578.1 | probable ATP-dependent RNA helicase DDX11 isoform X2 |
|  |  | XP_014274651.1 | probable ATP-dependent RNA helicase ddx20 |
|  |  | XP_014290252.1 | probable ATP-dependent RNA helicase DDX20 |
|  |  | XP_014293384.1 | probable ATP-dependent RNA helicase DDX23, partial |
|  |  | XP_014293893.1 | probable ATP-dependent RNA helicase DDX23, partial |
|  |  | XP_014293970.1 | probable ATP-dependent RNA helicase DDX23 |
|  |  | XP_014279138.1 | ATP-dependent RNA helicase DDX24 |
|  |  | XP_014274809.1 | probable ATP-dependent RNA helicase DDX27 |
|  |  | XP_014294719.1 | probable ATP-dependent RNA helicase DDX28 |
|  |  | XP_014274677.1 | probable ATP-dependent RNA helicase DDX31 |
|  |  | XP_014279591.1 | ATP-dependent RNA helicase DDX42 |
|  |  | XP_014284233.1 | probable ATP-dependent RNA helicase DDX46 |
|  |  | XP_014280268.1 | probable ATP-dependent RNA helicase DDX47 |
|  |  | XP_014282210.1 | probable ATP-dependent RNA helicase DDX49 |
|  |  | XP_014280134.1 | ATP-dependent RNA helicase DDX51 |
|  |  | XP_014285861.1 | probable ATP-dependent RNA helicase DDX52 |
|  |  | XP_014270929.1 | ATP-dependent RNA helicase DDX54 |
|  |  | XP_014285587.1 | ATP-dependent RNA helicase DDX55 |
|  |  | XP_014287185.1 | probable ATP-dependent RNA helicase DDX56, partial |
|  | Armitage (Armi) | XP_014272862.1 | probable RNA helicase armi |
|  |  | XP_014272863.1 | probable RNA helicase armi |
|  |  | XP_014289817.1 | probable RNA helicase armi |

**Table S6.** Positional information for the annotated homeobox genes. RNA-Seq expression data support alternate isoforms of *Hhal-pb/Hox2* and *Hhal-iro* that differ in the 5′ UTR region only, with alternate first exons for *Hhal-pb* and an optional additional first exon in the case of *Hhal-iro*.

| **Gene** | **Locus: start-end (DNA strand)** | **Locus length (nt)** | **Protein length (aa)** | **Number of CDS exons** |
| --- | --- | --- | --- | --- |
| **Hox cluster** | | | | |
| *Labial* | NW_014468139.1:19901-98297 (+) | 78,397 | 251 | 2 |
| *proboscipedia-RA* | NW_014467139.1:786122-948572 (+) | 162,451 | 586 | 3 |
| *proboscipedia-RB* | NW_014467139.1:804856-948572 (+) | 143,717 | 586 | 3 |
| *Zerknüllt* | NW_014467139.1:605311-669522 (+) | 64,212 | 185 | 3 |
| *Deformed* | NW_014467139.1:345875-417852 (+) | 71,978 | 307 | 2 |
| *Sex combs reduced* | NW_014466431.1:840165-933131 (-) | 92,967 | 299 | 2 |
| *fushi tarazu* | NW_014466431.1:995793-1001303 (-) | 5,511 | 290 | 2 |
| *Antennapedia* | NW_014466431.1:1352828-1476048 (-) | 123,221 | 291 | 2 |
| *Ultrabithorax* | NW_014466431.1:1915921-2368063 (-) | 452,143 | 289 | 2 |
| *abdominal-A* | NW_014466431.1:2842685-2943084 (-) | 100,400 | 339 | 3 |
| *Abdominal-B* | NW_014466431.1:3627632-3815551 (-) | 187,920 | 314 | 2 |
| **Iroquois Complex (Iro-C) cluster** | | | | |
| *iroquois-RA* | NW_014467512.1:334010-469039 (-) | 135,030 | 400 | 6 |
| *iroquois-RB* | NW_014467512.1:334010-560703 (-) | 226,694 | 400 | 6 |
| *mirror* | NW_014467512.1:20648-98234 (-) | 77,587 | 363 | 5 |

**Table S7.** Nuclear receptors of *H. halys*.

| Nuclear receptor | NRNC Nomenclature | Genomic Coordinates | Exon Count | Protein Length |
| --- | --- | --- | --- | --- |
| E75 | NR1D3 | NW_014466721.1:1324326-1340882 | 6 | 687 |
| E78 | NR1E1 | N term: NW_014468144.1:43715..43890 C term: NW_014468147.1:338-34797 | 6 | 338 |
| HR3 | NR1F4 | NW_014466772.1:192272-301820 | 14 | 552 |
| EcR | NR1H1 | NW_014466982.1:263903-387490 | 8 | 454 |
| HR96 | NR1J1 | NW_014466474.1:3168506-3176043 | 7 | 313 |
| HNF4 | NR2A4 | NW_014467008.1:52075-96106 | 9 | 409 |
| USP | NR2B4 | NW_014466441.1:396661-503867 | 9 | 434 |
| HR78 | NR2D1 | NW_014466731.1:556453-577461 | 10 | 492 |
| Tailless | NR2E2 | NW_014467252.1:197997-221073 | 8 | 347 |
| Dissatisfaction | NR2E4 | NW_014466721.1:440668-477852 | 9 | 345 |
| HR51 | NR2E3 | NW_014466770.1:110531-228810 | 8 | 332 |
| NR2E6 | NR2E6 | NW_014467853.1:135448-163270 | 9 | 337 |
| Seven up | NR2F3 | NW_014467660.1:71353-171535 | 3 | 251 |
| ERR | NR3B1 | NW_014466629.1:1077415-1100546 | 9 | 438 |
| HR38 | NR4A4 | NW_014467821.1:265771-295410 | 9 | 591 |
| FTZ-F1 | NR5A3 | NW_014467384.1:143322-490395 | 7 | 542 |
| HR39 | NR5B1 | NW_014467999.1:103637-281366 | 15 | 672 |
| HR4 | NR6A2 | NW_014467359.1:511969-632903 | 13 | 641 |

**Table S8.** Listing of candidate Y-linked genes. Sequences for assembled transcripts are available in Additional file 7. The top-scoring Gnomon model (E-value not more than 1e-15) is provided, as well as a functional description of each gene per best match with any protein from NCBI NR (see Methods).

| Assembled Transcript Gene ID | Best Gnomon model match | Gene Description (per comparison with NCBI NR) |
| --- | --- | --- |
| TRINITY_DN120296_c2 | none | mediator of RNA polymerase II |
| TRINITY_DN120978_c0 | XP_014271137.1 | rho GTPase-activating protein conundrum |
| TRINITY_DN109752_c0 | none | uncharacterized, adenylate kinase 9-like |
| TRINITY_DN119642_c0 | XP_014292262.1 | ankyrin repeat domain-containing, Ser/Thr-protein phosphatase |
| TRINITY_DN110542_c0 | XP_014292274.1 | dynein heavy chain |
| TRINITY_DN119625_c0 | XP_014292274.1 | dynein heavy chain |
| TRINITY_DN120259_c5 | XP_014290614.1 | dynein heavy chain 10 |
| TRINITY_DN120350_c0 | XP_014292274.1 | dynein heavy chain |
| TRINITY_DN90170_c0 | none | dynein heavy chain |
| TRINITY_DN62894_c0 | XP_014292274.1 | dynein heavy chain |
| TRINITY_DN92980_c0 | XP_014292274.1 | dynein heavy chain |
| TRINITY_DN118953_c4 | XP_014285361.1 | cilia- and flagella-associated protein 57 |
| TRINITY_DN118788_c0 | none | cilia- and flagella-associated protein 45 |
| TRINITY_DN115471_c0 | XP_014285361.1 | cilia- and flagella-associated protein 57 |
| TRINITY_DN120021_c1 | none | glycine-rich cell wall structural protein  (cilia- and flagella-associated, spidroin-1-like ) |
| TRINITY_DN116991_c1 | none | glycine-rich cell wall structural protein |
| TRINITY_DN121493_c0 | XP_014282429.1 | maltase A1-like |
| TRINITY_DN119394_c2 | XP_014282109.1 | cathepsin L1-like |
| TRINITY_DN117577_c1 | XP_014276388.1 | uncharacterized protein, cuticle protein 1-like |
| TRINITY_DN117068_c0 | none | cuticle protein 19-like |
| TRINITY_DN121573_c2 | none | peptidyl-prolyl cis-trans isomerase sig-7 |
| TRINITY_DN119896_c1 | XP_014279566.1 | uncharacterized protein |
| TRINITY_DN17690_c0 | XP_014289788.1 | uncharacterized protein |
| TRINITY_DN123483_c0 | none | histone-lysine N-methyltransferase SETMAR-like |

| **Table S9.** Number of genes identified as putative cuticle proteins per family in the genome of *H. halys*. | | | | | | | | |
| --- | --- | --- | --- | --- | --- | --- | --- | --- |
| CPR^a^ | | |  |  |  | |  |  |
| RR-1 | RR-2 | Uncl | CPAP1 | CPAP3 | | CPF | TWDL | Total |
| 27 | 106 | 5 | 8 | 3 | 4 | | 3 | 156 |
| ^a^Sequences that scored above the assigned cutoffs for the RR-1 and RR-2 models were classified as the corresponding type, whereas sequences with scores below the assigned cutoffs but above 0 were characterized as “unclassified” (Uncl). For more information, see Ioannidou et al. (2014) [120]. | | | | | | | | |

| **Table S10.** Number of genes identified as putative cuticle proteins per species in the genomes of several insect orders. | | | | | | | | | | | |
| --- | --- | --- | --- | --- | --- | --- | --- | --- | --- | --- | --- |
| **Order** | **Species** | **CPR** | **CPAP1** | **CPAP3** | **CPCFC** | **CPF** | **CPLCA** | **CPLCG** | **CPLCP** | **TWDL** | **Total** |
| Hemiptera | *Acyrthosiphon pisum* ^b^ | 117 | 11 | 10 | 1 | 3 | 0 | 0 | 0 | 3 | 145 |
|  | *Cimex lectularius* ^c^ | 121 | 15 | 6 | 0 | 5 | 0 | 0 | 0 | 3 | 149 |
|  | *Diaphorina citri* ^d^ | 38 | 11 | 8 | 0 | 3 | 0 | 0 | 0 | 3 | 63 |
|  | *Gerris buenoi* ^a^ | 126 | 10 | 6 | 0 | 3 | 0 | 0 | 0 | 10 | 155 |
|  | ***Halyomorpha halys* ^a^** | **138** | **8** | **3** | **0** | **4** | **0** | **0** | **0** | **3** | **156** |
|  | *Oncopeltus fasciatus* ^a^ | 133 | 11 | 7 | 0 | 7 | 0 | 0 | 0 | 3 | 173 |
|  | *Rhodnius prolixus* ^a^ | 91 | 10 | 6 | 1 | 2 | 0 | 0 | 0 | 1 | 111 |
| Hymenoptera | *Apis* *mellifera* ^b^ | 38 | 15 | 7 | 0 | 4 | 0 | 0 | 0 | 2 | 66 |
| Diptera | *Drosophila melanogaster* ^b^ | 137 | 29 | 10 | 1 | 5 | 13 | 4 | 0 | 29 | 228 |
| Lepidoptera | *Bombyx mori* ^b^ | 144 | 13 | 6 | 1 | 1 | 2 | 0 | 0 | 4 | 171 |
| Coleoptera | *Tribolium castaneum* ^b^ | 110 | 13 | 7 | 2 | 5 | 1 | 1 | 0 | 3 | 142 |
| Phthiraptera | *Pediculus humanus* ^b^ | 41 | 12 | 6 | 1 | 1 | 0 | 0 | 0 | 2 | 63 |
| ^a^ Cuticle protein numbers determined by analyzing gene sets with CutProtFam-Pred [120].  ^b^ Cuticle protein numbers determined from Ioannidou et al. (2014) [120].  ^c^ Cuticle protein numbers determined from Benoit et al. (2016) [101].  ^d^ Cuticle protein numbers determined from Saha et al. (2019) [128]. | | | | | | | | | | | |

| **Table S11.** Clusters of genes coding for cuticle proteins in the genome of *H. halys*. | | | | |
| --- | --- | --- | --- | --- |
| **Scaffold #** | **# Genes** | **Family** | **Length (Kbp)** | **Density (Kbp/gene)** |
| NW_014466874.1 | 36 | CPR RR-2 | 398 | 11.0 |
| NW_014466523.1 | 33 | CPR RR-2 | 324 | 9.8 |
| NW_014466824.1 | 14 | CPR RR-1 | 903 | 64.5 |
| NW_014466436.1 | 9 | CPR RR-2 | 69 | 7.6 |
| NW_014466518.1 | 8 | CPR RR-2/CPR Uncl | 99 | 12.4 |
| NW_014466563.1 | 6 | CPR RR-2 | 69 | 11.5 |
| NW_014468619.1 | 5 | CPR RR-1 | 100 | 19.9 |
| NW_014466547.1 | 4 | CPF | 306 | 76.6 |
| NW_014467379.1 | 3 | CPR RR-1 | 126 | 41.8 |

**Table S12.** Odorant-binding protein genes and pseudogenes (Ψ) annotated in the genome of *H. halys*. The annotation of the *obp21* gene is incomplete because some exons were missing, likely due to incomplete DNA sequencing/assembling. The gene names are not indicative of proximity.

| **Gene** | **Models used for annotation** | **Scaffold ID, locus and orientation** | **Length (bp)** | **Exons** | **qPCR support** |
| --- | --- | --- | --- | --- | --- |
| Odorant-binding protein 1 | HhalOBP1, 516 bp, 171 aa, GenBank KT875737 | NW_014467362.1:502812-537892 - | 35,081 | 7 | Yes |
| Odorant-binding protein 2 | HhalOBP2, 528 bp, 175 aa, GenBank KT875738 | NW_014467463.1:274127-276921 - | 2,795 | 7 | Yes |
| Odorant-binding protein 3 | HhalOBP3, 594 bp, 197 aa, GenBank KT875739 | NW_014466590.1:527289-547757 - | 20,469 | 9 | Yes |
| Odorant-binding protein 4 | HhalOBP4, 639 bp, 212 aa, GenBank KT875740 | NW_014466586.1:1744142-1762242 + | 18,101 | 9 | Yes |
| Odorant-binding protein 5 | HhalOBP5, 1002 bp, 333 aa, GenBank KT875741 | NW_014466467.1:2246638-2268553 - | 21,916 | 8 | Yes |
| Odorant-binding protein 6 | HhalOBP6, 423 bp, 140 aa, GenBank KT875742 | NW_014466445.1:1458342-1463404 - | 5,063 | 7 | Yes |
| Odorant-binding protein 7 | HhalOBP7, 426 bp, 141 aa, GenBank KT875743 | NW_014467659.1:67017-70346 + | 3,330 | 7 | Yes |
| Odorant-binding protein 8 | HhalOBP8, 432 bp, 143 aa, GenBank KT875744 | NW_014466445.1:1923150-1943338 - | 20,189 | 7 | Yes |
| Odorant-binding protein 9 | HhalOBP9, 615 bp, 204 aa, GenBank KT875745 | NW_014466634.1:320624-328544 - | 7,921 | 9 | Yes |
| Odorant-binding protein 10 | HhalOBP10, 597 bp, 198 aa, GenBank KT875746 | NW_014466922.1:323177-358530 - | 35,354 | 8 | Yes |
| Odorant-binding protein 11 | HhalOBP11, 420 bp, 139 aa, GenBank KT875747 | NW_014466445.1:1836424-1852919 - | 16,496 | 7 | Yes |
| Odorant-binding protein 12 | HhalOBP12, 450 bp, 149 aa, GenBank KT875748 | NW_014466445.1:1773859-1787043 - | 13,185 | 7 | Yes |
| Odorant-binding protein 13 | HhalOBP13, 573 bp, 190 aa, GenBank KT875749 | NW_014467401.1:54709-58653 - | 3,945 | 9 | Yes |
| Odorant-binding protein 14 | HhalOBP14, 558 bp, 148 aa, GenBank KT875750 | NW_014467090.1:511996-521873 - | 9,878 | 7 | Yes |
| Odorant-binding protein 15 isoform X1 | Predicted isoform X1, 495 bp, 164 aa,  GenBank XP_014283307.1 | NW_014467090.1:594310-599865 - | 5,556 | 7 | Yes |
| Odorant-binding protein 15 isoform X2 | Predicted isoform X2, 543 bp, 180 aa, GenBank XP_014283307.1 | NW_014467090.1:594310-599913 - | 5,604 | 7 | Yes |
| Odorant-binding protein 15 isoform X3 | HhalOBP15, 450 bp, 149 aa, GenBank KT875751 | NW_014467090.1:594310-599820 - | 5,511 | 7 | Yes |
| Odorant-binding protein 16 isoform X1 | Predicted isoform X1, 411 bp, 136 aa, GenBank XP_014283314.1 | NW_014467090.1:564255-573228 - | 8,974 | 6 | Yes |
| Odorant-binding protein 16 isoform X2 | HhalOBP16, 534 bp, 177 aa, GenBank KT875752 | NW_014467090.1:564255-573228 - | 8,974 | 7 | Yes |
| Odorant-binding protein 17 | HhalOBP17, 483 bp, 150 aa, GenBank KT875753 | NW_014466445.1:1724141-1732250 - | 8,110 | 7 | Yes |
| Odorant-binding protein 18 | HhalOBP18, 777 bp, 241 aa, GenBank KT875754 | NW_014466850.1:589741-596494 + | 6,754 | 9 | Yes |
| Odorant-binding protein 19 | HhalOBP19, 441 bp, 140 aa, GenBank KT875755 | NW_014467659.1:45328-48919 + | 3,592 | 7 | Yes |
| Odorant-binding protein 20 | HhalOBP20, 444 bp, 138 aa, GenBank KT875756 | NW_014467090.1:581537-584971 - | 3,435 | 7 | Yes |
| Odorant-binding protein 21 | HhalOBP21, 474 bp, 150 aa, GenBank KT875757 | NW_014466445.1:1474481-1511565 - | 37,085 | 5 | Yes |
| Odorant-binding protein 22 isoform X1 | Predicted isoform X1, 504 bp, 167 aa, GenBank XP_014288406.1 | NW_014467545.1:182901-185210 + | 2,310 | 6 | Yes |
| Odorant-binding protein 22 isoform X2 | Predicted isoform X2, 498 bp, 165 aa, GenBank XP_014288407.1 | NW_014467545.1:182901-185210 + | 2,310 | 6 | Yes |
| Odorant-binding protein 22 isoform X3 | Predicted isoform X3, 489 bp, 162 aa, GenBank XP_014288408.1 | NW_014467545.1:182916-185210 + | 2,295 | 6 | Yes |
| Odorant-binding protein 22 isoform X4 | HhalOBP22, 441 bp, 146 aa, GenBank KT875758 | NW_014467545.1:182958-185210 + | 2,253 | 6 | Yes |
| Odorant-binding protein 23 isoform X1 | Predicted isoform X1, 432 bp, 143 aa, GenBank XP_014281335.1 | NW_014466445.1:1444668-1449235 - | 4,568 | 7 | Yes |
| Odorant-binding protein 23 isoform X2 | HhalOBP23, 435 bp, 144 aa, GenBank KT875759 | NW_014466445.1:1444668-1449235 - | 4,568 | 7 | Yes |
| Odorant-binding protein 24 | HhalOBP24, 438 bp, 145 aa, GenBank KT875760 | NW_014466538.1:256562-261770 + | 5,209 | 7 | Yes |
| Odorant-binding protein 25 | HhalOBP25, 447 bp, 148 aa, GenBank KT875761 | NW_014467090.1:545641-553416 - | 7,776 | 7 | Yes |
| Odorant-binding protein 26 | HhalOBP26, 450 bp, 149 aa, GenBank KT875762 | NW_014467326.1:658301-665986 - | 7,686 | 7 | Yes |
| Odorant-binding protein 27 | HhalOBP27, 447 bp, 148 aa, GenBank KT875763 | NW_014466445.1:1643916-1654936 - | 11,021 | 8 | Yes |
| Odorant-binding protein 28 | HhalOBP28, 591 bp, 196 aa, GenBank KT875764 | NW_014466590.1:552444-574681 - | 22,238 | 9 | Yes |
| Odorant-binding protein 29 | HhalOBP29, 714 bp, 237 aa, GenBank KT875765 | NW_014466942.1:820823-842807 - | 21,985 | 8 | Yes |
| Odorant-binding protein 30 | HhalOBP30, 411 bp, 148 aa, GenBank KU315186 and XP_014282895.1 | NW_014466446.1:1911846-1924985 + | 13,140 | 7 | Yes |
| Odorant-binding protein 31 isoform X1 | Predicted isoform X1, 387 bp, 128 aa, GenBank XP_014271863.1 | NW_014466538.1:244127-251266 - | 7,140 | 7 | Yes |
| Odorant-binding protein 31 isoform X2 | Predicted isoform X2, 444 bp, 147 aa, GenBank XP_014271861.1 | NW_014466538.1:244127-251266 - | 7,140 | 7 | Yes |
| Odorant-binding protein 32 | Predicted, 408 bp, 135 aa, GenBank XP_014281385.1 | NW_014466445.1:1897217-1912851 - | 15,635 | 7 | Yes |
| Odorant-binding protein 33 | Predicted, 447 bp, 148 aa, GenBank XP_014282242.1 | NW_014466445.1:1604243-1617918 - | 13,676 | 7 | Yes |
| Odorant-binding protein 34 | Predicted, 417 bp, 138 aa, GenBank XP_014281486.1 | NW_014466445.1:1887706-1891259 - | 3,554 | 7 | Yes |
| Odorant-binding protein 35 | Predicted, 450 bp, 149 aa, GenBank XP_014281517.1 | NW_014466445.1:1576992-1594200 - | 17,209 | 7 | Yes |
| Odorant-binding protein 36 | Predicted, 441 bp, 146 aa, GenBank XP_014281372.1 | NW_014466445.1:552785-574353 + | 21,569 | 7 | Yes |
| Odorant-binding protein 37 | Predicted, 471 bp, 156 aa, GenBank XP_014285255.1 | NW_014466452.1:2251529-2255072 + | 3,544 | 7 | Yes |
| Odorant-binding protein 38 | Predicted, 447 bp, 148 aa, GenBank XP_014285315.1 | NW_014466452.1:2262566-2265008 + | 2,443 | 7 | Yes |
| Odorant-binding protein 39 | Predicted, 399 bp, 132 aa, GenBank XP_014273335.1 | NW_014466586.1:1818210-1826101 - | 7,892 | 7 | Yes |
| Odorant-binding protein 40 | Predicted, 411 bp, 136 aa, GenBank XP_014293023.1 | NW_014469125.1:1629-10567 + | 8,939 | 7 | Yes |
| Odorant-binding protein 41 | Predicted, 447 bp, 148 aa, GenBank XP_014277885.1 | NW_014466787.1:413678-431828 + | 18,151 | 7 | Yes |
| Odorant-binding protein 42 isoform X1 | Predicted isoform X1, 393 bp, 130 aa, GenBank XP_014288227.1 | NW_014467521.1:299565-303654 + | 4,090 | 7 | Yes |
| Odorant-binding protein 42 isoform X2 | Predicted isoform X2, 390 bp, 129 aa, GenBank XP_014288228.1 | NW_014467521.1:301087-303654 + | 2,568 | 7 | Yes |
| Odorant-binding protein 43 | Predicted, 387 bp, 128 aa, GenBank XP_014288229.1 | NW_014467521.1:417765-422736 - | 4,972 | 7 | Yes |
| Odorant-binding protein 44 | Predicted, 387 bp, 128 aa, GenBank XP_014288230.1 | NW_014467521.1:389308-391437 - | 2,130 | 7 | Yes |
| Odorant-binding protein 45 | Predicted, 384 bp, 127 aa, GenBank XP_014288234.1 | NW_014467521.1:327737-333630 + | 5,894 | 7 | Yes |
| Odorant-binding protein 46 | Predicted, 387 bp, 128 aa, GenBank XP_014288235.1 | NW_014467521.1:366542-371240 - | 4,699 | 7 | Yes |
| Odorant-binding protein 47 | Predicted, 396 bp, 131 aa, GenBank XP_014291808.1 | NW_014468285.1:5030-8275 + | 3,246 | 7 | Yes |
| Odorant-binding protein 48 isoform X1 | Predicted isoform X1, 387 bp, 128 aa, GenBank XP_014291806.1 | NW_014468285.1:11235-15130 - | 3,896 | 7 | Yes |
| Odorant-binding protein 48 isoform X2 | Predicted isoform X2, 387 bp, 128 aa, GenBank XP_014291807.1 | NW_014468285.1:11235-27084 - | 15,850 | 7 | Yes |
| Pseudo odorant-binding protein 1 | GenBank XP_014281397.1 | NW_014466445.1:1525165-1540347 - | 15,183 | 7 | No |
| Pseudo odorant-binding protein 2 | GenBank XP_014276989.1 | NW_014466739.1:404385-412901 - | 8,517 | 7 | No |
| Pseudo odorant-binding protein 3 | GenBank XP_014288225.1 | NW_014467521.1:345525-348448 + | 2,924 | 7 | No |
| Pseudo odorant-binding protein 4 | GenBank XP_014288233.1 | NW_014467521.1:356044-360212 + | 4,169 | 7 | No |
| Pseudo odorant-binding protein 5 | GenBank XP_014288237.1 | NW_014467521.1:311396-314697 + | 3,302 | 7 | No |
| Pseudo odorant-binding protein 6 | GenBank XP_014280145.1 | NW_014466892.1:160901-179231 - | 18,331 | 6 | No |
| Pseudo odorant-binding protein 7 | GenBank XP_014292092.1 | NW_014468422.1:24948-31613 + | 6,666 | 7 | No |

**Table S13.** Primer sequences used to validate the HhalOBP gene annotations.

| **GenBank accession** | **Sequence (5'>3')** |
| --- | --- |
| XP_014271861.1_F | AGTGTTGGCTCAGTTGTG |
| XP_014271861.1_R | GCTAATTCGCAGGCATCT |
| XP_014271863.1_F | AGTGTTGGCTCAGTTGTG |
| XP_014271863.1_R | GCTAATTCGCAGGCATCT |
| XP_014273335.1_F | GAGACAGAGGTTCATCACTA |
| XP_014273335.1_R | GCTCATTAGGCATTCTTCAT |
| XP_014276989.1_F | TGAGAAATGTGGGAGAATGT |
| XP_014276989.1_R | GCTTGGCATCTGCTTCAA |
| XP_014276990.1_F | TTTACCTTTCGTTCCACACT |
| XP_014276990.1_R | GCTTGGCATCTGCTTCAA |
| XP_014277885.1_F | GGCTATGGTATTGGCAGTT |
| XP_014277885.1_R | GACTTCTTCATCCTGATTCAC |
| XP_014280145.1_F | TGAGTCAATTCGATGTTCCT |
| XP_014280145.1_R | AGACCAGTTCCTAACTTCAC |
| XP_014281335.1_F | AAATGGAAGTGCCGAAGG |
| XP_014281335.1_R | AAGGAGATCAGACTGTGTTG |
| XP_014281372.1_F | GGATGATATTATGGACGACTTG |
| XP_014281372.1_R | TAAGAGCCGACGTAAGGT |
| XP_014281385.1_F | CCTATCGTTGACGCCTTC |
| XP_014281385.1_R | GCTCTTCCTGTAACCATCC |
| XP_014281397.1_F | ATGACCCTGCTGAGTATAAC |
| XP_014281397.1_R | TCTCGCAGACTCTAATGAC |
| XP_014281486.1_F | CAGCGGAATTACCAATGTTA |
| XP_014281486.1_R | TCCATCCTACTGCTTTGTAT |
| XP_014281517.1_F | AAGAATGCCAAGCTGAGG |
| XP_014281517.1_R | CGACCAAATCATAAGCGTAA |
| XP_014281568.1_F | AAGTGAAGGCTCTCAATCC |
| XP_014281568.1_R | AGTTCGCAGACATCAGTATT |
| XP_014282204.1_F | TCTGTATCACCACTCTTCTG |
| XP_014282204.1_R | TGCCTTCTCAACTTCTTCA |
| XP_014282242.1_F | TAGTGTTGGCGTCATGTG |
| XP_014282242.1_R | CCCTTAATGGCTTGCTCTA |
| XP_014282252.1_F | ACATCCTTGTCCGATTGC |
| XP_014282252.1_R | GTTCACAGAGGCTTGGTT |
| XP_014283301.1_F | CCAGCAGGTCTCAGTCTA |
| XP_014283301.1_R | GCCATGTAGCACTTAGCA |
| XP_014283307.1_F | GTCAAGCCAGGAATAATAAGTC |
| XP_014283307.1_R | GTCCATCTGTAACCAAACCA |
| XP_014283314.1_F | CCAGAACAGCCATAGGTAT |
| XP_014283314.1_R | AATTCATCAGCACTAAGAGG |
| XP_014285255.1_F | GAGCCTCTCCTTCTTGTG |
| XP_014285255.1_R | GTGATAATGCCAATGACAGT |
| XP_014285315.1_F | GCTTATATTGTTGCGTTGAC |
| XP_014285315.1_R | TGGCTGATCTTGTATTGTTC |
| XP_014287840.1_F | AGCACTAGCAAACAGACA |
| XP_014287840.1_R | TCGGAATGGCTTGATTAGA |
| XP_014288225.1_F | GACGAAGAGTTGAAGAAGAAG |
| XP_014288225.1_R | GCAGAGATAAGAGAAGAGTGT |
| XP_014288227.1_F | CGGATGGCTCTATTGACAA |
| XP_014288227.1_R | GCCTTAGTATCCACGCATT |
| XP_014288228.1_F | GTGAAGAGAACGCAATTAGA |
| XP_014288228.1_R | ATAGAGCCATCCGCCTTA |
| XP_014288229.1_F | CTGTCCTGTCAGATGATTATG |
| XP_014288229.1_R | AAGCACACTTAGCCTCAT |
| XP_014288230.1_F | GCTTTGGCTTGTTTATTCG |
| XP_014288230.1_R | TTCACTTCCGCATCCTTAT |
| XP_014288233.1_F | TACTCCTAACAGCACATTCT |
| XP_014288233.1_R | CTTCTTCTTCAACTCCTCATC |
| XP_014288234.1_F | CAACGCATTCTGTCTTATCA |
| XP_014288234.1_R | GCATCGCATTCGTCAATT |
| XP_014288235.1_F | TGACGGAACCATTGACAG |
| XP_014288235.1_R | CGATTGCCTTCAGATACTTC |
| XP_014288236.1_F | CAGTGCAATCTGTTCAATCA |
| XP_014288236.1_R | ACATCGCATTCATCAATAGC |
| XP_014288237.1_F | GGAATATTAGTTGTGCTCGC |
| XP_014288237.1_R | TCCTTGTTACACACTGCTTT |
| XP_014288406.1_F | TTCTGCTTACCATTCCATCA |
| XP_014288406.1_R | GTTGCCATTATCACTCTGTC |
| XP_014288407.1_F | TTCTGCTTACCATTCCATCA |
| XP_014288407.1_R | GTTGCCATTATCACTCTGTC |
| XP_014288408.1_F | TTCTGCTTACCATTCCATCA |
| XP_014288408.1_R | GTTGCCATTATCACTCTGTC |
| XP_014289168.1_F | ATGGCTGAGGAGGAGAAG |
| XP_014289168.1_R | GACGATGGTGGAGGAGAT |
| XP_014291806.1_F | ACATTCTGTTCTGTCACTGA |
| XP_014291806.1_R | CACACTTAGCTGCCTTGG |
| XP_014291807.1_F | GACAACAACAGCAGTTATAGG |
| XP_014291807.1_R | CAGTGACAGAACAGAATGTG |
| XP_014291808.1_F | CAAGGCATACCAACAACTG |
| XP_014291808.1_R | CGTGAGTGACAGAACAGAA |
| XP_014292092.1_F | AGCAAGAACATCACGAAGT |
| XP_014292092.1_R | AGAACATCCTCAGCAGTAAG |
| XP_014293023.1_F | TAGTAGTCTTCTGGACGAGA |
| XP_014293023.1_R | TGAAGCAAGCCATATAACAC |

**Table S14.** Correspondences between *H. halys* predicted protein identifiers and cathepsin labels.

| Cathepsin nomenclature | NCBI identifier | Description |
| --- | --- | --- |
| Hh_CatI | XP_014271834.1 | cathepsin L1-like |
| Hh_Cat.ss.uL2.14 | XP_014272392.1 | cathepsin L1 |
| Hh_Cat.ss.uL2.15 | XP_014290635.1 | cathepsin L1 |
| Hh_Cat.ss.uL1.8 | XP_014290884.1 | cathepsin L1-like isoform X1 |
| Hh_Cat.ss.uL1.11 | XP_024216457.1 | cathepsin L1-like |
| Hh_Cat.ss.uL2.12 | XP_014291518.1 | cathepsin L1-like |
| Hh_Cat.ss.uL1.1 | XP_014273553.1 | cathepsin L1-like |
| Hh_Cat.ss.uL1.2 | XP_014273554.1 | cathepsin L1-like |
| Hh_Cat.ss.uL1.3 | XP_014273554.1 | cathepsin L1-like |
| Hh_Cat.ss.uL1.4 | XP_014273554.1 | cathepsin L1-like |
| Hh_Cat.ss.uL1.5 | XP_014273555.1 | cathepsin L1-like |
| Hh_Cat.ss.uL2.8 | XP_014292125.1 | cathepsin L1-like |
| Hh_Cat.ss.uL2.9 | XP_014292126.1 | cathepsin L1-like |
| Hh_Cat.ss.uL2.10 | XP_014292129.1 | cathepsin L1-like |
| Hh_Cat.ss.uL1.6 | XP_024219582.1 | cathepsin L1-like |
| Hh_Cat.ss.uL2.16 | XP_024219377.1 | cathepsin L1 isoform X1 |
| Hh_Cat.ss.uL3.1 | XP_014279027.1 | cathepsin L1 |
| Hh_Cat.ss.uL1.7 | XP_014292799.1 | cathepsin L1 |
| Hh_Cat.ss.uL3.2 | XP_014282138.1 | cathepsin L1-like |
| Hh_CatO | XP_024215418.1 | cathepsin L1 |
| Hh_CatB | XP_014290817.1 | cathepsin B |
| Hh_CatF | XP_014278765.1 | cathepsin L1 |
| Hh_Cat.ss.uB1.1 | XP_014289457.1 | cathepsin B-like |
| Hh_Cat.ss.uB1.2 | XP_014289471.1 | cathepsin B |
| Hh_Cat.ss.uB1.3 | XP_014289487.1 | cathepsin B-like cysteine proteinase 4 |
| Hh_Cat.ss.uB1.4 | XP_024215294.1 | cathepsin B-like |
| Hh_Cat.ss.uB1.5 | XP_024215294.1 | cathepsin B-like |
| Hh_Cat.ss.uB1.6 | XP_014289247.1 | cathepsin B-like isoform X3 |
| Hh_Cat.ss.uL1.9 | XP_014281817.1 | cathepsin L1 |
| Hh_Cat.ss.uL2.1 | XP_014282107.1 | cathepsin L1-like isoform X1 |
| Hh_Cat.ss.uL2.2 | XP_014282107.1 | cathepsin L1-like isoform X1 |
| Hh_Cat.ss.uL2.3 | XP_014282108.1 | cathepsin L1-like isoform X2 |
| Hh_Cat.ss.uL2.4 | XP_014282110.2 | cathepsin L1-like |
| Hh_Cat.ss.uL2.5 | XP_014282105.1 | cathepsin L1-like |
| Hh_Cat.ss.uL2.6 | XP_014282106.1 | cathepsin L1-like |
| Hh_Cat.ss.uL2.7 | XP_014282112.1 | cathepsin L1-like |
| Hh_CatLl | XP_014283673.1 | cathepsin L1-like |
| Hh_Cat.ss.uL1.10 | XP_024217634.1 | cathepsin L1-like |
| Hh_Cat.ss.uL2.11 | XP_024216457.1 | cathepsin L1-like |
| Hh_Cat.ss.uL2.17 | XP_014286294.1 | cathepsin L1-like |
| Hh_Cat.ss.uL2.13 | XP_014286295.1 | cathepsin L1 |

**Table S15.** A total of 64 salivary effector proteins were identified in the *H. halys* genome.

| **Gene symbol** | **Protein Reference** | **Pea aphid homolog** | **Blast E-value** | **Sequence description** | **Greatest identity %** | **Greatest positive %** | **Greatest HSP length** | **Blast bit score** |
| --- | --- | --- | --- | --- | --- | --- | --- | --- |
| LOC106691878 | XP_014293266.1 | ACYPI003278 | 3.7ee-69 | 15 kDa selenoprotein-like | 66.9 | 79.9 | 154 | 210.3 |
| LOC106682875 | XP_014279464.1 | ACYPI009886 | 0 | acetylcholinesterase-like | 67.5 | 82.8 | 571 | 832.0 |
| LOC106686134 | XP_014284771.1 | ACYPI002258 | 0 | aminopeptidase N-like | 38.1 | 60.7 | 885 | 664.5 |
| LOC106681465 | XP_014277274.1 | ACYPI007204 | 0 | angiotensin-converting enzyme-like | 62.0 | 79.3 | 485 | 670.6 |
| LOC106679717 | XP_014274514.1 | ACYPI004198 | 1.1e-76 | apolipophorins | 27.3 | 49.5 | 744 | 275.0 |
| LOC106681713 | XP_014277663.1 | ACYPI008001 | 1.6e-45 | armet/mesencephalic astrocyte-derived neurotrophic factor homolog | 51.7 | 70.1 | 174 | 151.0 |
| LOC106681650 | XP_014277576.1 | ACYPI002622 | 0 | calreticulin | 75.9 | 86.2 | 340 | 520.4 |
| LOC106686022 | XP_014284587.1 | ACYPI001238 | 0 | carboxypeptidase E-like | 59.5 | 74.1 | 414 | 520.4 |
| LOC106690036 | XP_014290885.1 | ACYPI000003 | 3.2e-139 | cathepsin B | 60.6 | 73.8 | 316 | 402.9 |
| LOC106682597 | XP_014279027.1 | ACYPI006974 | 3.0e-114 | cathepsin L1 | 66.5 | 80.0 | 229 | 337.0 |
| LOC106689042 | XP_014289277.1 | ACYPI005292 | 0 | CD109 antigen-like isoform X4 | 60.4 | 77.7 | 1326 | 1640.9 |
| LOC106681659 | XP_014277588.1 | ACYPI001365 | 2.1e-113 | chitinase-like protein EN03 isoform X3 | 44.3 | 59.0 | 406 | 342.8 |
| LOC106691864 | XP_014293249.1 | ACYPI001579 | 4.7e-128 | chondroitin proteoglycan-2-like | 91.5 | 96.3 | 189 | 375.9 |
| LOC106688918 | XP_014289105.1 | ACYPI000473 | 2.3e-77 | circadian clock-controlled protein-like | 48.5 | 69.5 | 233 | 238.4 |
| LOC106685386 | XP_014283503.1 | ACYPI005526 | 0 | contactin | 61.2 | 77.7 | 1183 | 1478.0 |
| LOC106685481 | XP_014283673.1 | ACYPI003954 | 5.4e-170 | digestive cysteine proteinase 1 | 53.2 | 69.8 | 452 | 496.1 |
| LOC106677514 | XP_014270980.1 | ACYPI006124 | 3.2e-179 | dnaJ homolog subfamily C member 3 | 53.5 | 71.0 | 492 | 516.5 |
| LOC106682353 | XP_014278639.1 | ACYPI001775 | 1.6e-23 | endocuticle structural glycoprotein SgAbd-2-like | 48.0 | 66.0 | 100 | 95.1 |
| LOC106682465 | XP_014278818.1 | ACYPI007954 | 3.8e-120 | endoplasmic reticulum lectin 1 | 42.1 | 62.9 | 455 | 366.3 |
| LOC106678053 | XP_014271832.1 | ACYPI000995 | 9.7e-76 | endoplasmic reticulum resident protein 29 | 48.2 | 67.3 | 243 | 233.4 |
| LOC106683332 | XP_014280207.1 | ACYPI000119 | 1.3e-165 | endoplasmic reticulum resident protein 44 | 59.6 | 71.7 | 381 | 475.3 |
| LOC106681661 | XP_014277591.1 | ACYPI009915 | 0 | endoplasmin | 72.2 | 84.2 | 709 | 1004.2 |
| LOC106680807 | XP_014276245.1 | ACYPI002576 | 8.1e-24 | ER membrane protein complex subunit 10 | 31.9 | 48.7 | 218 | 96.3 |
| LOC106689333 | XP_014289747.1 | ACYPI007083 | 0 | fasciclin-1 isoform X9 | 47.1 | 65.6 | 650 | 580.5 |
| LOC106692988 | XP_014294773.1 | ACYPI009403 | 1.0e-62 | ferritin subunit | 51.0 | 69.1 | 186 | 198.0 |
| LOC106679424 | XP_014274065.1 | ACYPI003483 | 2.1e-104 | FK506-binding protein 2 | 74.9 | 84.9 | 199 | 305.1 |
| LOC106691013 | XP_014292137.1 | ACYPI008158 | 1.8e-159 | gelsolin, cytoplasmic | 62.5 | 80.3 | 354 | 471.9 |
| LOC106688290 | XP_014288163.1 | ACYPI002729 | 0 | glycosyltransferase-like protein LARGE1 | 67.5 | 81.3 | 709 | 1010.8 |
| LOC106687064 | XP_014286232.1 | ACYPI008996 | 0 | hypoxia up-regulated protein 1 | 40.8 | 55.6 | 952 | 558.9 |
| LOC106690826 | XP_014291894.1 | ACYPI008756 | 2.4e-178 | lachesin | 71.9 | 85.6 | 325 | 503.8 |
| LOC106684151 | XP_014281554.1 | ACYPI001019 | 1.6e-41 | mucin-2-like | 74.1 | 83.1 | 152 | 167.9 |
| LOC106680197 | XP_014275215.1 | ACYPI008792 | 5.4e-45 | microsomal triglyceride transfer protein large subunit | 28.6 | 47.2 | 544 | 171.8 |
| LOC106681352 | XP_014277113.1 | ACYPI000097 | 1.6e-26 | mp10/ejaculatory bulb-specific protein 3-like | 45.9 | 68.4 | 98 | 99.0 |
| LOC106688181 | XP_014288014.1 | ACYPI005411 | 1.6e-165 | neurogenic locus notch homolog protein 2 | 57.2 | 69.7 | 406 | 475.7 |
| LOC106679031 | XP_014273428.1 | ACYPI009457 | 0 | neutral alpha-glucosidase AB | 52.9 | 69.5 | 900 | 990.7 |
| LOC106679755 | XP_014274569.1 | ACYPI003852 | 0 | pancreatic lipase-related protein 2-like | 48.0 | 68.5 | 528 | 547.0 |
| LOC106692440 | XP_014293875.1 | ACYPI009369 | 3.0e-60 | pancreatic triacylglycerol lipase-like | 37.2 | 55.8 | 284 | 198.7 |
| LOC106692485 | XP_014293941.1 | ACYPI000817 | 1.2e-108 | peroxidase-like isoform X1 | 33.4 | 52.7 | 585 | 345.5 |
| LOC106681766 | XP_014277748.1 | ACYPI003960 | 9.2e-138 | peroxiredoxin-2 | 77.0 | 86.6 | 239 | 391.7 |
| LOC106678736 | XP_014272949.1 | ACYPI009721 | 2.9e-99 | prosaposin | 40.5 | 52.4 | 640 | 331.6 |
| LOC106689346 | XP_014289762.1 | ACYPI000760 | 2.6e-84 | protein canopy 4 | 66.0 | 77.5 | 207 | 252.7 |
| LOC106684721 | XP_014282450.1 | ACYPI005702 | 1.0e-32 | protein cereblon homolog | 44.2 | 68.1 | 138 | 117.5 |
| LOC106678635 | XP_014272751.1 | ACYPI009755 | 0 | protein disulfide-isomerase | 53.8 | 72.7 | 472 | 534.6 |
| LOC106677432 | XP_014270846.1 | ACYPI005594 | 0 | protein disulfide-isomerase A3 | 62.4 | 80.2 | 446 | 605.9 |
| LOC106686982 | XP_014286089.1 | ACYPI008926 | 1.1e-172 | protein disulfide-isomerase A6 | 60.8 | 73.4 | 395 | 493.4 |
| LOC106690160 | XP_014290979.1 | ACYPI008182 | 1.6e-98 | protein takeout-like | 68.9 | 86.5 | 222 | 292.0 |
| LOC106690949 | XP_014292045.1 | ACYPI000479 | 1.0e-168 | protein yellow isoform X2 | 56.5 | 76.0 | 390 | 484.2 |
| LOC106680598 | XP_014275905.1 | ACYPI001857 | 8.6e-112 | protein yellow-like isoform X1 | 42.9 | 60.0 | 392 | 339.3 |
| LOC106682432 | XP_014278766.1 | ACYPI000376 | 1.7e-123 | putative cysteine proteinase CG12163 isoform X2 | 48.9 | 65.1 | 436 | 386.7 |
| LOC106677191 | XP_014270440.1 | ACYPI002876 | 4.2e-28 | putative defense protein 3 | 38.8 | 55.3 | 140 | 105.5 |
| LOC106688517 | XP_014288497.1 | ACYPI007347 | 1.0e-39 | repetitive proline-rich cell wall protein 2-like | 55.7 | 65.6 | 211 | 145.6 |
| LOC106681164 | XP_014276825.1 | ACYPI001446 | 0 | sarcalumenin | 77.2 | 90.2 | 439 | 766.9 |
| LOC106692986 | XP_014294772.1 | ACYPI007511 | 7.1e-50 | soma ferritin | 39.7 | 58.1 | 232 | 166.0 |
| LOC106682098 | XP_014278259.1 | ACYPI007065 | 4.4e-76 | stromal cell-derived factor 2 | 53.3 | 67.8 | 211 | 232.6 |
| LOC106681155 | XP_014276814.1 | ACYPI003921 | 2.6e-58 | superoxide dismutase [Cu-Zn]-like isoform X2 | 58.9 | 71.2 | 159 | 186.0 |
| LOC106678839 | XP_014273103.1 | ACYPI001938 | 5.1e-34 | transport and Golgi organization protein 1 | 29.2 | 48.4 | 505 | 142.9 |
| LOC106681721 | XP_014277675.1 | ACYPI002298 | 0 | trehalase-like | 51.9 | 70.6 | 533 | 597.8 |
| LOC106679932 | XP_014274819.1 | ACYPI000217 | 0 | uncharacterized protein | 83.1 | 90.7 | 522 | 929.9 |
| LOC106689851 | XP_014290523.1 | ACYPI009786 | 6.2e-92 | uncharacterized protein | 66.5 | 80.1 | 190 | 273.5 |
| LOC106688070 | XP_014287840.1 | ACYPI006147 | 2.4e-31 | uncharacterized protein | 45.1 | 63.9 | 121 | 116.3 |
| LOC106688773 | XP_014288888.1 | ACYPI006373 | 2.4e-27 | uncharacterized protein | 42.7 | 55.9 | 121 | 102.4 |
| LOC106681652 | XP_014277590.1 | ACYPI006963 | 5.5e-36 | uncharacterized protein | 37.3 | 54.4 | 186 | 129.0 |
| LOC106685454 | XP_014283619.1 | ACYPI004272 | 2.1e-49 | uncharacterized protein | 29.8 | 46.3 | 592 | 182.6 |
| LOC106684787 | XP_014282547.1 | ACYPI008471 | 2.8e-24 | uncharacterized protein | 25.4 | 43.1 | 300 | 104.4 |

**Table S16.** A select subset of 15 *H. halys* salivary effector proteins having variable expression levels between nymphal and adult stages (up- or down-regulation).

| **Protein** | **Transcript** | **2nd_Instar**  **USDA^*^** | **4th_Instar**  **USDA^*^** | **Adult_Male**  **USDA^*^** | **Adult_Female**  **USDA^*^** | **Juveniles UMD^*^** | **Adults UMD^*^** | **Antennae EMBRAPA^*^** | **Description** | **Test Statistic (dN-dS)^#^** |
| --- | --- | --- | --- | --- | --- | --- | --- | --- | --- | --- |
| XP_014274514.1 | XM_014419028.1 | 96.62 | 80.80 | 685.61 | 318.87 | 913.58 | 1,011.58 | 12.11 | apolipophorins (LOC106679717) | -26.48 |
| XP_014292137.1 | XM_014436651.1 | 103.30 | 133.28 | 516.41 | 227.37 | 542.25 | 797.40 | 252.93 | gelsolin, cytoplasmic (LOC106691013) | -22.11 |
| XP_014293875.1 | XM_014438389.1 | 21.27 | 25.75 | 112.64 | 28.04 | 35.43 | 89.91 | 0.56 | pancreatic triacylglycerol lipase-like (LOC106692440) | -11.61 |
| XP_014292044.1 | XM_014436558.1 | 15.63 | 33.25 | 61.48 | 88.83 | 171.94 | 80.03 | 46.86 | protein yellow (LOC106690949), transcript variant X1 | 19.17 |
| XP_014272949.1 | XM_014417463.1 | 166.17 | 242.25 | 457.36 | 300.35 | 480.21 | 724.83 | 132.97 | prosaposin (LOC106678736) | -19.35 |
| XP_014290817.1 | XM_014435399.1 | 38.02 | 126.19 | 181.86 | 134.82 | 13.86 | 21.85 | 5.30 | cathepsin B (LOC106690036),transcript variant X2 | -15.16 |
| XP_014293249.1 | XM_014437763.1 | 47.81 | 85.70 | 94.97 | 151.90 | 254.73 | 42.78 | 22.60 | chondroitin proteoglycan-2-like (LOC106691864) | -27.31 |
| XP_014271832.1 | XM_014416346.1 | 46.35 | 38.52 | 108.97 | 39.34 | 43.14 | 79.03 | 19.70 | endoplasmic reticulum resident protein 29 (LOC106678053) | -12.11 |
| XP_014279027.1 | XM_014423541.1 | 455.81 | 921.59 | 1,059.94 | 1,026.02 | 762.56 | 799.13 | 375.82 | cathepsin L1 (LOC106682597) | -14.93 |
| XP_014270440.1 | XM_014414954.1 | 282.38 | 508.64 | 91.80 | 275.55 | 455.94 | 87.36 | 1,728.97 | putative defense protein 3 (LOC106677191) | -8.62 |
| XP_014290523.1 | XM_014435037.1 | 259.30 | 432.33 | 102.57 | 190.48 | 944.95 | 10.33 | 59.70 | uncharacterized (LOC106689851) | -16.63 |
| XP_014288014.1 | XM_014432528.1 | 162.62 | 120.27 | 38.14 | 60.89 | 35.51 | 20.40 | 45.15 | neurogenic locus notch homolog protein 2 (LOC106688181) | -23.04 |
| XP_014287840.1 | XM_014432354.1 | 1,344.38 | 355.27 | 285.16 | 280.78 | 386.69 | 688.66 | 14,128.48 | uncharacterized (LOC106688070) | -5.97 |
| XP_014284771.1 | XM_014429285.1 | 154.38 | 32.51 | 18.99 | 15.64 | 29.31 | 31.61 | 135.41 | aminopeptidase N-like (LOC106686134) | -34.87 |
| XP_014283619.1 | XM_014428133.1 | 89.34 | 20.59 | 9.36 | 8.44 | 10.33 | 14.35 | 38.18 | uncharacterized (LOC106685454) | -24.67 |

* “USDA” refers to samples from [15], “UMD” from [19] and “EMBRAPA” from [18].

# P>0.05 for test of positive selection performed using Nei-Gojobori method [129] through MEGA X [130].

**Table S17.** Gene expression data for *H. halys* glutathione S-transferase genes. “USDA” refers to samples from [15], “UMD” from [19] and “EMBRAPA” from [18]. Yellow highlighting denotes Sigma-class GSTs, red denotes Delta, turquoise denotes Theta, green denotes Microsomal and purple denotes Prostaglandin E synthase.

| Protein | Transcript | Location | 2nd_Instar- USDA | 4th_Instar- USDA | Adult_Male- USDA | Adult_Female- USDA | Juveniles- UMD | Adults- UMD | Antennae- EMBRAPA |
| --- | --- | --- | --- | --- | --- | --- | --- | --- | --- |
| XP_014274229.1 | XM_014418743.1 | LOC106679529 | 0.17 | 0.49 | 0.24 | 0.70 | 0.00 | 0.00 | 0.00 |
| XP_014274230.1 | XM_014418744.1 | LOC106679529 | 9.12 | 21.05 | 7.39 | 25.83 | 3.04 | 1.08 | 0.02 |
| XP_014293557.1 | XM_014438071.1 | LOC106692166 | 58.35 | 19.78 | 40.29 | 34.05 | 101.15 | 108.28 | 274.91 |
| XP_014289737.1 | XM_014434251.1 | LOC106689331 | 11.81 | 13.34 | 15.17 | 20.83 | 10.78 | 10.53 | 5.22 |
| XP_014280850.1 | XM_014425364.1 | LOC106683718 | 33.87 | 30.38 | 30.13 | 36.40 | 34.82 | 33.24 | 22.32 |
| XP_014289953.1 | XM_014434467.1 | LOC106689491 | 0.03 | 0.96 | 105.90 | 0.00 | 5.39 | 85.54 | 0.00 |
| XP_014288573.1 | XM_014433087.1 | LOC106688542 | 755.56 | 663.50 | 810.68 | 765.34 | 852.46 | 919.95 | 710.33 |
| XP_014284599.1 | XM_014429113.1 | LOC106686030 | 0.59 | 0.46 | 0.00 | 0.76 | 1.61 | 0.04 | 150.09 |
| XP_014284598.1 | XM_014429112.1 | LOC106686030 | 1.00 | 0.46 | 0.40 | 0.00 | 0.00 | 0.73 | 4.28 |
| XP_014284597.1 | XM_014429111.1 | LOC106686030 | 0.54 | 0.29 | 0.30 | 0.24 | 0.03 | 0.07 | 13.91 |
| XP_014282487.1 | XM_014427001.1 | LOC106684748 | 81.84 | 73.73 | 79.56 | 69.79 | 96.56 | 93.63 | 76.54 |
| XP_014275015.1 | XM_014419529.1 | LOC106680062 | 8.58 | 2.31 | 1.91 | 2.15 | 1.32 | 2.92 | 1.35 |
| XP_014285058.1 | XM_014429572.1 | LOC106686338 | 165.08 | 47.08 | 26.49 | 24.25 | 20.86 | 58.56 | 54.12 |
| XP_014285065.1 | XM_014429579.1 | LOC106686345 | 17.63 | 49.15 | 47.58 | 58.22 | 68.70 | 32.61 | 0.00 |
| XP_014282246.1 | XM_014426760.1 | LOC106684592 | 78.77 | 60.07 | 72.85 | 81.68 | 63.83 | 74.17 | 125.67 |
| XP_014293590.1 | XM_014438104.1 | LOC106692198 | 35.02 | 19.19 | 21.79 | 30.11 | 36.89 | 13.53 | 54.43 |
| XP_014293434.1 | XM_014437948.1 | LOC106692037 | 87.22 | 26.06 | 17.87 | 15.51 | 10.38 | 22.07 | 90.97 |
| XP_014286985.1 | XM_014431499.1 | LOC106687547 | 44.52 | 14.64 | 9.93 | 14.27 | 64.02 | 15.11 | 13.56 |
| XP_014274135.1 | XM_014418649.1 | LOC106679461 | 80.44 | 27.04 | 36.25 | 27.21 | 104.11 | 124.70 | 279.04 |
| XP_014274136.1 | XM_014418650.1 | LOC106679461 | 16.25 | 5.38 | 3.41 | 5.96 | 29.09 | 8.15 | 4.26 |
| XP_014285059.1 | XM_014429573.1 | LOC106686339 | 41.99 | 24.90 | 20.68 | 26.13 | 17.94 | 17.85 | 26.94 |
| XP_014285062.1 | XM_014429576.1 | LOC106686341 | 532.78 | 159.84 | 76.74 | 101.36 | 61.87 | 96.23 | 32.53 |
| XP_014272706.1 | XM_014417220.1 | LOC106678617 | 120.31 | 56.59 | 55.03 | 76.44 | 58.23 | 43.11 | 21.69 |
| XP_014288095.1 | XM_014432609.1 | LOC106688241 | 895.21 | 429.80 | 144.01 | 334.45 | 451.46 | 418.51 | 0.39 |
| XP_014290097.1 | XM_014434611.1 | LOC106689565 | 15.58 | 47.36 | 82.55 | 40.81 | 28.38 | 47.28 | 408.24 |
| XP_014283897.1 | XM_014428411.1 | LOC106685620 | 4.49 | 2.88 | 2.28 | 1.66 | 6.73 | 4.91 | 0.87 |
| XP_014283896.1 | XM_014428410.1 | LOC106685620 | 24.78 | 8.54 | 8.73 | 8.94 | 18.19 | 34.07 | 8.69 |
| XP_014283893.1 | XM_014428407.1 | LOC106685618 | 1.88 | 5.34 | 1.49 | 2.40 | 0.00 | 1.54 | 0.68 |
| XP_014283892.1 | XM_014428406.1 | LOC106685618 | 21.71 | 21.32 | 27.79 | 21.84 | 16.67 | 80.30 | 43.36 |
| XP_014283894.1 | XM_014428408.1 | LOC106685618 | 2.90 | 3.77 | 1.85 | 2.45 | 0.80 | 2.18 | 4.90 |
| XP_014273946.1 | XM_014418460.1 | LOC106679349 | 5.45 | 2.28 | 22.83 | 2.22 | 6.75 | 27.17 | 2.61 |
| XP_014273949.1 | XM_014418463.1 | LOC106679352 | 10.72 | 4.27 | 23.65 | 1.77 | 3.30 | 2.76 | 0.64 |
| XP_014273000.1 | XM_014417514.1 | LOC106678768 | 0.07 | 0.00 | 0.00 | 0.21 | 0.00 | 0.00 | 0.00 |
| XP_014283899.1 | XM_014428413.1 | LOC106685622 | 1.83 | 0.25 | 0.00 | 0.00 | 5.55 | 0.00 | 19.96 |
| XP_014283900.1 | XM_014428414.1 | LOC106685622 | 1.93 | 1.44 | 0.32 | 33.83 | 5.92 | 70.76 | 0.00 |
| XP_014283902.1 | XM_014428416.1 | LOC106685622 | 255.37 | 66.66 | 11.10 | 10.84 | 185.71 | 28.19 | 0.49 |
| XP_014283901.1 | XM_014428415.1 | LOC106685622 | 318.83 | 184.15 | 142.35 | 111.80 | 210.10 | 122.39 | 371.61 |
| XP_014274300.1 | XM_014418814.1 | LOC106679579 | 2.90 | 2.12 | 2.56 | 1.91 | 3.68 | 3.28 | 1.05 |
| XP_014274299.1 | XM_014418813.1 | LOC106679579 | 14.37 | 13.66 | 11.19 | 15.99 | 21.98 | 24.44 | 26.00 |
| XP_014288362.1 | XM_014432876.1 | LOC106688430 | 123.65 | 68.45 | 56.34 | 68.31 | 66.28 | 65.73 | 11.05 |
| XP_014286727.1 | XM_014431241.1 | LOC106687381 | 0.76 | 0.67 | 0.13 | 0.29 | 0.38 | 0.06 | 49.94 |

**Supplementary Figure Legends**

**Figure S1. Phylogenetic organization of the Hemiptera.** Cladogram of Hemiptera as inferred from Bayesian analyses of mitogenomic sequence data. Reproduced without modification from Li et al. (2017, presented as Figure 1 therein; [131]) under terms of the Creative Commons license (https://creativecommons.org/licenses/by/4.0/).

**Figure S2. Ortholog distributions among hemipterans.** The comparisons were performed on the Hemiptera node orthology dataset from the OrthoDB v10 catalog of orthologs, which comprises a total of 16 species. The number of orthogroups and the average number of genes (in parentheses) are presented for all combinations of the five selected species, and the species-specific counts represent orthology with at least one of the 11 other species. A conserved core of nearly 5,000 orthogroups with on average more than 6,000 genes per species are found in common across all five selected hemipterans.

**Figure S3. Genome assembly quality control.** Scaffold sequence coverage as a function of G+C composition is indicated. The plot exhibits consistent placement of scaffolds within narrow bands of sequencing coverage and [GC], supporting the notion of very few contaminant sequences being present in the assembly. The analysis was conducted using BlobTools [8].

**Figure S4. Hox and Iro-C cluster gene loci.** Clusters are shown to scale and with linkage on scaffolds in the current assembly. Note that the Hox genes, ordered from *lab/Hox1* to *Abd-B/Hox10*, all occur on the reverse strand and are shown in a 3′ to 5′ transcriptional orientation, and with a slightly enlarged *ftz* locus shown for clarity. The Iro-C genes occur on the forward strand with a 5′ to 3′ transcriptional orientation. Where multiple isoforms were annotated (*pb/Hox2, iro*), the larger locus is shown here.

**Figure S5. *Halyomorpha mannosidase* expansion.** Genomic organization, gene phylogeny and proposed duplication mechanisms for a mannosidase enzyme acquired by the host insect via a lateral gene transfer event. Nine copies in total were observed. The maximum likelihood phylogeny clearly resolves three subclades, which largely correspond to gene cluster organization in the genome (note green/ blue/ yellow color-coding). In the nucleotide sequence alignment, the pink ‘X’ denotes the position of conserved splice sites within the 5’-UTR (upper alignment) and between coding exons (lower alignment). Gene structure is also a subclade-specific feature (exon counts per gene are indicated within the genome scaffold view).

**Figure S6. Maximum likelihood phylogenetic tree of selected mannosidase proteins from three bacterial outgroups and three hemipteran species.** Included proteins are: both paralogs from *Oncopeltus fasciatus*, one representative paralog from each subclade in *Halyomorpha halys* (as depicted in main text Figure 2), and the four best, tandem tBLASTn hits in the *Euschistus heros* assembly (accession GCA_003667255.1), using OFAS017153 and the three *H. halys* subclade representatives as BLAST queries. Note that this is only comprehensive for the *Oncopeltus* mannosidase proteins. The hemipteran proteins form a well-supported clade, consistent with a single LGT event at the base of the Pentatomomorpha and subsequent sequence divergence and lineage-specific duplication events within the Lygaeidae (*Oncopeltus*) and Pentatominae (*Halyomorpha* and *Euschistus*). The phylogeny was constructed at phylogeny.fr with default pipeline settings, permitting gaps in the MUSCLE alignment and constructing the tree with PhyML [132]. All nodes have >70% support; branch length unit is substitutions per site; log-likelihood : -4598.93217. For the bacterial and *Halyomorpha* (Hhal) proteins, species and GenBank accession numbers are as indicated. The *Oncopeltus* protein IDs (OFAS) refer to the OGS v1.2 for this species [5]. For *Euschistus*, the tBLASTn hits are indicated by their position within assembly contig RCWM01000015.1 (contig00015).

**Figure S7. Phylogenetic tree of the OR family.** The tree was rooted with the highly conserved and basal Orco proteins. An asterisk indicates likely gene losses from *Halyomorpha*. Protein names and the branches leading to them are colored blue for *Halyomorpha*, orange for *Oncopeltus*, brown for *Rhodnius*, and red for *Cimex*. A suffix of P after the protein number indicates a pseudogene, while alternatively-spliced ORs are indicated by lower case letters after the protein number. Support for nodes is the approximate Likelihood Ratio Test value from PhyML v3.0, shown as size of a circle scaled from 0-1. The scale bar is substitutions per site.

**Figure S8. Phylogenetic tree of the GR family.** The tree was rooted with the conserved sugar and carbon dioxide receptor subfamilies. These two subfamilies, and the fructose receptor subfamily, are highlighted by colored background wedges, and representative members from *D. melanogaster*, *Apis mellifera*, *Tribolium castaneum*, and *Acyrthosiphon pisum* are included for comparison in these three subfamilies. Other features as in Additional file 1: Figure S7.

**Figure S9. Phylogenetic tree of the IR family.** The tree was rooted with the conserved co-receptor Ir8a and 25a lineages, which closely resemble the ionotropic glutamate receptors from which these variant Ionotropic Receptors evolved. The entire *D. melanogaster* IR repertoire was included for comparison. Other features as in Additional file 1: Figure S7, except that lower case suffixes do not indicate alternative-splicing, but rather either orthology with particular *Drosophila* IRs, or the Ir41 and 75 series of genes. Colored wedges highlight lineages clearly related to *Drosophila* IRs.

**Figure S10: Heteropteran global opsin gene tree.** Species abbreviations: *Acyrthosiphon pisum* = Apis, *Apis mellifera* = Amel, *Bemisia tabaci* = Btab, *Cimex lectularius* = Clec, *Diaphorina citri* = Dcit, *Diuraphis noxia* = Dnox, *Clastoptera arizonana* = Cari, *Gerris buenoi* = Gbue, *Halyomorpha halys* = Hhal, *Lygus hesperus* = Lhes, *Mezira granulata* = Mgra, *Musca domestica* = Mdom, *Nephotettix cincticeps*﻿ = Ncin, *Nilaparvata lugens* = Nlug, *Oncopeltus fasciatus* = Ofas, *Pachypsylla celtidismamma* = Pcel, *Pachypsylla venusta* = Pven, *Tribolium castaneum* = Tcas, *Triatoma infestans* = Tinf.

**Figure S11. Array of β-esterase genes.** A section of scaffold NW_014466677.1 harboring an array of eleven β-esterase genes, shown in grey. Note that gene model XM_014420119.1 appears to correspond to an unrelated lipase gene.

**Figure S12. Distribution of transcription factor families across insect genomes.** Heatmap depicting the abundance of transcription factor (TF) families across a collection of insect genomes. Each entry indicates the number of TF genes for the given family in the given genome, based on presence of DNA binding domains. Color key is depicted at the top (light blue means the TF family is completely absent) – note log (base 2) scale. Species were hierarchically clustered using average linkage clustering. *H. Halys* is boxed.

**Figure S13. Nanos amino acid sequence alignments from different species.** Alignment of partial nanos sequences from *Drosophila melanogaster, Aedes aegypti, Oncopeltus fasciatus, Schistocerca Americana, Tribolium castaneum, Apis mellifera* and *Parhyale hawaiensis.* Blacklines indicate two CCHC metal-binding domains.

**Figure S14. Location of pair-rule gene orthologs in the *H. halys* genome.** Complete sequences of most orthologs were found on the same scaffold, with the exception of *opa*, which necessitated merging sequences from two scaffolds. o*dd* and *run* share scaffolds with related genes *sob* and *lz*, respectively. Blue boxes mark exons, gray boxes signify UTR, arrows indicate the direction of transcription, dark black lines show introns, and thin black lines indicate intergenic regions. Only exons have been drawn to scale.

**Figure S15. *H. halys odd*-family genes.** Alignment of four tandem zinc fingers common to *Hhal-odd-2, -bowl, -sob,* and *–odd-1. Hhal-odd-1* exhibits higher sequence similarity with *Hhal-sob* and *–bowl* than does *Hhal-odd-2*.

**Figure S16. Alignment of Wnt family domain proteins.** Wingless in *T. castaneum* (red flour beetle, EFA04660), *D. melanogaster* (common fruit fly, NP_523502), N. lugens (brown planthopper, XP_022196814), *H. halys*, and *C. lectularis* (bed bug, XP_014249666).

**Figure S17. Engrailed and Invected are shared among diverse insects.** Alignment of Engrailed-family protein sequence in *D. melanogaster*, *T. castaneum*, *O. fasciatus*, *Thermobia domestica* (firebrat), and *H. halys*. Boxes indicate conserved regions.

**Figure S18: Phylogenetic analysis of hemipteran genes named “NR2E1” reveals that they are orthologous to NR2E6.** Sequences were selected based on the top hits to at tBLASTn search using the *Apis mellifera* NR2E6 amino acid sequence as a query. Sequences are named according to their current description in Genbank or FlyBase, not based on orthology to known genes. Nuclear receptor orthology was established using a MrBayes model (JTT plus Gamma), 2 runs, 100,000 generations, sampling frequency of 10, in TOPALi v2.5 [133]. Branches are supported by posterior probability. Alignment of all full-length represented proteins was generated by MUSCLE [134]. Accession numbers or FlyBase IDs of sequences used: *Amel*NR2E1 (XP_001121187.2), *Amel*NR2E6 (XM_016915404), *Amel*PNR (XP_396999.4), *Amel*Usp (XP_006561616.1), *Apis*PNR (XP_001948870.2), *Btab*NR2E1 (XP_018910426.1), *Clec*NR2E1 (XP_014250712.1), *Dcit*NR2E1 (XP_017299463.1), *Dmel*HR51 (FBgn0034012), DmelSvp (FBpp0082034), *Dmel*Tll (FBpp0085071), *Dmel*Usp (FBpp0070332), *Hhal*NR2E1 (XP_014290345.1),*Tcas*NR2E6 (XP_008194202.1), *Tcas*PNR (XM_001813960), *Tcas*Svp (XP_967537.1), *Tcas*Tll (NP_001034502.1), *Tjap*PNR (AID52848.1), *Tjap*Svp (AID52851.1).
