# Supplementary figures and images for: Brown marmorated stink bug, Halyomorpha halys (Stål), genome: putative underpinnings of polyphagy, insecticide resistance potential and biology of a top worldwide pest
Source: BMC Genomics. 2020 Mar 14;21:227. doi: 10.1186/s12864-020-6510-7 (PMC7071726; doi:10.1186/s12864-020-6510-7)

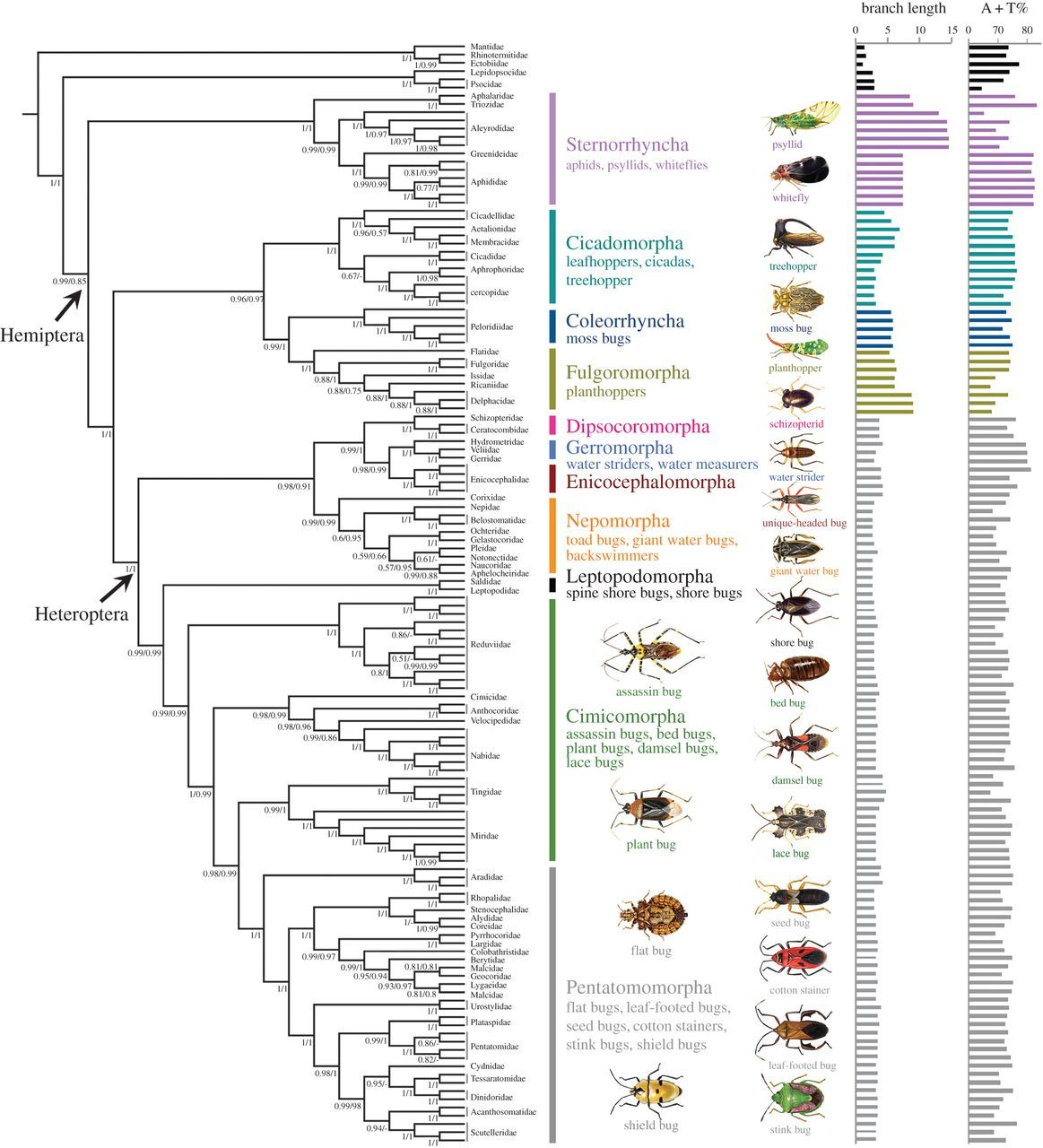

Supplement: Supplementary file 1 — Additional file 1: Main Supplementary Information text file, including Tables S1-S17 and Figures S1-S18. Table S1. Sequencing, assembly, annotation statistics and accession numbers. Table S2. OrthoDB v10 comparison of five species for ortholog presence and copy-number in Hemiptera-level orthogroups. Table S3. Scaffolds present in the H. halys assembly (accession GCA_000696795.1) that may originate from contaminant sources. Table S4. Counts of repetitive DNA elements encountered in the H. halys genome assembly. Table S5. H. halys predicted protein products associated with the RNAi pathway. Table S6. Positional information for the annotated homeobox genes. Table S7. Nuclear receptors of H. halys. Table S8. Listing of candidate Y-linked genes. Table S9. Number of genes identified as putative cuticle proteins per family in the genome of H. halys. Table S10. Number of genes identified as putative cuticle proteins per species in the genomes of several insect orders. Table S11. Clusters of genes coding for cuticle proteins in the genome of H. halys. Table S12. Odorant-binding protein genes and pseudogenes (Ψ) annotated in the genome of H. halys. Table S13. Primer sequences used to validate the HhalOBP gene annotations. Table S14. Correspondences between H. halys predicted protein identifiers and cathepsin labels. Table S15. A total of 64 salivary effector proteins were identified in the H. halys genome. Table S16. A select subset of 15 H. halys salivary effector proteins having variable expression levels between nymphal and adult stages (up- or down-regulation). Table S17. Gene expression data for H. halys glutathione S-transferase genes. Figure S1. Phylogenetic organization of the Hemiptera. Figure S2. Ortholog distributions among hemipterans. Figure S3. Genome assembly quality control. Figure S4. Hox and Iro-C cluster gene loci. Figure S5. Halyomorpha mannosidase expansion. Figure S6. Maximum likelihood phylogenetic tree of selected mannosidase proteins from three bacter [file 12864_2020_6510_MOESM1_ESM.zip › 12864_2020_6510_MOESM1_ESM/Fig_S01__HEMIPHYL.png]

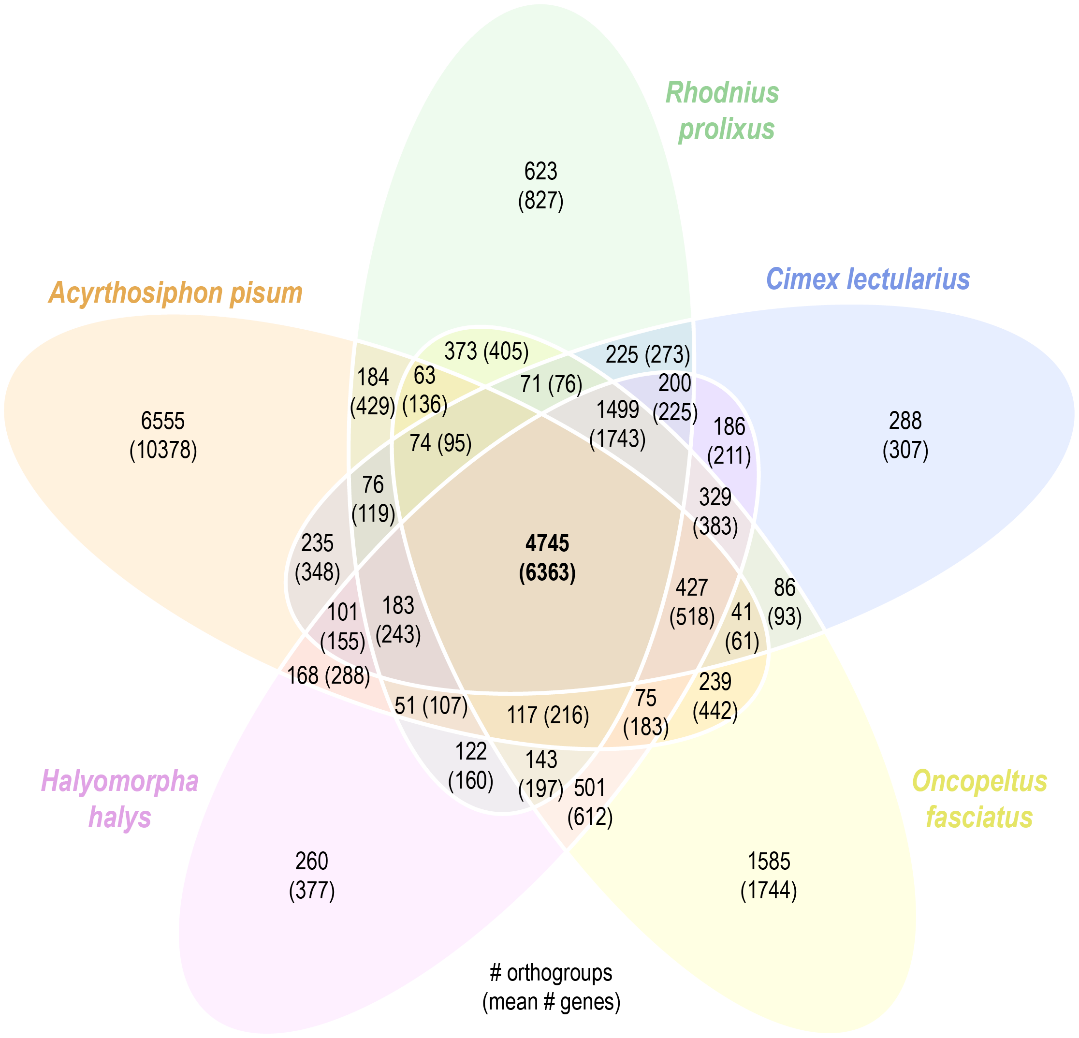

Supplement: Supplementary file 1 — Additional file 1: Main Supplementary Information text file, including Tables S1-S17 and Figures S1-S18. Table S1. Sequencing, assembly, annotation statistics and accession numbers. Table S2. OrthoDB v10 comparison of five species for ortholog presence and copy-number in Hemiptera-level orthogroups. Table S3. Scaffolds present in the H. halys assembly (accession GCA_000696795.1) that may originate from contaminant sources. Table S4. Counts of repetitive DNA elements encountered in the H. halys genome assembly. Table S5. H. halys predicted protein products associated with the RNAi pathway. Table S6. Positional information for the annotated homeobox genes. Table S7. Nuclear receptors of H. halys. Table S8. Listing of candidate Y-linked genes. Table S9. Number of genes identified as putative cuticle proteins per family in the genome of H. halys. Table S10. Number of genes identified as putative cuticle proteins per species in the genomes of several insect orders. Table S11. Clusters of genes coding for cuticle proteins in the genome of H. halys. Table S12. Odorant-binding protein genes and pseudogenes (Ψ) annotated in the genome of H. halys. Table S13. Primer sequences used to validate the HhalOBP gene annotations. Table S14. Correspondences between H. halys predicted protein identifiers and cathepsin labels. Table S15. A total of 64 salivary effector proteins were identified in the H. halys genome. Table S16. A select subset of 15 H. halys salivary effector proteins having variable expression levels between nymphal and adult stages (up- or down-regulation). Table S17. Gene expression data for H. halys glutathione S-transferase genes. Figure S1. Phylogenetic organization of the Hemiptera. Figure S2. Ortholog distributions among hemipterans. Figure S3. Genome assembly quality control. Figure S4. Hox and Iro-C cluster gene loci. Figure S5. Halyomorpha mannosidase expansion. Figure S6. Maximum likelihood phylogenetic tree of selected mannosidase proteins from three bacter [file 12864_2020_6510_MOESM1_ESM.zip › 12864_2020_6510_MOESM1_ESM/Fig_S02__ORTHO.png]

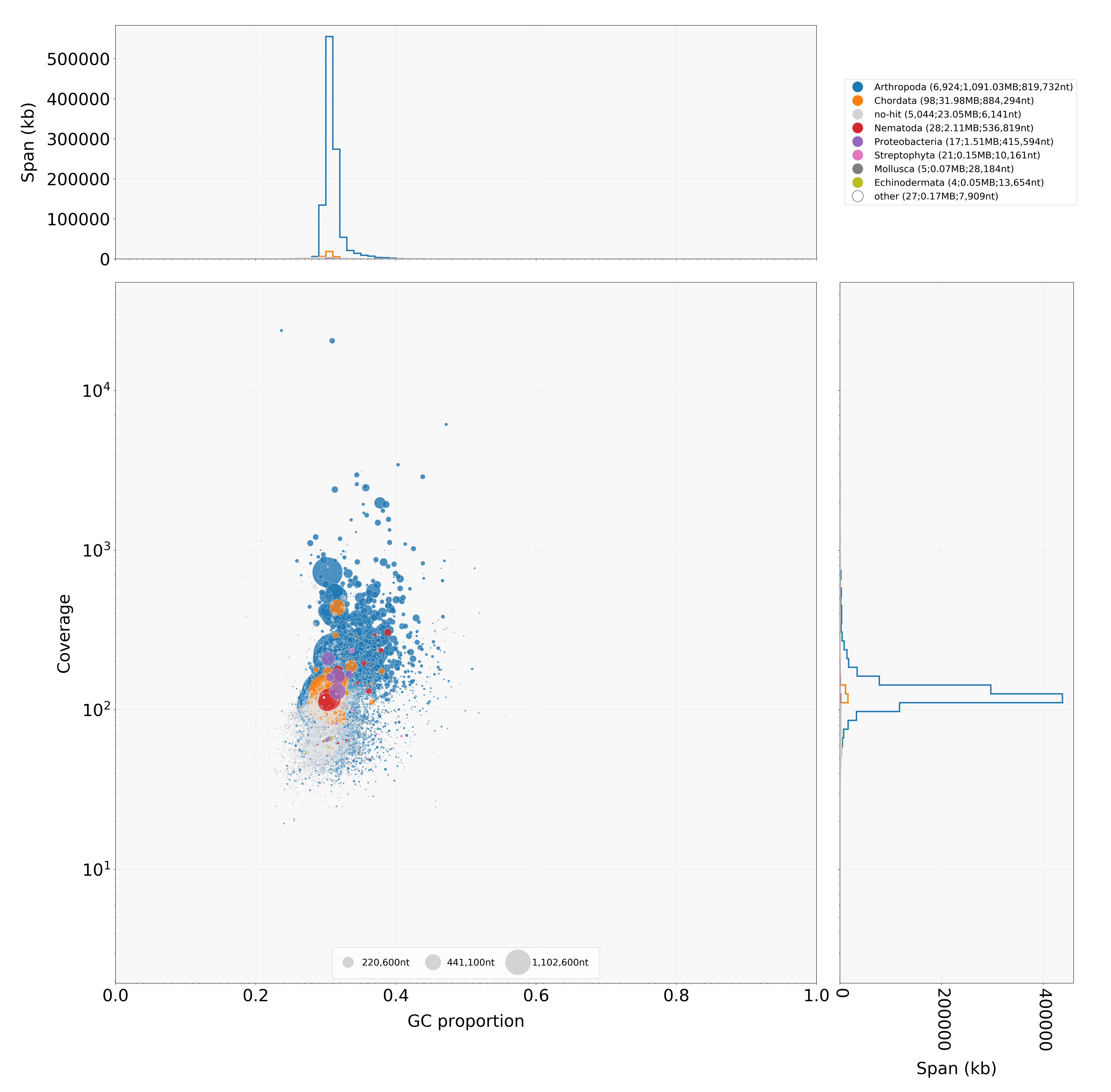

Supplement: Supplementary file 1 — Additional file 1: Main Supplementary Information text file, including Tables S1-S17 and Figures S1-S18. Table S1. Sequencing, assembly, annotation statistics and accession numbers. Table S2. OrthoDB v10 comparison of five species for ortholog presence and copy-number in Hemiptera-level orthogroups. Table S3. Scaffolds present in the H. halys assembly (accession GCA_000696795.1) that may originate from contaminant sources. Table S4. Counts of repetitive DNA elements encountered in the H. halys genome assembly. Table S5. H. halys predicted protein products associated with the RNAi pathway. Table S6. Positional information for the annotated homeobox genes. Table S7. Nuclear receptors of H. halys. Table S8. Listing of candidate Y-linked genes. Table S9. Number of genes identified as putative cuticle proteins per family in the genome of H. halys. Table S10. Number of genes identified as putative cuticle proteins per species in the genomes of several insect orders. Table S11. Clusters of genes coding for cuticle proteins in the genome of H. halys. Table S12. Odorant-binding protein genes and pseudogenes (Ψ) annotated in the genome of H. halys. Table S13. Primer sequences used to validate the HhalOBP gene annotations. Table S14. Correspondences between H. halys predicted protein identifiers and cathepsin labels. Table S15. A total of 64 salivary effector proteins were identified in the H. halys genome. Table S16. A select subset of 15 H. halys salivary effector proteins having variable expression levels between nymphal and adult stages (up- or down-regulation). Table S17. Gene expression data for H. halys glutathione S-transferase genes. Figure S1. Phylogenetic organization of the Hemiptera. Figure S2. Ortholog distributions among hemipterans. Figure S3. Genome assembly quality control. Figure S4. Hox and Iro-C cluster gene loci. Figure S5. Halyomorpha mannosidase expansion. Figure S6. Maximum likelihood phylogenetic tree of selected mannosidase proteins from three bacter [file 12864_2020_6510_MOESM1_ESM.zip › 12864_2020_6510_MOESM1_ESM/Fig_S03__ASMQC.png]

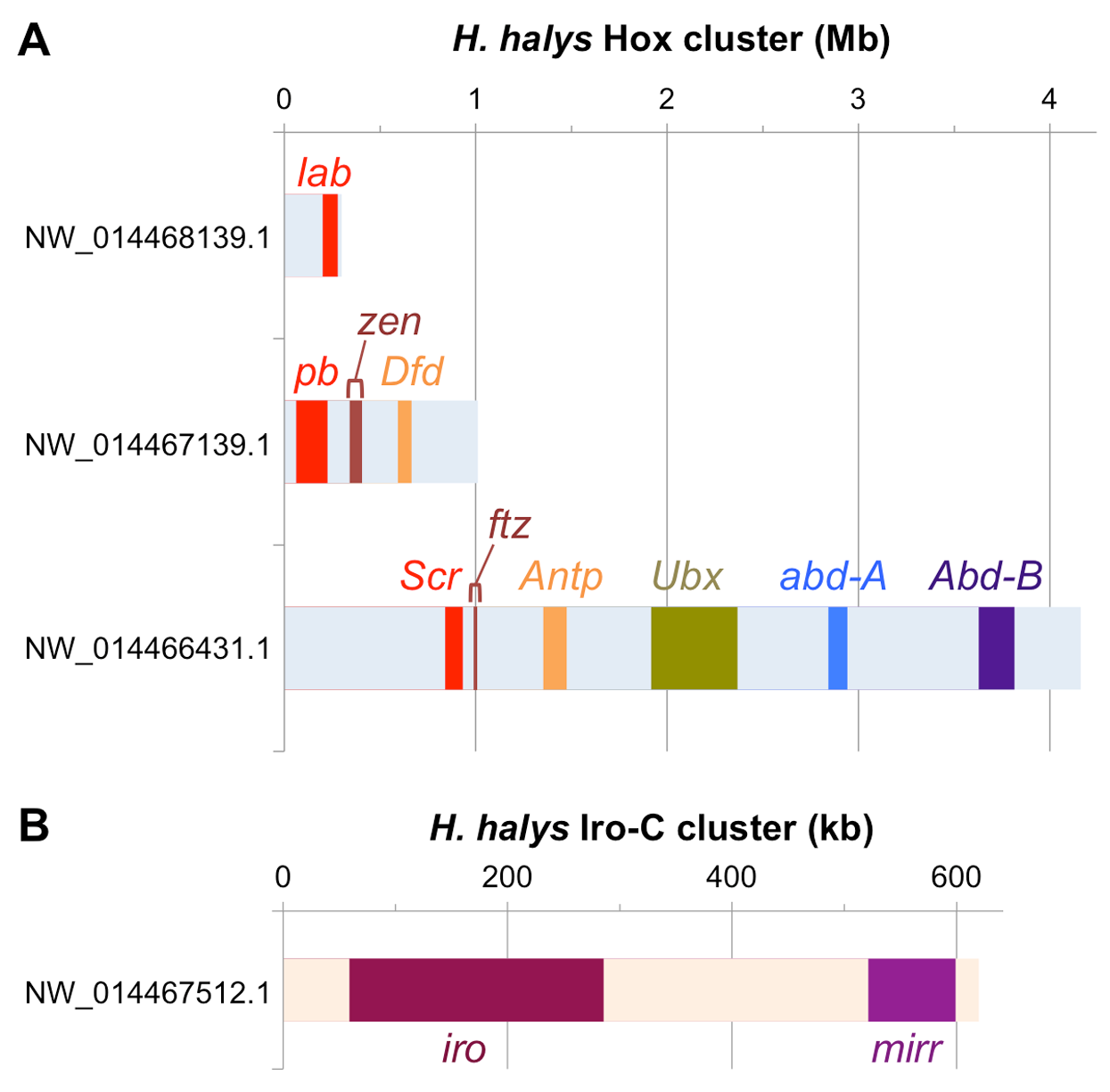

Supplement: Supplementary file 1 — Additional file 1: Main Supplementary Information text file, including Tables S1-S17 and Figures S1-S18. Table S1. Sequencing, assembly, annotation statistics and accession numbers. Table S2. OrthoDB v10 comparison of five species for ortholog presence and copy-number in Hemiptera-level orthogroups. Table S3. Scaffolds present in the H. halys assembly (accession GCA_000696795.1) that may originate from contaminant sources. Table S4. Counts of repetitive DNA elements encountered in the H. halys genome assembly. Table S5. H. halys predicted protein products associated with the RNAi pathway. Table S6. Positional information for the annotated homeobox genes. Table S7. Nuclear receptors of H. halys. Table S8. Listing of candidate Y-linked genes. Table S9. Number of genes identified as putative cuticle proteins per family in the genome of H. halys. Table S10. Number of genes identified as putative cuticle proteins per species in the genomes of several insect orders. Table S11. Clusters of genes coding for cuticle proteins in the genome of H. halys. Table S12. Odorant-binding protein genes and pseudogenes (Ψ) annotated in the genome of H. halys. Table S13. Primer sequences used to validate the HhalOBP gene annotations. Table S14. Correspondences between H. halys predicted protein identifiers and cathepsin labels. Table S15. A total of 64 salivary effector proteins were identified in the H. halys genome. Table S16. A select subset of 15 H. halys salivary effector proteins having variable expression levels between nymphal and adult stages (up- or down-regulation). Table S17. Gene expression data for H. halys glutathione S-transferase genes. Figure S1. Phylogenetic organization of the Hemiptera. Figure S2. Ortholog distributions among hemipterans. Figure S3. Genome assembly quality control. Figure S4. Hox and Iro-C cluster gene loci. Figure S5. Halyomorpha mannosidase expansion. Figure S6. Maximum likelihood phylogenetic tree of selected mannosidase proteins from three bacter [file 12864_2020_6510_MOESM1_ESM.zip › 12864_2020_6510_MOESM1_ESM/Fig_S04__HOXIRO.png]

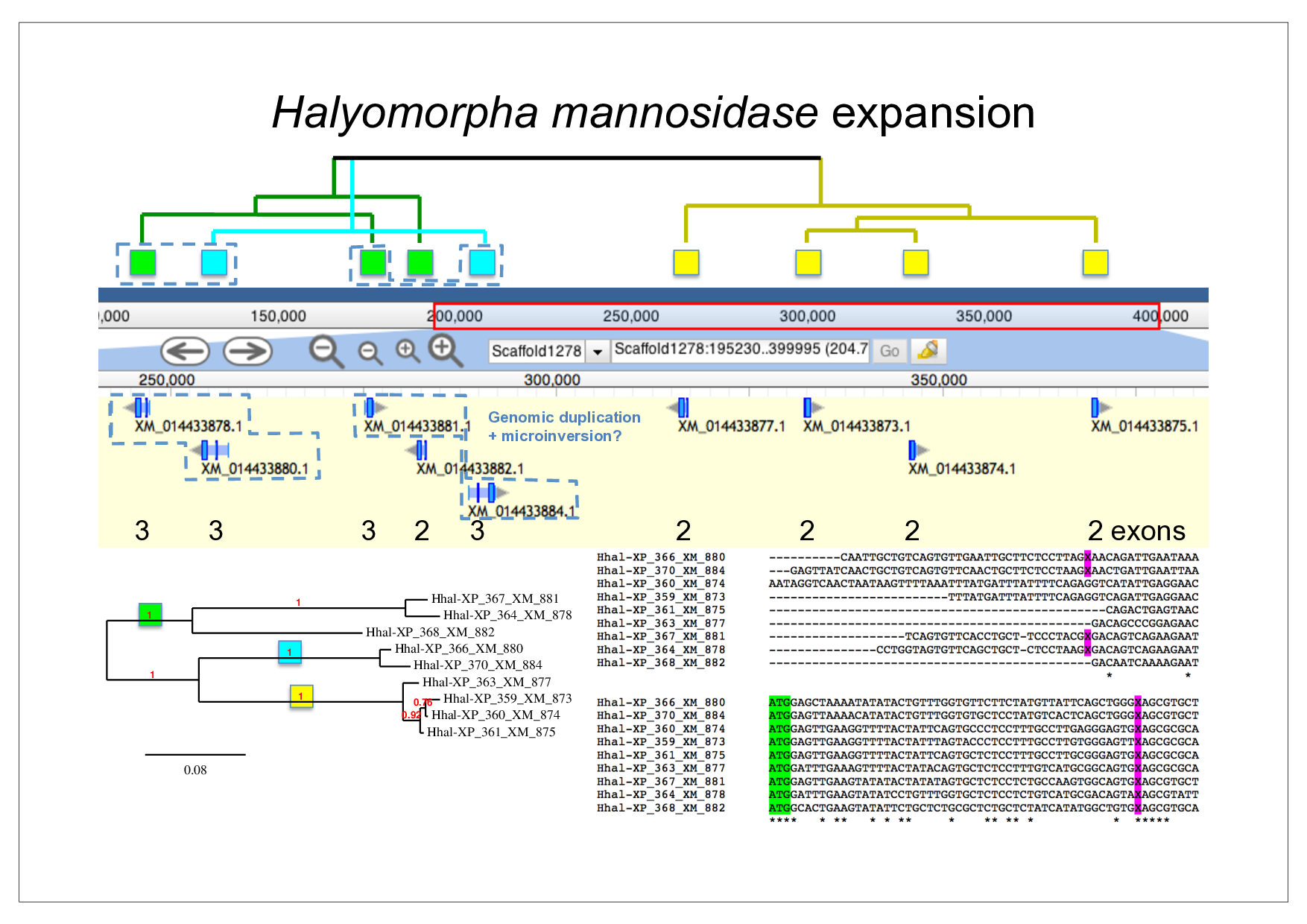

Supplement: Supplementary file 1 — Additional file 1: Main Supplementary Information text file, including Tables S1-S17 and Figures S1-S18. Table S1. Sequencing, assembly, annotation statistics and accession numbers. Table S2. OrthoDB v10 comparison of five species for ortholog presence and copy-number in Hemiptera-level orthogroups. Table S3. Scaffolds present in the H. halys assembly (accession GCA_000696795.1) that may originate from contaminant sources. Table S4. Counts of repetitive DNA elements encountered in the H. halys genome assembly. Table S5. H. halys predicted protein products associated with the RNAi pathway. Table S6. Positional information for the annotated homeobox genes. Table S7. Nuclear receptors of H. halys. Table S8. Listing of candidate Y-linked genes. Table S9. Number of genes identified as putative cuticle proteins per family in the genome of H. halys. Table S10. Number of genes identified as putative cuticle proteins per species in the genomes of several insect orders. Table S11. Clusters of genes coding for cuticle proteins in the genome of H. halys. Table S12. Odorant-binding protein genes and pseudogenes (Ψ) annotated in the genome of H. halys. Table S13. Primer sequences used to validate the HhalOBP gene annotations. Table S14. Correspondences between H. halys predicted protein identifiers and cathepsin labels. Table S15. A total of 64 salivary effector proteins were identified in the H. halys genome. Table S16. A select subset of 15 H. halys salivary effector proteins having variable expression levels between nymphal and adult stages (up- or down-regulation). Table S17. Gene expression data for H. halys glutathione S-transferase genes. Figure S1. Phylogenetic organization of the Hemiptera. Figure S2. Ortholog distributions among hemipterans. Figure S3. Genome assembly quality control. Figure S4. Hox and Iro-C cluster gene loci. Figure S5. Halyomorpha mannosidase expansion. Figure S6. Maximum likelihood phylogenetic tree of selected mannosidase proteins from three bacter [file 12864_2020_6510_MOESM1_ESM.zip › 12864_2020_6510_MOESM1_ESM/Fig_S05__MANNOEX.png]

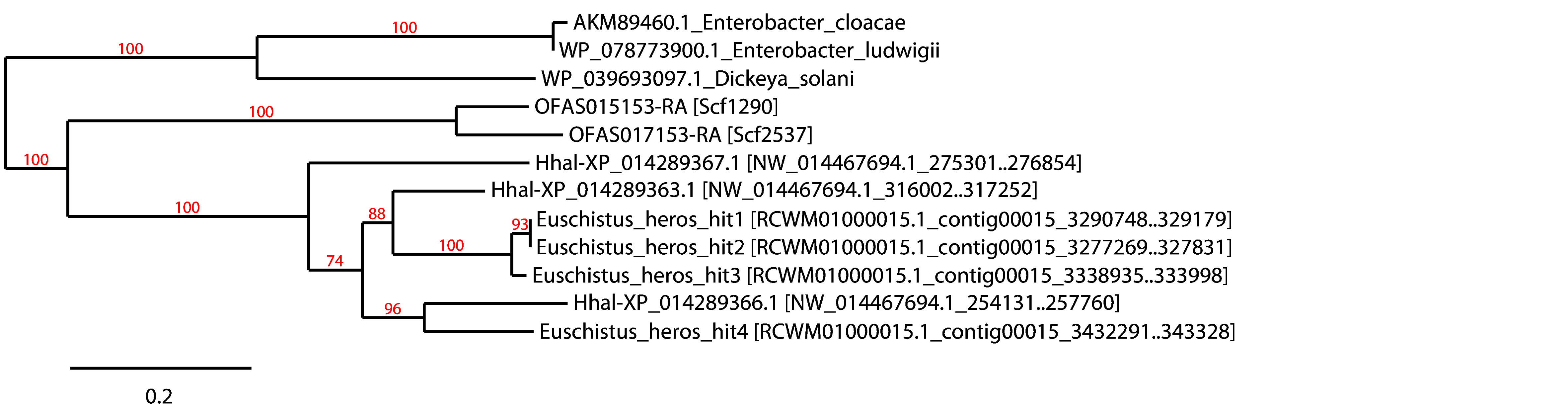

Supplement: Supplementary file 1 — Additional file 1: Main Supplementary Information text file, including Tables S1-S17 and Figures S1-S18. Table S1. Sequencing, assembly, annotation statistics and accession numbers. Table S2. OrthoDB v10 comparison of five species for ortholog presence and copy-number in Hemiptera-level orthogroups. Table S3. Scaffolds present in the H. halys assembly (accession GCA_000696795.1) that may originate from contaminant sources. Table S4. Counts of repetitive DNA elements encountered in the H. halys genome assembly. Table S5. H. halys predicted protein products associated with the RNAi pathway. Table S6. Positional information for the annotated homeobox genes. Table S7. Nuclear receptors of H. halys. Table S8. Listing of candidate Y-linked genes. Table S9. Number of genes identified as putative cuticle proteins per family in the genome of H. halys. Table S10. Number of genes identified as putative cuticle proteins per species in the genomes of several insect orders. Table S11. Clusters of genes coding for cuticle proteins in the genome of H. halys. Table S12. Odorant-binding protein genes and pseudogenes (Ψ) annotated in the genome of H. halys. Table S13. Primer sequences used to validate the HhalOBP gene annotations. Table S14. Correspondences between H. halys predicted protein identifiers and cathepsin labels. Table S15. A total of 64 salivary effector proteins were identified in the H. halys genome. Table S16. A select subset of 15 H. halys salivary effector proteins having variable expression levels between nymphal and adult stages (up- or down-regulation). Table S17. Gene expression data for H. halys glutathione S-transferase genes. Figure S1. Phylogenetic organization of the Hemiptera. Figure S2. Ortholog distributions among hemipterans. Figure S3. Genome assembly quality control. Figure S4. Hox and Iro-C cluster gene loci. Figure S5. Halyomorpha mannosidase expansion. Figure S6. Maximum likelihood phylogenetic tree of selected mannosidase proteins from three bacter [file 12864_2020_6510_MOESM1_ESM.zip › 12864_2020_6510_MOESM1_ESM/Fig_S06__MANNPHYLO.png]

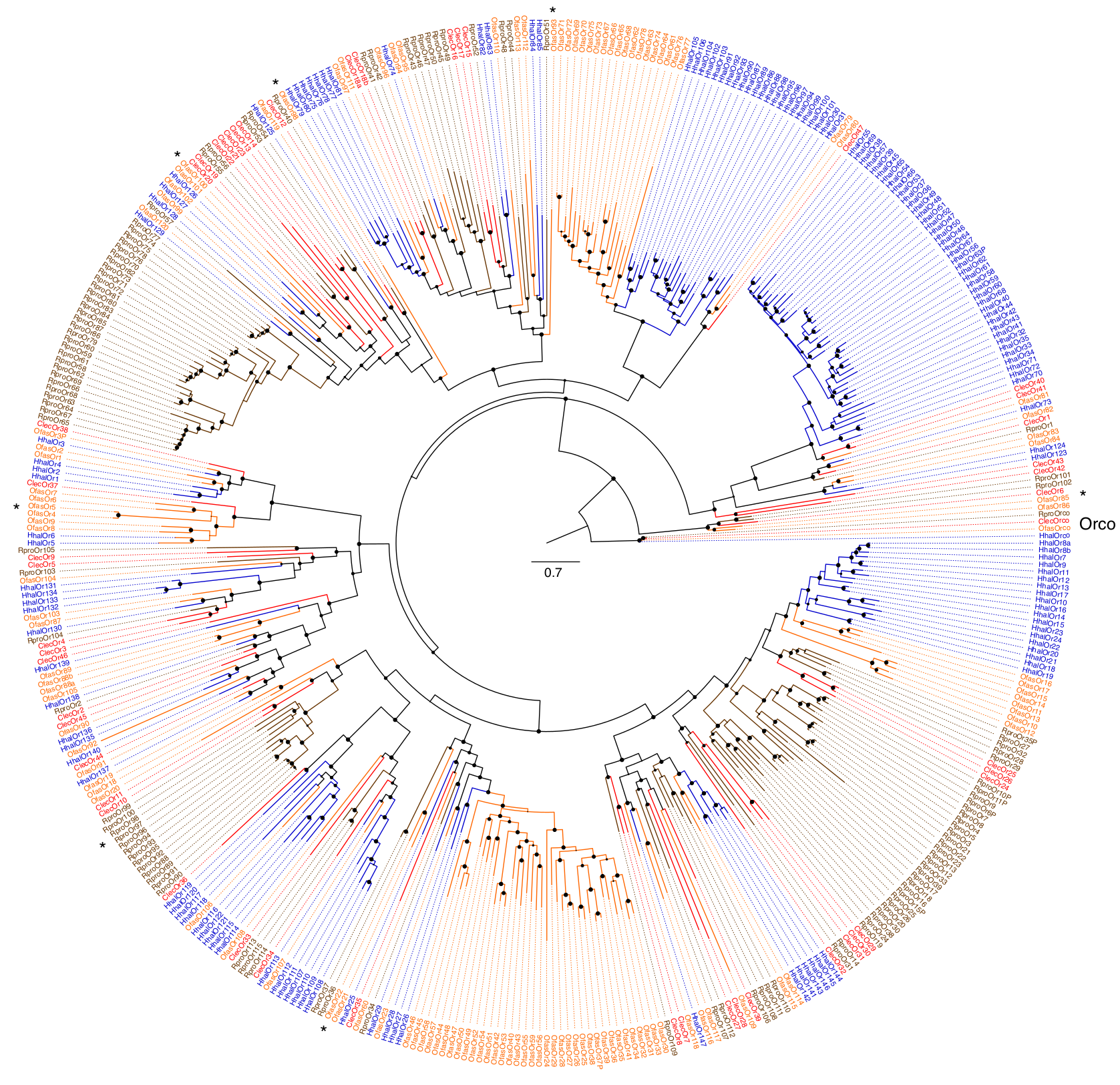

Supplement: Supplementary file 1 — Additional file 1: Main Supplementary Information text file, including Tables S1-S17 and Figures S1-S18. Table S1. Sequencing, assembly, annotation statistics and accession numbers. Table S2. OrthoDB v10 comparison of five species for ortholog presence and copy-number in Hemiptera-level orthogroups. Table S3. Scaffolds present in the H. halys assembly (accession GCA_000696795.1) that may originate from contaminant sources. Table S4. Counts of repetitive DNA elements encountered in the H. halys genome assembly. Table S5. H. halys predicted protein products associated with the RNAi pathway. Table S6. Positional information for the annotated homeobox genes. Table S7. Nuclear receptors of H. halys. Table S8. Listing of candidate Y-linked genes. Table S9. Number of genes identified as putative cuticle proteins per family in the genome of H. halys. Table S10. Number of genes identified as putative cuticle proteins per species in the genomes of several insect orders. Table S11. Clusters of genes coding for cuticle proteins in the genome of H. halys. Table S12. Odorant-binding protein genes and pseudogenes (Ψ) annotated in the genome of H. halys. Table S13. Primer sequences used to validate the HhalOBP gene annotations. Table S14. Correspondences between H. halys predicted protein identifiers and cathepsin labels. Table S15. A total of 64 salivary effector proteins were identified in the H. halys genome. Table S16. A select subset of 15 H. halys salivary effector proteins having variable expression levels between nymphal and adult stages (up- or down-regulation). Table S17. Gene expression data for H. halys glutathione S-transferase genes. Figure S1. Phylogenetic organization of the Hemiptera. Figure S2. Ortholog distributions among hemipterans. Figure S3. Genome assembly quality control. Figure S4. Hox and Iro-C cluster gene loci. Figure S5. Halyomorpha mannosidase expansion. Figure S6. Maximum likelihood phylogenetic tree of selected mannosidase proteins from three bacter [file 12864_2020_6510_MOESM1_ESM.zip › 12864_2020_6510_MOESM1_ESM/Fig_S07__ORPHY.png]

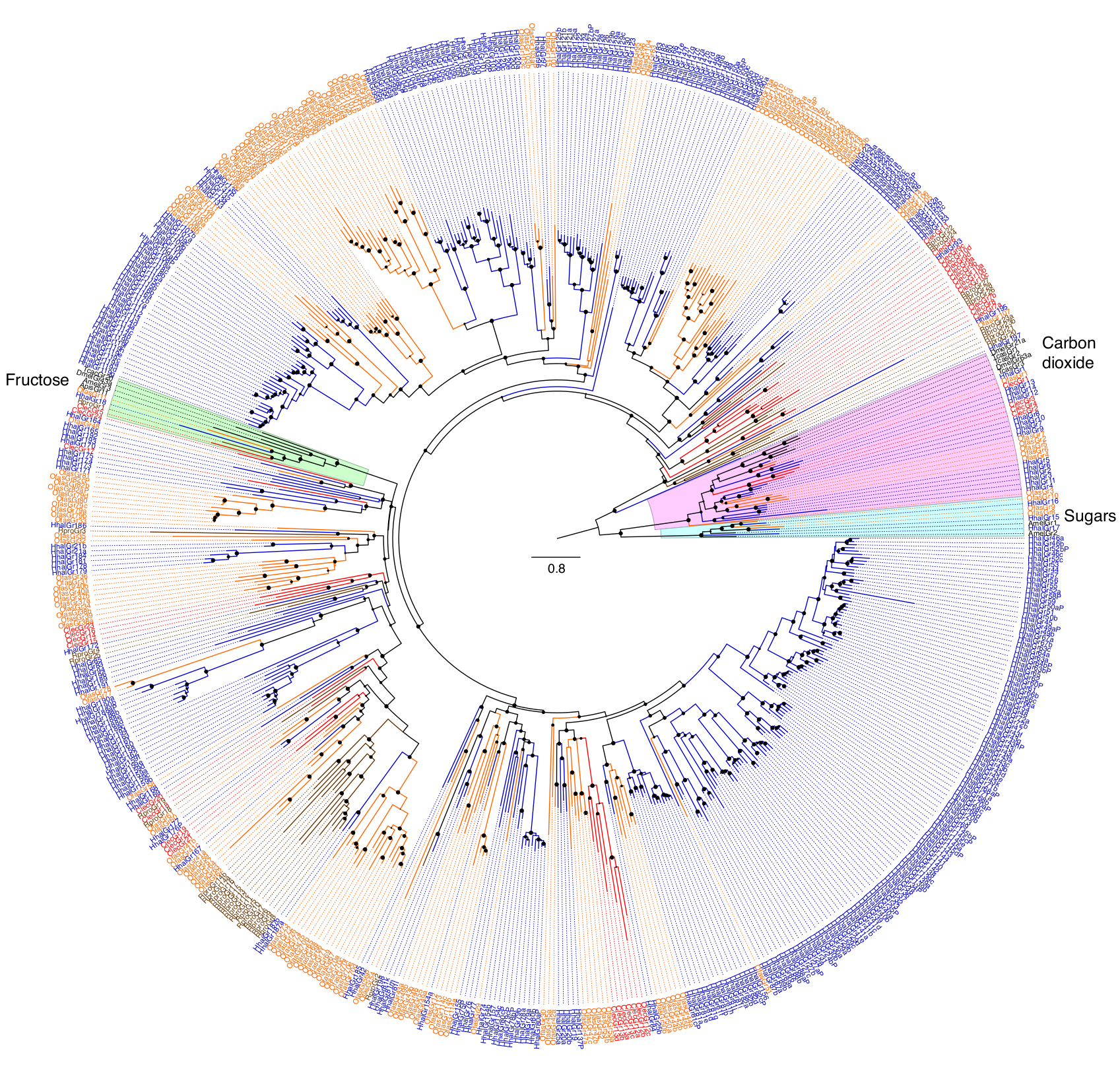

Supplement: Supplementary file 1 — Additional file 1: Main Supplementary Information text file, including Tables S1-S17 and Figures S1-S18. Table S1. Sequencing, assembly, annotation statistics and accession numbers. Table S2. OrthoDB v10 comparison of five species for ortholog presence and copy-number in Hemiptera-level orthogroups. Table S3. Scaffolds present in the H. halys assembly (accession GCA_000696795.1) that may originate from contaminant sources. Table S4. Counts of repetitive DNA elements encountered in the H. halys genome assembly. Table S5. H. halys predicted protein products associated with the RNAi pathway. Table S6. Positional information for the annotated homeobox genes. Table S7. Nuclear receptors of H. halys. Table S8. Listing of candidate Y-linked genes. Table S9. Number of genes identified as putative cuticle proteins per family in the genome of H. halys. Table S10. Number of genes identified as putative cuticle proteins per species in the genomes of several insect orders. Table S11. Clusters of genes coding for cuticle proteins in the genome of H. halys. Table S12. Odorant-binding protein genes and pseudogenes (Ψ) annotated in the genome of H. halys. Table S13. Primer sequences used to validate the HhalOBP gene annotations. Table S14. Correspondences between H. halys predicted protein identifiers and cathepsin labels. Table S15. A total of 64 salivary effector proteins were identified in the H. halys genome. Table S16. A select subset of 15 H. halys salivary effector proteins having variable expression levels between nymphal and adult stages (up- or down-regulation). Table S17. Gene expression data for H. halys glutathione S-transferase genes. Figure S1. Phylogenetic organization of the Hemiptera. Figure S2. Ortholog distributions among hemipterans. Figure S3. Genome assembly quality control. Figure S4. Hox and Iro-C cluster gene loci. Figure S5. Halyomorpha mannosidase expansion. Figure S6. Maximum likelihood phylogenetic tree of selected mannosidase proteins from three bacter [file 12864_2020_6510_MOESM1_ESM.zip › 12864_2020_6510_MOESM1_ESM/Fig_S08__GRPHY.png]

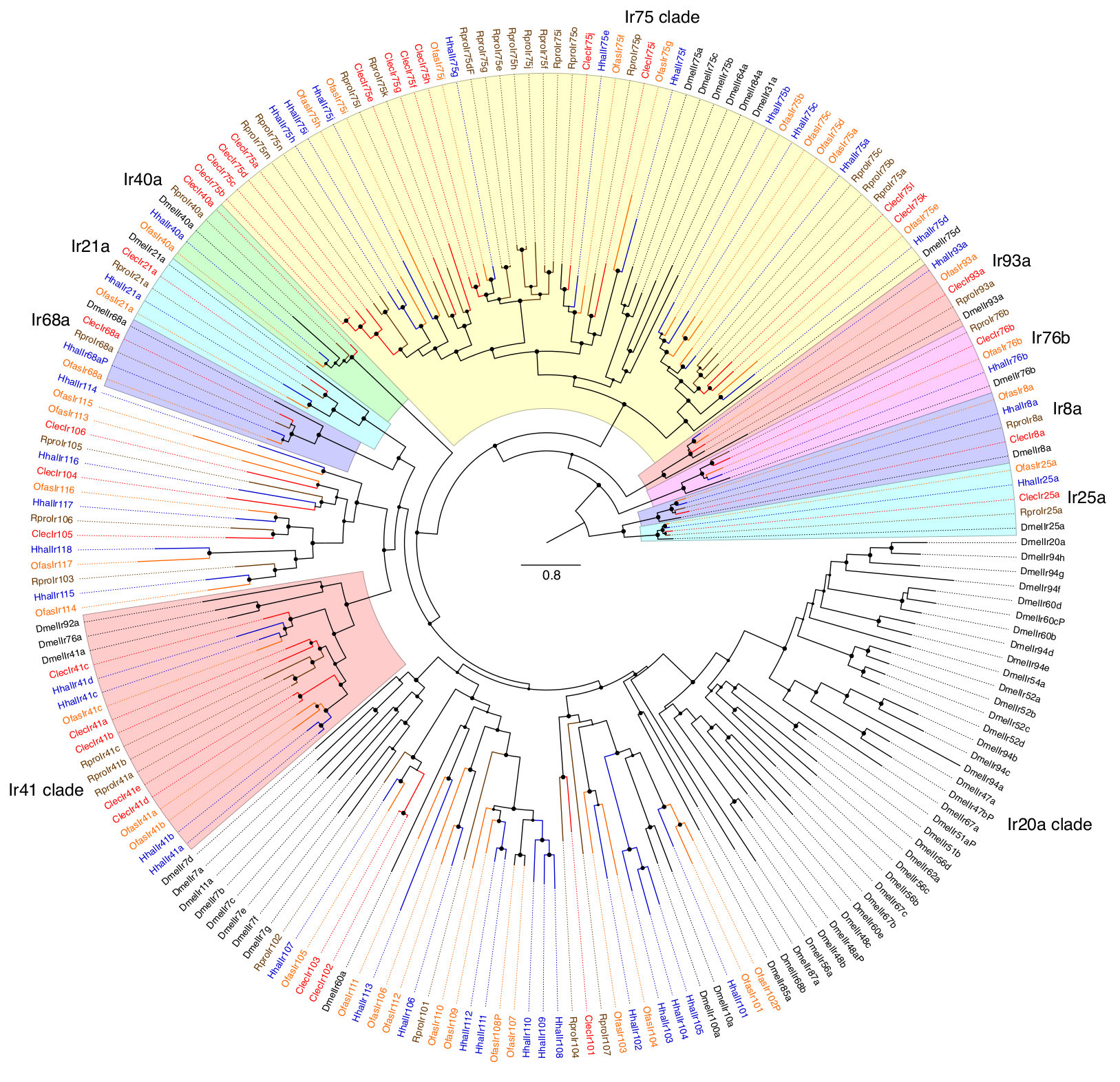

Supplement: Supplementary file 1 — Additional file 1: Main Supplementary Information text file, including Tables S1-S17 and Figures S1-S18. Table S1. Sequencing, assembly, annotation statistics and accession numbers. Table S2. OrthoDB v10 comparison of five species for ortholog presence and copy-number in Hemiptera-level orthogroups. Table S3. Scaffolds present in the H. halys assembly (accession GCA_000696795.1) that may originate from contaminant sources. Table S4. Counts of repetitive DNA elements encountered in the H. halys genome assembly. Table S5. H. halys predicted protein products associated with the RNAi pathway. Table S6. Positional information for the annotated homeobox genes. Table S7. Nuclear receptors of H. halys. Table S8. Listing of candidate Y-linked genes. Table S9. Number of genes identified as putative cuticle proteins per family in the genome of H. halys. Table S10. Number of genes identified as putative cuticle proteins per species in the genomes of several insect orders. Table S11. Clusters of genes coding for cuticle proteins in the genome of H. halys. Table S12. Odorant-binding protein genes and pseudogenes (Ψ) annotated in the genome of H. halys. Table S13. Primer sequences used to validate the HhalOBP gene annotations. Table S14. Correspondences between H. halys predicted protein identifiers and cathepsin labels. Table S15. A total of 64 salivary effector proteins were identified in the H. halys genome. Table S16. A select subset of 15 H. halys salivary effector proteins having variable expression levels between nymphal and adult stages (up- or down-regulation). Table S17. Gene expression data for H. halys glutathione S-transferase genes. Figure S1. Phylogenetic organization of the Hemiptera. Figure S2. Ortholog distributions among hemipterans. Figure S3. Genome assembly quality control. Figure S4. Hox and Iro-C cluster gene loci. Figure S5. Halyomorpha mannosidase expansion. Figure S6. Maximum likelihood phylogenetic tree of selected mannosidase proteins from three bacter [file 12864_2020_6510_MOESM1_ESM.zip › 12864_2020_6510_MOESM1_ESM/Fig_S09__IRPHY.png]

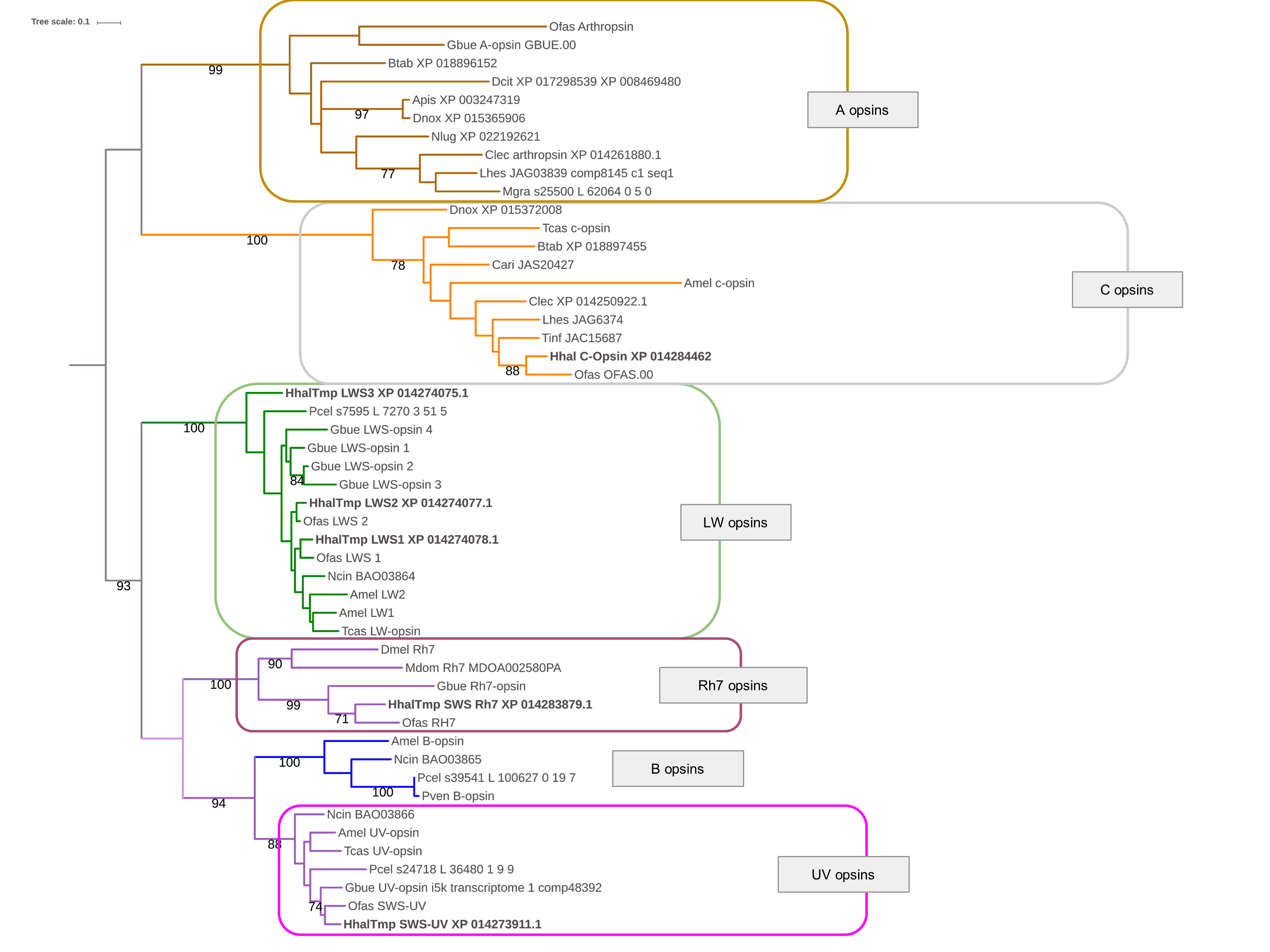

Supplement: Supplementary file 1 — Additional file 1: Main Supplementary Information text file, including Tables S1-S17 and Figures S1-S18. Table S1. Sequencing, assembly, annotation statistics and accession numbers. Table S2. OrthoDB v10 comparison of five species for ortholog presence and copy-number in Hemiptera-level orthogroups. Table S3. Scaffolds present in the H. halys assembly (accession GCA_000696795.1) that may originate from contaminant sources. Table S4. Counts of repetitive DNA elements encountered in the H. halys genome assembly. Table S5. H. halys predicted protein products associated with the RNAi pathway. Table S6. Positional information for the annotated homeobox genes. Table S7. Nuclear receptors of H. halys. Table S8. Listing of candidate Y-linked genes. Table S9. Number of genes identified as putative cuticle proteins per family in the genome of H. halys. Table S10. Number of genes identified as putative cuticle proteins per species in the genomes of several insect orders. Table S11. Clusters of genes coding for cuticle proteins in the genome of H. halys. Table S12. Odorant-binding protein genes and pseudogenes (Ψ) annotated in the genome of H. halys. Table S13. Primer sequences used to validate the HhalOBP gene annotations. Table S14. Correspondences between H. halys predicted protein identifiers and cathepsin labels. Table S15. A total of 64 salivary effector proteins were identified in the H. halys genome. Table S16. A select subset of 15 H. halys salivary effector proteins having variable expression levels between nymphal and adult stages (up- or down-regulation). Table S17. Gene expression data for H. halys glutathione S-transferase genes. Figure S1. Phylogenetic organization of the Hemiptera. Figure S2. Ortholog distributions among hemipterans. Figure S3. Genome assembly quality control. Figure S4. Hox and Iro-C cluster gene loci. Figure S5. Halyomorpha mannosidase expansion. Figure S6. Maximum likelihood phylogenetic tree of selected mannosidase proteins from three bacter [file 12864_2020_6510_MOESM1_ESM.zip › 12864_2020_6510_MOESM1_ESM/Fig_S10__GLOBOPTREE.png]

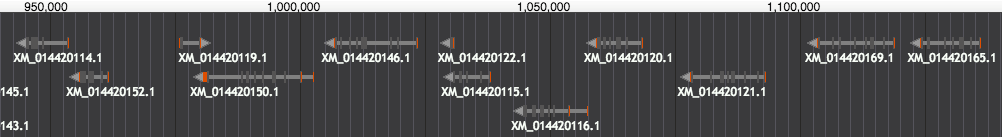

Supplement: Supplementary file 1 — Additional file 1: Main Supplementary Information text file, including Tables S1-S17 and Figures S1-S18. Table S1. Sequencing, assembly, annotation statistics and accession numbers. Table S2. OrthoDB v10 comparison of five species for ortholog presence and copy-number in Hemiptera-level orthogroups. Table S3. Scaffolds present in the H. halys assembly (accession GCA_000696795.1) that may originate from contaminant sources. Table S4. Counts of repetitive DNA elements encountered in the H. halys genome assembly. Table S5. H. halys predicted protein products associated with the RNAi pathway. Table S6. Positional information for the annotated homeobox genes. Table S7. Nuclear receptors of H. halys. Table S8. Listing of candidate Y-linked genes. Table S9. Number of genes identified as putative cuticle proteins per family in the genome of H. halys. Table S10. Number of genes identified as putative cuticle proteins per species in the genomes of several insect orders. Table S11. Clusters of genes coding for cuticle proteins in the genome of H. halys. Table S12. Odorant-binding protein genes and pseudogenes (Ψ) annotated in the genome of H. halys. Table S13. Primer sequences used to validate the HhalOBP gene annotations. Table S14. Correspondences between H. halys predicted protein identifiers and cathepsin labels. Table S15. A total of 64 salivary effector proteins were identified in the H. halys genome. Table S16. A select subset of 15 H. halys salivary effector proteins having variable expression levels between nymphal and adult stages (up- or down-regulation). Table S17. Gene expression data for H. halys glutathione S-transferase genes. Figure S1. Phylogenetic organization of the Hemiptera. Figure S2. Ortholog distributions among hemipterans. Figure S3. Genome assembly quality control. Figure S4. Hox and Iro-C cluster gene loci. Figure S5. Halyomorpha mannosidase expansion. Figure S6. Maximum likelihood phylogenetic tree of selected mannosidase proteins from three bacter [file 12864_2020_6510_MOESM1_ESM.zip › 12864_2020_6510_MOESM1_ESM/Fig_S11__COEJBROWSE.png]

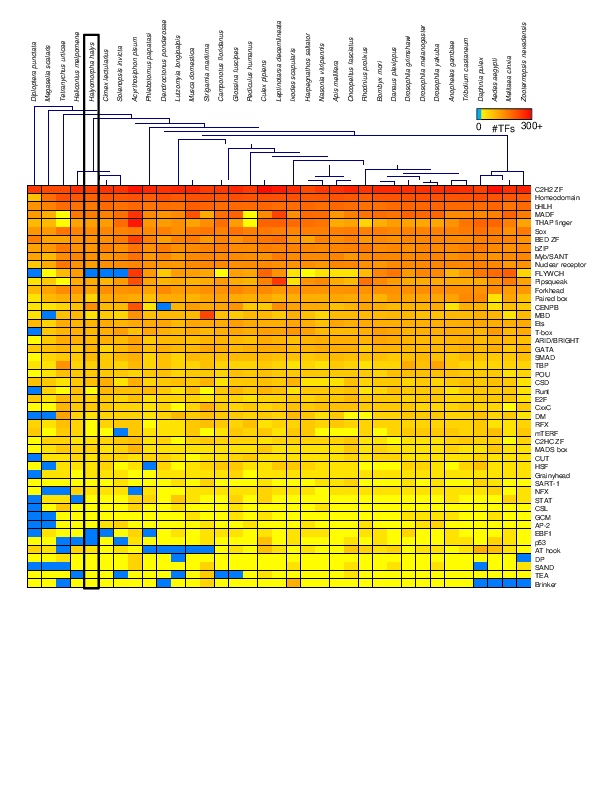

Supplement: Supplementary file 1 — Additional file 1: Main Supplementary Information text file, including Tables S1-S17 and Figures S1-S18. Table S1. Sequencing, assembly, annotation statistics and accession numbers. Table S2. OrthoDB v10 comparison of five species for ortholog presence and copy-number in Hemiptera-level orthogroups. Table S3. Scaffolds present in the H. halys assembly (accession GCA_000696795.1) that may originate from contaminant sources. Table S4. Counts of repetitive DNA elements encountered in the H. halys genome assembly. Table S5. H. halys predicted protein products associated with the RNAi pathway. Table S6. Positional information for the annotated homeobox genes. Table S7. Nuclear receptors of H. halys. Table S8. Listing of candidate Y-linked genes. Table S9. Number of genes identified as putative cuticle proteins per family in the genome of H. halys. Table S10. Number of genes identified as putative cuticle proteins per species in the genomes of several insect orders. Table S11. Clusters of genes coding for cuticle proteins in the genome of H. halys. Table S12. Odorant-binding protein genes and pseudogenes (Ψ) annotated in the genome of H. halys. Table S13. Primer sequences used to validate the HhalOBP gene annotations. Table S14. Correspondences between H. halys predicted protein identifiers and cathepsin labels. Table S15. A total of 64 salivary effector proteins were identified in the H. halys genome. Table S16. A select subset of 15 H. halys salivary effector proteins having variable expression levels between nymphal and adult stages (up- or down-regulation). Table S17. Gene expression data for H. halys glutathione S-transferase genes. Figure S1. Phylogenetic organization of the Hemiptera. Figure S2. Ortholog distributions among hemipterans. Figure S3. Genome assembly quality control. Figure S4. Hox and Iro-C cluster gene loci. Figure S5. Halyomorpha mannosidase expansion. Figure S6. Maximum likelihood phylogenetic tree of selected mannosidase proteins from three bacter [file 12864_2020_6510_MOESM1_ESM.zip › 12864_2020_6510_MOESM1_ESM/Fig_S12__TRANSFACFIG.png]

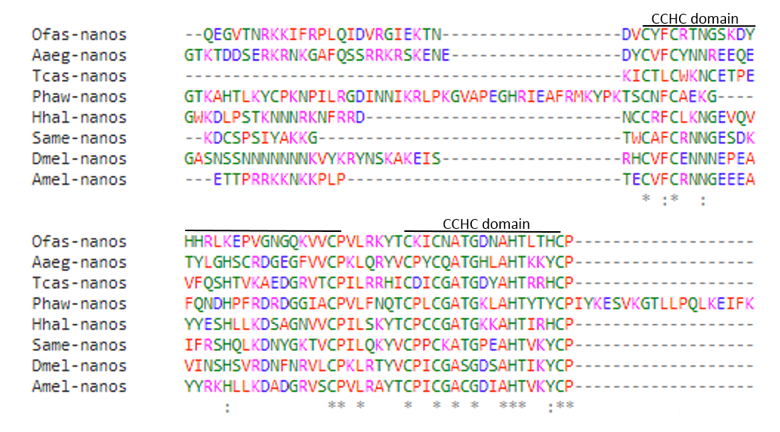

Supplement: Supplementary file 1 — Additional file 1: Main Supplementary Information text file, including Tables S1-S17 and Figures S1-S18. Table S1. Sequencing, assembly, annotation statistics and accession numbers. Table S2. OrthoDB v10 comparison of five species for ortholog presence and copy-number in Hemiptera-level orthogroups. Table S3. Scaffolds present in the H. halys assembly (accession GCA_000696795.1) that may originate from contaminant sources. Table S4. Counts of repetitive DNA elements encountered in the H. halys genome assembly. Table S5. H. halys predicted protein products associated with the RNAi pathway. Table S6. Positional information for the annotated homeobox genes. Table S7. Nuclear receptors of H. halys. Table S8. Listing of candidate Y-linked genes. Table S9. Number of genes identified as putative cuticle proteins per family in the genome of H. halys. Table S10. Number of genes identified as putative cuticle proteins per species in the genomes of several insect orders. Table S11. Clusters of genes coding for cuticle proteins in the genome of H. halys. Table S12. Odorant-binding protein genes and pseudogenes (Ψ) annotated in the genome of H. halys. Table S13. Primer sequences used to validate the HhalOBP gene annotations. Table S14. Correspondences between H. halys predicted protein identifiers and cathepsin labels. Table S15. A total of 64 salivary effector proteins were identified in the H. halys genome. Table S16. A select subset of 15 H. halys salivary effector proteins having variable expression levels between nymphal and adult stages (up- or down-regulation). Table S17. Gene expression data for H. halys glutathione S-transferase genes. Figure S1. Phylogenetic organization of the Hemiptera. Figure S2. Ortholog distributions among hemipterans. Figure S3. Genome assembly quality control. Figure S4. Hox and Iro-C cluster gene loci. Figure S5. Halyomorpha mannosidase expansion. Figure S6. Maximum likelihood phylogenetic tree of selected mannosidase proteins from three bacter [file 12864_2020_6510_MOESM1_ESM.zip › 12864_2020_6510_MOESM1_ESM/Fig_S13__CCHC.png]

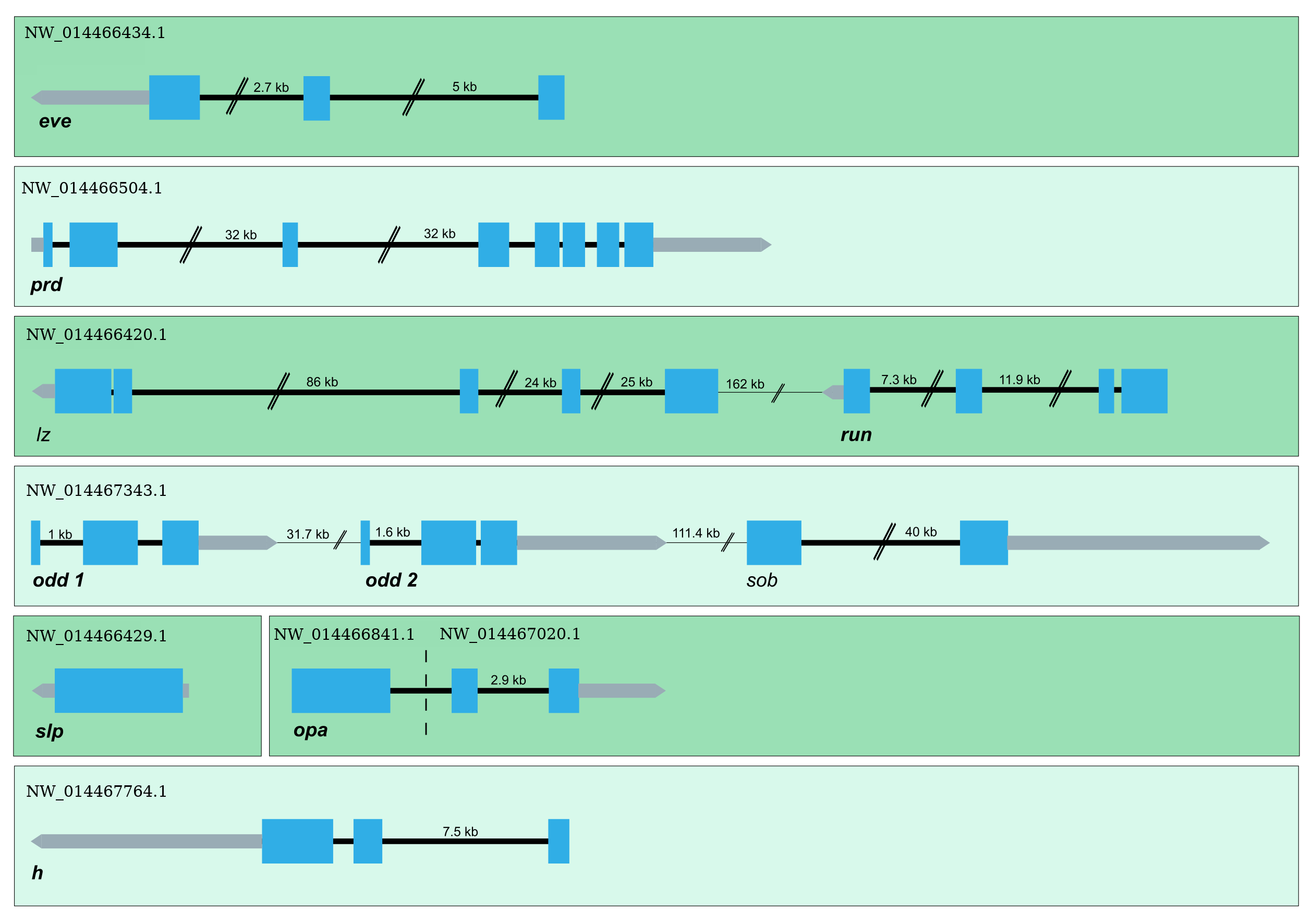

Supplement: Supplementary file 1 — Additional file 1: Main Supplementary Information text file, including Tables S1-S17 and Figures S1-S18. Table S1. Sequencing, assembly, annotation statistics and accession numbers. Table S2. OrthoDB v10 comparison of five species for ortholog presence and copy-number in Hemiptera-level orthogroups. Table S3. Scaffolds present in the H. halys assembly (accession GCA_000696795.1) that may originate from contaminant sources. Table S4. Counts of repetitive DNA elements encountered in the H. halys genome assembly. Table S5. H. halys predicted protein products associated with the RNAi pathway. Table S6. Positional information for the annotated homeobox genes. Table S7. Nuclear receptors of H. halys. Table S8. Listing of candidate Y-linked genes. Table S9. Number of genes identified as putative cuticle proteins per family in the genome of H. halys. Table S10. Number of genes identified as putative cuticle proteins per species in the genomes of several insect orders. Table S11. Clusters of genes coding for cuticle proteins in the genome of H. halys. Table S12. Odorant-binding protein genes and pseudogenes (Ψ) annotated in the genome of H. halys. Table S13. Primer sequences used to validate the HhalOBP gene annotations. Table S14. Correspondences between H. halys predicted protein identifiers and cathepsin labels. Table S15. A total of 64 salivary effector proteins were identified in the H. halys genome. Table S16. A select subset of 15 H. halys salivary effector proteins having variable expression levels between nymphal and adult stages (up- or down-regulation). Table S17. Gene expression data for H. halys glutathione S-transferase genes. Figure S1. Phylogenetic organization of the Hemiptera. Figure S2. Ortholog distributions among hemipterans. Figure S3. Genome assembly quality control. Figure S4. Hox and Iro-C cluster gene loci. Figure S5. Halyomorpha mannosidase expansion. Figure S6. Maximum likelihood phylogenetic tree of selected mannosidase proteins from three bacter [file 12864_2020_6510_MOESM1_ESM.zip › 12864_2020_6510_MOESM1_ESM/Fig_S14__PAIRRULE.png]

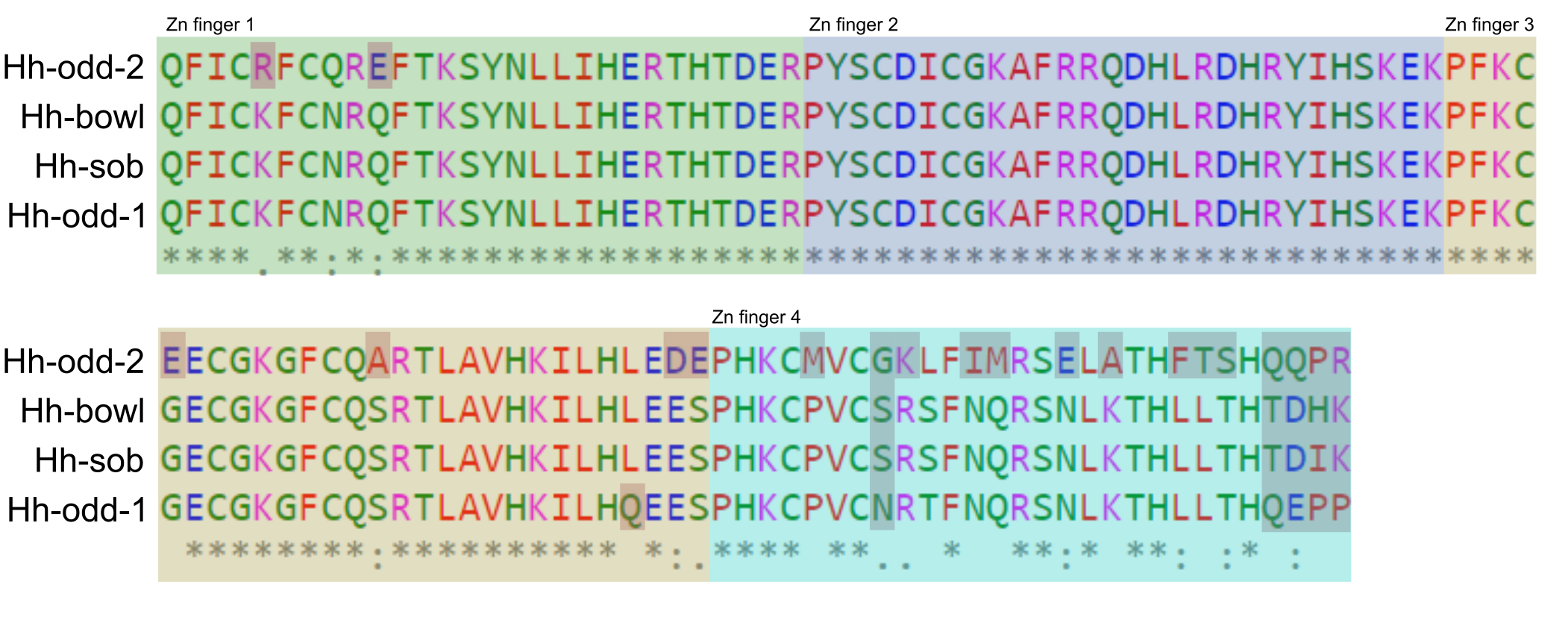

Supplement: Supplementary file 1 — Additional file 1: Main Supplementary Information text file, including Tables S1-S17 and Figures S1-S18. Table S1. Sequencing, assembly, annotation statistics and accession numbers. Table S2. OrthoDB v10 comparison of five species for ortholog presence and copy-number in Hemiptera-level orthogroups. Table S3. Scaffolds present in the H. halys assembly (accession GCA_000696795.1) that may originate from contaminant sources. Table S4. Counts of repetitive DNA elements encountered in the H. halys genome assembly. Table S5. H. halys predicted protein products associated with the RNAi pathway. Table S6. Positional information for the annotated homeobox genes. Table S7. Nuclear receptors of H. halys. Table S8. Listing of candidate Y-linked genes. Table S9. Number of genes identified as putative cuticle proteins per family in the genome of H. halys. Table S10. Number of genes identified as putative cuticle proteins per species in the genomes of several insect orders. Table S11. Clusters of genes coding for cuticle proteins in the genome of H. halys. Table S12. Odorant-binding protein genes and pseudogenes (Ψ) annotated in the genome of H. halys. Table S13. Primer sequences used to validate the HhalOBP gene annotations. Table S14. Correspondences between H. halys predicted protein identifiers and cathepsin labels. Table S15. A total of 64 salivary effector proteins were identified in the H. halys genome. Table S16. A select subset of 15 H. halys salivary effector proteins having variable expression levels between nymphal and adult stages (up- or down-regulation). Table S17. Gene expression data for H. halys glutathione S-transferase genes. Figure S1. Phylogenetic organization of the Hemiptera. Figure S2. Ortholog distributions among hemipterans. Figure S3. Genome assembly quality control. Figure S4. Hox and Iro-C cluster gene loci. Figure S5. Halyomorpha mannosidase expansion. Figure S6. Maximum likelihood phylogenetic tree of selected mannosidase proteins from three bacter [file 12864_2020_6510_MOESM1_ESM.zip › 12864_2020_6510_MOESM1_ESM/Fig_S15__ODD.png]

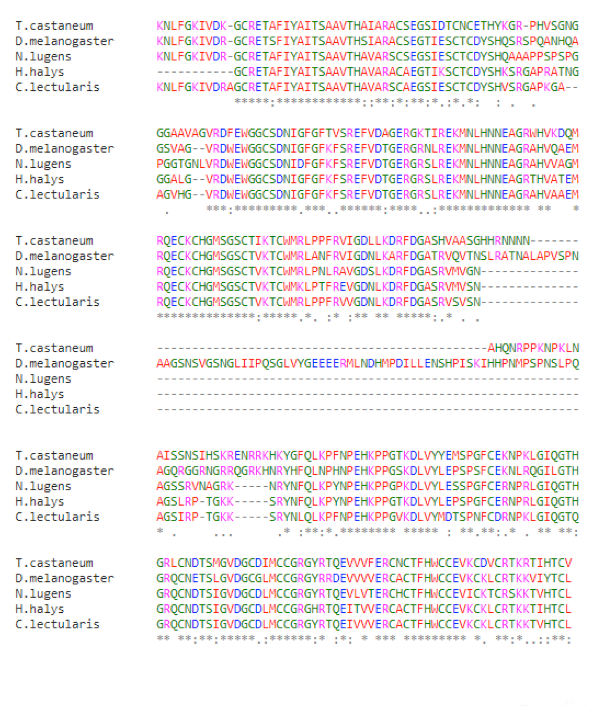

Supplement: Supplementary file 1 — Additional file 1: Main Supplementary Information text file, including Tables S1-S17 and Figures S1-S18. Table S1. Sequencing, assembly, annotation statistics and accession numbers. Table S2. OrthoDB v10 comparison of five species for ortholog presence and copy-number in Hemiptera-level orthogroups. Table S3. Scaffolds present in the H. halys assembly (accession GCA_000696795.1) that may originate from contaminant sources. Table S4. Counts of repetitive DNA elements encountered in the H. halys genome assembly. Table S5. H. halys predicted protein products associated with the RNAi pathway. Table S6. Positional information for the annotated homeobox genes. Table S7. Nuclear receptors of H. halys. Table S8. Listing of candidate Y-linked genes. Table S9. Number of genes identified as putative cuticle proteins per family in the genome of H. halys. Table S10. Number of genes identified as putative cuticle proteins per species in the genomes of several insect orders. Table S11. Clusters of genes coding for cuticle proteins in the genome of H. halys. Table S12. Odorant-binding protein genes and pseudogenes (Ψ) annotated in the genome of H. halys. Table S13. Primer sequences used to validate the HhalOBP gene annotations. Table S14. Correspondences between H. halys predicted protein identifiers and cathepsin labels. Table S15. A total of 64 salivary effector proteins were identified in the H. halys genome. Table S16. A select subset of 15 H. halys salivary effector proteins having variable expression levels between nymphal and adult stages (up- or down-regulation). Table S17. Gene expression data for H. halys glutathione S-transferase genes. Figure S1. Phylogenetic organization of the Hemiptera. Figure S2. Ortholog distributions among hemipterans. Figure S3. Genome assembly quality control. Figure S4. Hox and Iro-C cluster gene loci. Figure S5. Halyomorpha mannosidase expansion. Figure S6. Maximum likelihood phylogenetic tree of selected mannosidase proteins from three bacter [file 12864_2020_6510_MOESM1_ESM.zip › 12864_2020_6510_MOESM1_ESM/Fig_S16__WNT.png]

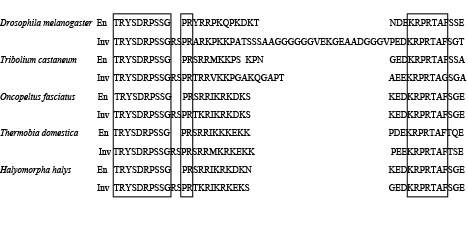

Supplement: Supplementary file 1 — Additional file 1: Main Supplementary Information text file, including Tables S1-S17 and Figures S1-S18. Table S1. Sequencing, assembly, annotation statistics and accession numbers. Table S2. OrthoDB v10 comparison of five species for ortholog presence and copy-number in Hemiptera-level orthogroups. Table S3. Scaffolds present in the H. halys assembly (accession GCA_000696795.1) that may originate from contaminant sources. Table S4. Counts of repetitive DNA elements encountered in the H. halys genome assembly. Table S5. H. halys predicted protein products associated with the RNAi pathway. Table S6. Positional information for the annotated homeobox genes. Table S7. Nuclear receptors of H. halys. Table S8. Listing of candidate Y-linked genes. Table S9. Number of genes identified as putative cuticle proteins per family in the genome of H. halys. Table S10. Number of genes identified as putative cuticle proteins per species in the genomes of several insect orders. Table S11. Clusters of genes coding for cuticle proteins in the genome of H. halys. Table S12. Odorant-binding protein genes and pseudogenes (Ψ) annotated in the genome of H. halys. Table S13. Primer sequences used to validate the HhalOBP gene annotations. Table S14. Correspondences between H. halys predicted protein identifiers and cathepsin labels. Table S15. A total of 64 salivary effector proteins were identified in the H. halys genome. Table S16. A select subset of 15 H. halys salivary effector proteins having variable expression levels between nymphal and adult stages (up- or down-regulation). Table S17. Gene expression data for H. halys glutathione S-transferase genes. Figure S1. Phylogenetic organization of the Hemiptera. Figure S2. Ortholog distributions among hemipterans. Figure S3. Genome assembly quality control. Figure S4. Hox and Iro-C cluster gene loci. Figure S5. Halyomorpha mannosidase expansion. Figure S6. Maximum likelihood phylogenetic tree of selected mannosidase proteins from three bacter [file 12864_2020_6510_MOESM1_ESM.zip › 12864_2020_6510_MOESM1_ESM/Fig_S17__RSMOTIF.png]

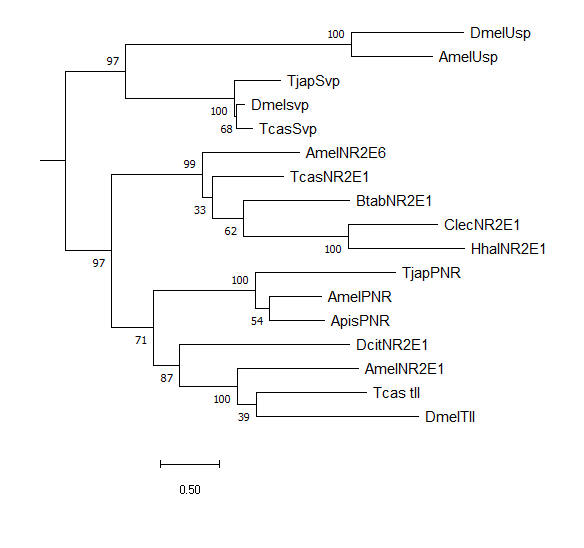

Supplement: Supplementary file 1 — Additional file 1: Main Supplementary Information text file, including Tables S1-S17 and Figures S1-S18. Table S1. Sequencing, assembly, annotation statistics and accession numbers. Table S2. OrthoDB v10 comparison of five species for ortholog presence and copy-number in Hemiptera-level orthogroups. Table S3. Scaffolds present in the H. halys assembly (accession GCA_000696795.1) that may originate from contaminant sources. Table S4. Counts of repetitive DNA elements encountered in the H. halys genome assembly. Table S5. H. halys predicted protein products associated with the RNAi pathway. Table S6. Positional information for the annotated homeobox genes. Table S7. Nuclear receptors of H. halys. Table S8. Listing of candidate Y-linked genes. Table S9. Number of genes identified as putative cuticle proteins per family in the genome of H. halys. Table S10. Number of genes identified as putative cuticle proteins per species in the genomes of several insect orders. Table S11. Clusters of genes coding for cuticle proteins in the genome of H. halys. Table S12. Odorant-binding protein genes and pseudogenes (Ψ) annotated in the genome of H. halys. Table S13. Primer sequences used to validate the HhalOBP gene annotations. Table S14. Correspondences between H. halys predicted protein identifiers and cathepsin labels. Table S15. A total of 64 salivary effector proteins were identified in the H. halys genome. Table S16. A select subset of 15 H. halys salivary effector proteins having variable expression levels between nymphal and adult stages (up- or down-regulation). Table S17. Gene expression data for H. halys glutathione S-transferase genes. Figure S1. Phylogenetic organization of the Hemiptera. Figure S2. Ortholog distributions among hemipterans. Figure S3. Genome assembly quality control. Figure S4. Hox and Iro-C cluster gene loci. Figure S5. Halyomorpha mannosidase expansion. Figure S6. Maximum likelihood phylogenetic tree of selected mannosidase proteins from three bacter [file 12864_2020_6510_MOESM1_ESM.zip › 12864_2020_6510_MOESM1_ESM/Fig_S18__NR2E.png]
